# Mapping QTLs for anaerobic tolerance at germination and bud stages using new high density genetic map of rice

**DOI:** 10.3389/fpls.2022.985080

**Published:** 2022-10-17

**Authors:** Jing Yang, Ji Wei, Jifen Xu, Yumeng Xiong, Gang Deng, Jing Liu, Shah Fahad, Hongyang Wang

**Affiliations:** ^1^ Yunnan Key Laboratory of Potato Biology, Yunnan Normal University, Kunming, China; ^2^ School of Agriculture, Yunnan University, Kunming, China; ^3^ Department of Agriculture, Abdul Wali Khan University Mardan, Khyber Pakhtunkhwa, Pakistan; ^4^ Department of Agronomy, The University of Haripur, Haripur, Pakistan

**Keywords:** direct-seeding rice, anaerobic seedling establishment, GBS, RIL, QTL mapping

## Abstract

Due to its low cost and convenience, direct seeding is an efficient technique for the production of rice in different rice growing areas. However, anaerobic conditions are a major obstacle to the direct seeding of rice and result in poor seedling establishment, which leads to yield losses. We constructed a collection of recombinant inbred lines (RIL) comprising 275 lines derived from the H335 and CHA-1 cross by the method of single seed descent. *Via* a genotyping-by-sequencing (GBS) strategy, a high-density genetic map containing 2498 recombination bin markers was constructed, the average physical distance between the markers was only 149.38 Kb. After anaerobic treatment, 12 phenotypes related to both the coleoptile at germination and seedling quality at budding were evaluated. There were no significant correlations between seedling and bud traits. Genetic mapping of quantitative traits was performed for these traits across two cropping seasons. A total of 20 loci were detected, named locus 1~20. Three of them were repeatedly detected across both seasons. Six loci overlapped with those in previous reports, and nine loci were associated with multiple traits at both stages. Notably, locus 3, which is located on chromosome 2 (26,713,837 to 27,333,897 bp), was detected for both the germination and bud traits. By focusing on the locus 3 interval and by combining gene annotation and expression analyses, we identified a promising candidate gene, trehalose-6-phosphate phosphatase (*OsTPP1*, LOC_Os02g44230). Furthermore, RILs (G289, G379, G403, G430 and G454) that have superior phenotypes and that pyramid elite alleles were recognized. The findings of present study provide new genetic resources for direct-seeding rice (DSR) varieties for molecular breeding strategies and expand our knowledge of genetic regulation of seedling establishment under anaerobic conditions.

## Introduction

Direct seeding is an energy-efficient and climate-resilient establishment technique for rice crops (Rao et al., 2007; Chauhan and Johnson, 2010). Compared with traditional transplanted-puddled rice system, direct-seeding rice (DSR) omitted transplanting process, thus saving a lot of water resources, labor and energy. Mahender et al. (2015) reported that dry DSR can save 35-57% of water. Flooding the field following direct seeding can help restrict weed growth and prevent damage from mice and birds (Manigbas et al., 2008). However, flooding also creates anaerobic conditions that affects on germination, seedling survival and morphogenesis (Yamauchi et al., 1993; Ismail et al., 2009). So, anaerobic stress has also become a limiting factor for adoption of DSR at commercial level.

In rice different strategies have been evolved to adapt the flooding stress; these strategies involve mainly two different mechanisms: the “quiescence strategy” and the “escape strategy” (Jackson, 2008; Loreti et al., 2016). A large amount of research clearly showed that, compared with those of susceptible cultivars, coleoptiles of tolerant cultivars showed higher and rapid growth under submergence during the germination phase and this adaptation in morphology allow them to arrive at surface rapidly, permitting oxygen to diffuse primary leaves and roots, to hold up seedling growth (Atwell et al., 1982; Yamauchi et al., 1993; Yamauchi and Biswas, 1997; Ismail et al., 2009). Indeed, this phenomenon is considered an “escape strategy” used to tolerate submergence during the germination phase. Therefore, these mechanisms also provide a good theoretical basis for breeding rice varieties that have enhanced capabilities for anaerobic germination (AG).

Unlike most cereal crop species, under O_2_ deficiency, rice can mobilize starchy endosperm as easily fermentable sugars to produce ATP; the phenomenon is important for supporting AG (Guglielminetti et al., 1995; Hwang et al., 1999). When the seeds germinate under submerged conditions, mitochondria release Ca^2+^ as secondary messenger in response of O_2_ deficiency and sugar starvation. Calcineurin B-like (CBL) is a Ca^2+^ sensor which binds and activated by Ca^2+^. Activated CBL/Ca^2+^ activates SnRK1A by interacting with protein kinase CIPK15 which results in activation of the promoter of the transcription factor MYBS1 and phosphorylates the MYBS1 protein. Moreover, gibberellic acid (GA) synthesized by the embryo diffuses to aleurone cells and induces expression of the transcription factor MYBGA. Sugar response elements (SREs) and GA response element (GAREs) are two important control elements. MYBGA binds to GAREs while, MYBS1 binds to SREs, developing a bipartite MYB-DNA complex that considerably activates α-amylase gene promoters (Hong et al., 2012). The hydrolysis of starch stored within the rice seeds then provides energy for germination,*OsTPP7* encoding trehalose-6-phosphate (T6P) phosphatase, has been involved in T6P metabolic processes and functions to enhance starch mobilization for higher tolerance of AG (Kretzschmar et al., 2015). However, *OsTPP7* signaling pathway is still unclear and additional research is needed.

To date, genetic research on the germination of rice under O_2_ deficient conditions have focused majorly on mining functional genes and exploring their mechanisms of function. Some QTLs are identified by genetic mapping populations. Several studies have considered coleoptile elongation an indicator trait of tolerant phenotypes for QTL mapping. Seven QTLs associated with seed anoxia germinability were mapped onto chromosomes 1, 2, 5, and 7 *via* QTL mapping of two populations (Jiang et al., 2004; Jiang et al., 2006). Using the seeding survival rate as an indicator of tolerant phenotypes, Angaji. (2008); Anganji et al., (2010) investigated two different backcross inbred line populations, both of which were derived from the backcrossed offspring of IR64 as a recurrent parent, to map a total of 13 QTLs throughout the chromosomes. Septiningsih et al. (2013) identified six QTLs that were significantly associated with survival rate on chromosomes 2, 5, 6, and 7. Baltazar et al. (2014) identified four QTLs that contributed to anaerobic tolerance *via* improved survival rates. Kim and Reinke (2018) reported four QTLs responsible for survival rate to a certain extent on chromosomes 1, 8, and 11. Four QTLs derived from Kharsu 80A giving enhanced tolerance in the absence of free oxygen germination were identified by Baltazar et al. (2019): three on chromosome number 7 and one on chromosome number 3, phenotypic variance clarified from 8.1% to 12.6%. When two biparental mapping populations were used for QTL analyses, this identified four QTLs on chromosomes 1, 3 and 7 for seedling height and five QTLs on chromosomes 3, 5, 6, 7, and 8 for survival rate (Ghosal et al., 2019). Recently, Kong et al. (2022) identified two QTLs on chromosomes 1 (*qCL-1.1*) and 3 (*qCL-3.1*) using for coleoptile length as an indicator. Genome-wide association studies (GWASs) represent an efficient approach for complex traits dissection, however, little literature on excavation of loci linked with AG tolerance *via* GWASs is available. Hsu and Tung (2015) performed GWAS by 36901 single nucleotide polymorphisms (SNPs) in addition to find 88 genetic loci linked to AG tolerance. *Via* GWAS of the 5291 SNP markers in the 432 *indica* varieties, 15 genetic loci linked with AG tolerance were detected (Zhang et al., 2017). Fifty genes for submergence stress were identified when Gao et al. (2020) combined information of GWAS, transcriptomic analysis, and reported QTL locations. Su et al. (2021) conducted genome resequencing on 209 rice varieties, and a dynamic GWAS of coleoptile length and diameter was adopted, with 26 loci were detected.

Although some progress has been made in QTL analyses for AG tolerance, relatively few genetic loci associated with AG tolerance compared with other important rice traits have been identified. To date, due to a low density of markers, only *qAG-9-2* finely mapped and then cloned as the *OsTPP7* (Kretzschmar et al., 2015). This has occurred because most of these genetic loci were recognized based on traditional markers, which are sparsely distributed across 12 rice chromosomes; in addition, only two main indicators have been used for mapping. These two reasons lead to the low efficiency of the excavation of genetic loci associated with AG tolerance and limit our knowledge of genetic regulatory mechanisms of AG tolerance in the seeds of rice. Therefore, the development of new types of high-density markers and additional high-efficiency phenotypic indicators are essential to assist fine mapping and cloning of QTLs.

In our previous study, we based on the protocol of Septiningsih et al. (2013), investigated the seedling survival rateof rice seeds after twenty one days of germination in water 10cm deep of 200 conventional *indica* rice varieties mainly from Guangdong province, China. The average seedling survival rate of 200 materials was 42.67%, the maximum was 83.00%, and the minimum was 8.13%. Interestingly, the seedling survival rate of our donor parent H335 was 78.12%, while another parent CHA-1 was only 8.85% (unpublished data). Therefore, we created RIL population included 275 RILs derived *via* the single seed descent from CHA-1 and H335. The GBS approach was applied to sequence 275 recombinant inbred lines (RILs), generating a set of high-density SNP markers. To create linkage map of high density depending on the technique of parent independent genotyping constructing linkage map (Xie et al., 2010) and validate it using genes from the reference genome. We evaluated 12 traits of rice seeds after 6 days anaerobic treatment at the germination and bud stages; these 12 traits can be used for the comprehensive evaluation of the anaerobic seedling establishment ability of rice. QTL mapping was performed based on these 12 traits to identify additional genetic loci to enrich the genotypes of genetic pool associated with anaerobic seeding tolerance. These results broaden the knowledge of the anaerobic tolerance genetic mechanisms of rice seeds at germination and bud stages. Specifically some QTLs are promising targets for the marker assisted breeding of DSR varieties.

## Materials and methods

### Plant materials

In present study, National Engineering Research Center for Plant Space Breeding bred two parents H335 and CHA-1. RIL population of 275 lines were constructed using single seed descent method. Both the parents and RILs were grown in rice field during wet (WS) and dry season (DS) of 2017 at the South China Agricultural University, Province Guangzhou, China (113°E longitude, 23°N latitude approximately). Block design was used to grown each RIL or parent, comprising 6 columns × 6 rows, with 20 cm space between the plants. Keeping in view the issues related to seed maturity, 6 plants were harvested independently from the center of each block on 35^th^ and 40^th^ day after heading during WS and DS respectively. Seeds obtained after harvest were then dried at 42°C in dryer using heated air for five days then finally stored at low temperature (-20°C).

### Evaluation of anaerobic tolerance at germination and bud stages

After harvesting 3 out of 6 plants per line per season with healthy seeds were chosen. Their seeds were kept in an oven at 50°C for the period of one week to break their dormancy, then sterilized with a 20% diluted bleach solution (6-7% NaClO) for 20 minutes and finally sterile water was used to rinse the seeds. For the germination stage, five seeds were added to each centrifuge tube of 10 cm and filled with the distilled water to develop anaerobic conditions. Four replicates (centrifuge tubes) were included per individual, and twelve replicates were included per line. For the bud stage, three individuals were selected per line, approximately 100 seeds per individual were placed in a petri dish (9 cm) randomly, and 10 ml of distilled water was poured. These petri dishes were positioned in a chamber of 8 hour light (200 μmol m^-2^ s^-1^) and 16 h dark cycle at 30°C. Germinated seeds with approximately 3-mm-long buds were selected, and five seeds were positioned to the bottom of 1 tissue culture bottle (75 mm diameter, 108 mm height) that was filled with distilled water. Two replicates (tissue culture bottles) were included per individual, and a total of six replicates were included per line. All the centrifuge tubes and tissue culture bottles were placed immediately in a chamber where a shift of light 8 h (200 μmol m^-2^ s^-1^) and dark16 hour cycle was maintained at the temperature of 30°C. Coleoptile volume (CV), coleoptile surface area (CSA), coleoptile length (CL) and the coleoptile diameter (CD) of germination stage, shoot height (SH), shoot stem diameter (SSD), root length (RL), root volume (RV), root surface area (RSA), and root diameter (RD)of bud stage were determined after six days using WinRHIZO root image analysis system. Shoot fresh biomass (SFW) and root fresh biomass (RFW) were calculated using sensitive balance. For the statistical analysis Statistical Analysis System and Microsoft Excel were used. The statistical significance of the difference between two parents was evaluated by One-way ANOVA. The correlations of traits were computed using PROC CORR by SAS software.

### DNA extraction, library construction and illumina sequencing

Leaf samples were collected from the 275 RILs in the F_7_ generation. Leaf tissues were used to extract total genomic DNA using the CTAB method. The quantity and quality of genomic DNA was determined by NanoDrop ND-1000 Spectrophotometer and 1% agarose gel electrophoresis, respectively (Thermo Scientific, Wilmington, USA).

For 275 RILs, the genomic DNA was incubated with ATP (NEB), T4 DNA ligase (NEB), *MseI* (New England Biolabs, NEB), and *MseI* Y adapter N containing barcode at 37°C. Temperature at 65°C was used to inactivate restriction ligation reactions and digested at 37°C by the additional restriction enzyme (*NlaIII*). Agencourt AMPure XP (Beckman) was utilized to purify samples of the restriction ligation. Finally PCR was done with these purified samples, universal primers of Phusion Master Mix (NEB), index primers to add index, as well as entire i5 and i7 sequences. Purification of products of PCR was carried out by Agencourt AMPure XP (Beckman) then pooled and run on an agarose gel (2%). Gel extraction kit (Qiagen) was utilized to separate fragments of 375-400 bp (with indexes and adaptors) in size. The fragment products were subsequently purified by using Agencourt AMPure XP (Beckman) and diluted to further sequencing. After that paired-end sequencing was done on the selected tags with Illumina HiSeq PE150 platform (Novogene Bioinformatics Technology Co., Ltd, P.R. China).

### SNP identification and bin marker production

Sequencing give original image data which were converted into sequence data (raw data) in fastq format *via* base calling (Illumina pipeline CASAVA version 1.8.2). Firstly raw data were processed by a chain of quality control (QC) methods: (1) removing reads with ≥ 10% unidentified nucleotides (N), (2) removing reads with > 50% bases having a phred quality < 5, and (3) removing reads with > 10 nt aligned to adapter, allowing ≤ 10% mismatches. Burrows-Wheeler Aligner version 0.7.8 was utilized to align clean reads of every sample against reference genome (MSU Rice Genome Annotation Project database v.7; http://rice.plantbiology.msu.edu/); command line was ‘BWA mem -t 4 -k 32 -M’. After alignment, by means of a Bayesian approach implemented in package SAMtools, we performed SNP calling on population scale (Li et al., 2009). According to the methods Xie et al. (2010), filtered abnormalities, identified biallelic homozygous SNPs. I.e. all potential SNPs were identified in the entire population to obtain drafts of parental genotypes using a maximum parsimonious inference of recombination. Then, filtering out low-quality SNPs by Bayesian inference. Finally, RILs were genotyped using high quality SNPs assisted by a hidden Markov model.

In accordance with the methods of Huang et al. (2009), with some modifications, RILs genotypic maps were aligned and split in recombination bins following breakpoints, with a window size parameter of 15 SNPs. In addition, their genotypes were compared for a 100-kb interval. Adjacent 100-kb intervals with similar genotype across all RILs were combined into a recombination bin marker. Networks were visualized by Cytoscape version.3.6.1. (Smoot et al., 2011).

### Linkage map construction and QTL analyses

Bin markers utilized to construct genetic linkage map by the R/qtl package (Broman et al., 2003) with Kosambi map method, and genetic distances of markers were determined. QTL analyses were carried out using QTL IciMapping v4.1 (Meng et al., 2015) software. Significance threshold value of logarithm of odd (LOD) scores was 2.5 for the QTL detection, and QTL additive effect and contribution rate to trait were determined.

### Validation of the candidate genes by the real-time quantitative RT-PCR

According to the method of Yang et al. (2019). Total RNA of every RILs were homogenized by mortar and pestle using the liquid nitrogen and after that purified with a Purification Kit of Plant Total RNA (ComWin Biotech Company) following the manufacturer’s instructions. Samples of RNA undergo reverse transcription process to develop cDNA by means of high capacity cDNA archive kit (Applied Biosystems, USA). AceQ qPCR SYBR Green Master Mix Kit (Vazyme Biotech) was utilized to conduct qRT-PCR following standard protocol. Furthermore, StepOnePlus System (Applied Biosystems, USA) was utilized to estimate genes expression levels. Each treatment has three replications. Being endogenous control, the Actin was utilized in normalization of cycle threshold (Ct) value achieved, and ΔΔCt method was applied to determine values for relative expression NCBI primer BLAST (http://www.ncbi.nlm.nih.gov/tools/primer-blast/) were utilized to design gene-specific primers and primer sequences of four candidate genes listed in **Table S1**.

## Results

### Sequencing and genotyping

Sequencing of the GBS libraries yielded approximately 110.95 Gb of raw data for the 275 RILs, with an average of 0.40 Gb for each line. The Q30 ratio for the entire samples ranged from 86.89 to 96.12%, with a mean of 92.14%; thus, data quality is high and meets the necessities for further analysis. After the raw data were filtered strictly, a total of 770,428,068 clean paired-end reads were obtained. Approximately 96.79% of reads were mapped to Nipponbare reference genome. The mapped regions were covered by the captured fragments totaled approximately 12.16% of genome sequence with coverage depth of 14.14× on average for captured regions (**Table S2**).

To identify potential SNP sites by the use of sequences of RILs. Genotype calling was performed for each RIL, the detected SNPs are merged into a set, which contains 805,088 SNPs. According to the methods and criteria described in detail by Xie et al. (2010), after filtering for abnormalities, 100307 biallelic homozygous SNPs were validated for the estimation of the recombinant event.

### Bin map construction and characteristics

**Figure 1:**
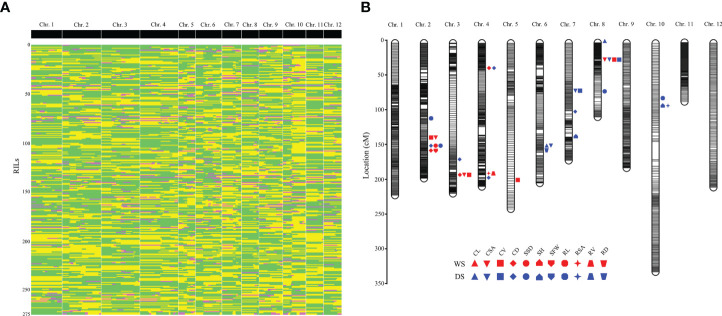
Genetic linkage map constructed with bin markers and the locations of QTLs associated with traits related to anaerobic seedling establishment. **(A)** Recombination bin map consisting of 2,498 bin markers. Green: CHA-1 genotype; yellow: H335 genotype; pink: heterozygote. **(B)** All of the QTL positions on the high-density map. The red patterns represent the QTLs detected during the wet season (WS); the blue patterns represent the QTLs detected during the dry season (DS). The words associated with different shapes are abbreviations of different phenotypes: CL, coleoptile length; CSA, coleoptile surface area; CV, coleoptile volume; CD, coleoptile diameter; SSD, shoot stem diameter; SH, shoot height; SFW, shoot fresh weight; RL, root length; RSA, root surface area; RV, root volume; RD, root diameter.

For the RIL population, adjacent 100-kb intervals with same genotype across all 275 RILs were identified as single recombination bin marker (Huang et al., 2009). Finally, 2,498 recombination bin throughout 12 chromosomes adopted to develop genetic map for RIL population (**Figure 1A**). Map spanned a total of 2371.84 cM, with a mean interval of 0.95 cM between adjacent markers (**Table 1** and **Figure 1**). Among the 12 chromosomes, chromosome 2 was highly saturated; it include 316 markers, with 0.62 cM average marker density. In contrast, chromosome 10 was the least dense with 1.77 cM average marker density. Approximately 197.65 cM was an average genetic size for 12 chromosomes while, 149.38 kb was an average physical distance between the markers.

**Table 1:** Distribution of genetic markers across the 12 chromosomes in rice.

Chromosome	Number of markers	Total Distance (cM)	Average Genetic Distance between Markers (cM)	Gap < 5 cM	Average Physical Distance between Markers (kb)
Chr. 1	251	219.89	0.88	100.00%	172.38
Chr. 2	316	196.26	0.62	99.37%	113.70
Chr. 3	313	217.4	0.70	99.68%	116.33
Chr. 4	309	207.84	0.67	99.68%	114.77
Chr. 5	138	238.62	1.74	99.27%	217.08
Chr. 6	212	202.72	0.96	99.05%	147.38
Chr. 7	156	171.16	1.10	99.35%	190.26
Chr. 8	141	111.26	0.79	99.29%	201.70
Chr. 9	192	181.65	0.95	100.00%	119.85
Chr. 10	185	326.27	1.77	98.37%	125.44
Chr. 11	140	89.95	0.65	100.00%	207.25
Chr. 12	145	208.82	1.45	100.00%	189.86
Overall	2,498	2,371.84	0.95	99.51%	149.38

### Bin map construction and characteristics

Collinearity analysis was performed by genetic map and physical location of the 2,498 bin markers. The spearman correlation coefficient was calculated for all the linkage groups. Its value near to 1 showed an improved collinearity among physical and genetic map. Order of most markers on every chromosome of the project is consistent with genome, indicating that collinearity is good and calculation accuracy of genetic recombination rate is high (**Figure S1**). To evaluate the accuracy and power of present genetic map for traits, a QTL mapping of heading date was carried out. The QTL *qHD-8*, for which peak encompassed a cloned gene involved in heading date (*Hd18*) (Shibaya et al., 2016), was detected on chromosome 8, and this QTL had a high LOD value of 11.98 (**Figure S2**). *qHD-8*, which is located within the genetic interval from block12804~block12814, can explain 15.57% of the phenotypic variation. The physical distance between the *Hd18* gene and  block12814 on chromosome 8 is only 77 kb.

### Phenotypic performance of RIL population for anaerobic tolerance

**Figure 2:**
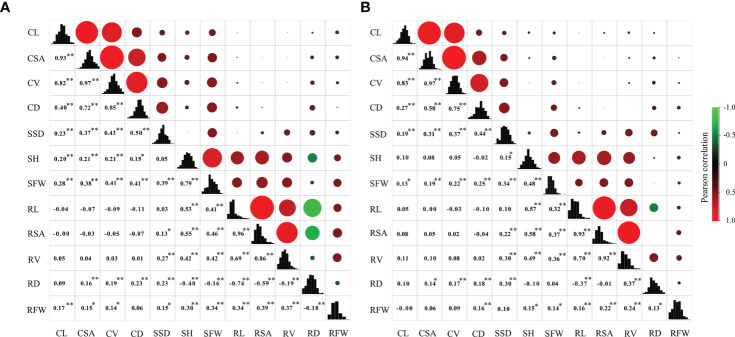
Correlation coefficients among 12 traits at the germination and bud stages under anaerobic stress in the RIL population. In the upper panel, the size of circle and depth of shading indicate magnitude of correlations. Negative correlations are colored green, and positive correlations are colored red. Lower panel contains correlation coefficients, and ^*^ and ^**^ represent significance at 0.05 and 0.01, respectively. The diagonal represents frequency distribution of 12 traits. **(A)** wet season; **(B)** dry season.

We investigated 4 traits (CL, CV, CD, CSA), at the germination stage and 8 traits (SH, SSD, RL, SFW, RV, RSA, RFW, RD) at the bud stage in the RIL population under anaerobic conditions. Variations in both the parents are obvious (**Table 2**). As for RILs population, single peak pattern distributions have examined for 12 investigated traits, and these distributions widely varied (**Figure 2**; **Tables 2**, **S3**).

**Table 2:** Phenotypic performance during the germination and the bud stage under anaerobic stress across two cropping seasons.

Trait^a^	Env^b^	Parents^c^		RIL population				
		CHA-1	H335	Mean ± SD	Range	Skewness	Kurtosis	*CV* ^d^(%)
CL (cm)	WS	1.770 ± 0.146	2.825 ± 0.216^**^	2.737 ± 0.254	1.776-3.400	0.006	0.311	9.266
	DS	2.102 ± 0.193	2.657 ± 0.512	2.562 ± 0.349	1.421-3.608	-0.189	0.265	13.604
CSA (cm^2^)	WS	0.303 ± 0.007	0.556 ± 0.042^**^	0.507 ± 0.062	0.297-0.652	-0.006	0.151	12.238
	DS	0.330 ± 0.034	0.542 ± 0.036^**^	0.462 ± 0.075	0.269-0.710	0.123	0.033	16.271
CV (mm^3^)	WS	4.200 ± 0.557	7.644 ± 0.840^**^	7.509 ± 1.220	4.000-10.668	0.063	0.047	16.245
	DS	4.640 ± 0.289	7.381 ± 0.863^**^	6.658 ± 1.353	3.359-11.142	0.268	-0.078	20.222
CD (mm)	WS	0.540 ± 0.030	0.620 ± 0.030^*^	0.588 ± 0.031	0.494-0.670	-0.188	0.211	5.198
	DS	0.500 ± 0.007	0.577 ± 0.021^*^	0.572 ± 0.034	0.482-0.662	-0.238	-0.277	5.939
SSD (mm)	WS	0.614 ± 0.075	0.713 ± 0.026^*^	0.699 ± 0.050	0.560-0.844	-0.018	0.115	7.119
	DS	0.580 ± 0.058	0.785 ± 0.008^**^	0.668 ± 0.049	0.511-0.818	-0.161	0.130	7.307
SH (cm)	WS	4.400 ± 0.265	5.340 ± 0.560	4.930 ± 1.014	2.289-7.763	-0.053	-0.241	20.562
	DS	3.730 ± .0680	5.285 ± 0.390^*^	4.687 ± 0.770	2.773-7.812	0.313	0.690	16.420
SFW (mg)	WS	10.871 ± 1.813	6.862 ± 2.415^**^	15.867 ± 2.815	9.243-29.390	0.386	1.251	17.754
	DS	11.108 ± 1.062	18.313 ± 1.429^**^	14.904 ± 3.399	8.300-53.798	5.646	61.418	22.808
RL (cm)	WS	2.347 ± 0.095	3.590 ± 0.152^**^	3.114 ± 1.212	1.316-9.357	1.852	4.462	38.919
	DS	2.160 ± 0.321	3.244 ± 0.276^*^	2.425 ± 0.604	1.522-6.391	2.450	10.858	24.948
RSA (cm^2^)	WS	0.332 ± 0.031	0.424 ± 0.088	0.415 ± 0.107	0.222-0.874	1.259	2.348	25.742
	DS	0.350 ± 0.040	0.558 ± 0.030^**^	0.407 ± 0.091	0.267-1.079	2.484	12.156	22.248
RV (mm^3^)	WS	3.560 ± 0.288	4.541 ± 0.186^**^	4.415 ± 0.903	2.710-9.083	0.953	2.484	20.464
	DS	4.930 ± 0.030	6.916 ± 0.440^**^	5.497 ± 1.334	3.583-14.833	2.564	11.083	24.254
RD (mm)	WS	0.423 ± 0.199	0.477 ± 0.020	0.454 ± 0.043	0.299-0.568	-0.266	0.505	9.389
	DS	0.510 ± 0.048	0.568 ± 0.122	0.541 ± 0.045	0.429-0.735	0.752	2.073	8.344
RFW (mg)	WS	4.802 ± 0.040	7.225 ± 0.030^**^	6.548 ± 2.651	3.125-28.427	4.631	29.623	40.487
	DS	5.548 ± 0.100	8.241 ± 0.120^**^	6.855 ± 3.184	4.495-36.327	6.531	47.883	46.447

a Trait: CL, coleoptile length; CSA, coleoptile surface area; CV, coleoptile volume; CD, coleoptile diameter; SSD, shoot stem diameter; SH, shoot height; SFW, shoot fresh weight; RL, root length; RSA, root surface area; RV, root volume; RD, root diameter; RFW, root fresh weight.

b Environment: WS is the wet season in 2017; DS is the dry season in 2017.

c Parent refers to the mean ± standard deviation (SD) of the parents, * and ** indicates significance at the levels of 0.05 and 0.01, respectively.

d *CV* (%), coefficient of variation.

The correlations among the 12 traits in RIL population were examined (**Figure 2**). Results revealed no strong correlations exist among traits at germination and bud stages and that the correlation coefficient between CD and SSD was the greatest 0.50 and 0.44 during WS and DS, respectively. For four traits at the germination stage, there were significant correlations between the four traits, except the correlation between CL and CD was not strong. For the eight traits at bud stage, no strong correlation exist between the eight traits, except RSA was strongly correlated with RL and RV.

### QTL mapping in the RIL population

**Figure 3:**
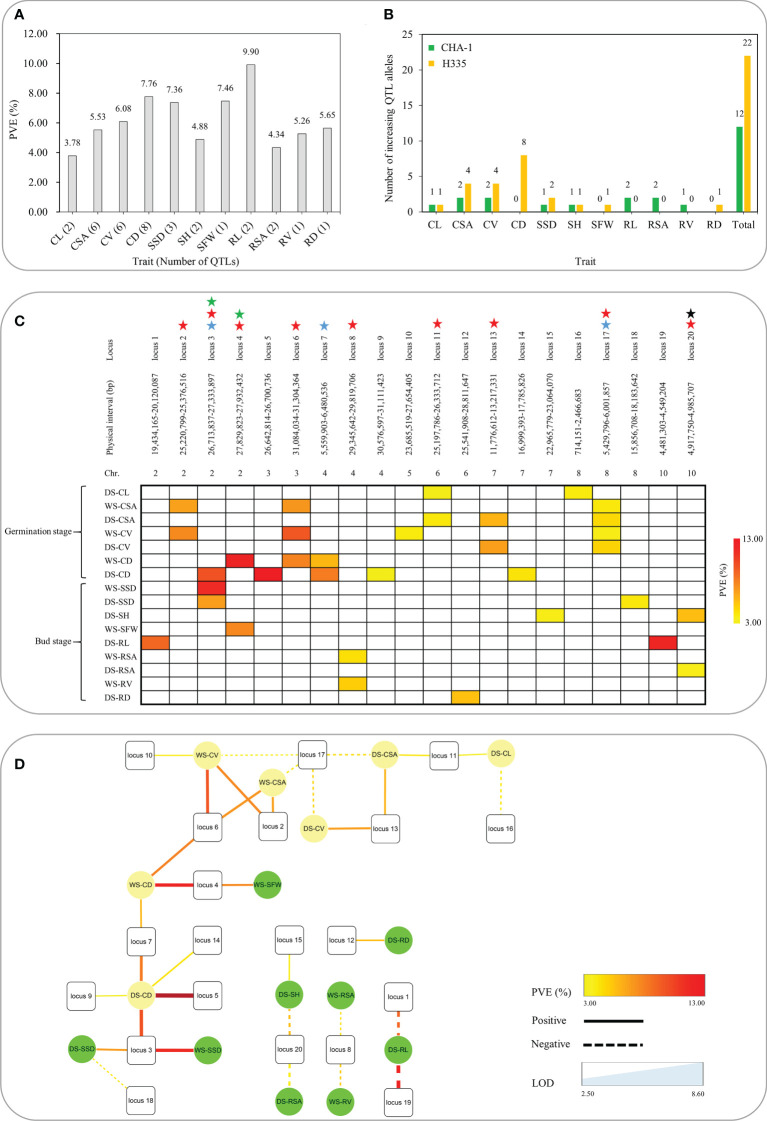
Summary of 20 loci associated with 11 traits related to anaerobic tolerance. **(A)** Phenotypic contribution rates of QTLs associated with 11 traits. **(B)** Number of increasing QTL alleles in various traits contributed by parents. **(C)** Heat map reveals pleiotropy of various QTLs. The different colors shown in the legend correspond to various levels of explanation of phenotypic variation. The blue star means detected in both seasons, the red star represents the simultaneous influence of multiple traits, the black star represents the trait that affects both shoots and roots for bud growth, and the green star indicates that both seed germination and bud growth are affected. **(D)** The interaction network of different traits and QTLs. The square represents locus, the circle represents trait; the dotted line represents the negative effect, the solid line represents the positive effect; the thickness of the line represents the LOD value, and the color of the line represents the PVE.

Via method of inclusive composite interval mapping (ICIM), 34 QTLs (14 and 20 for the WS and DS, respectively) by IciMapping v4.1 software. Phenotypic difference explained (PVE) *via* the QTLs were ranged 3.34-12.17%. Among them, 22 and 12 were detected at germination and bud stages, respectively, and they are distributed on chromosomes 2, 3, 4, 5, 6, 7, 8, and 10 (**Figure 1B**). QTLs were detected for all traits except RFW. The most QTLs (eight) were associated with CD. However, there was only one QTL each associated with SFW, RV and RD (**Table 3**). The average PVE of different traits was calculated, in which the CL was the lowest (3.78%) and the RL was the highest (9.90%) (**Figure 3A**). Both the parents contributed favorable alleles at different loci, while, among the 34 QTLs, 22 were from H335, and only 12 were from CHA-1 (**Figure 3B**). This observation is consistent with phenotypic differences observed between two parents.

**Table 3:** QTLs that are associated with anaerobic tolerance at the germination and bud stages and that were detected in the RIL population.

Locus	QTL	Env^a^	Chr.^b^	Marker interval	Physical interval (bp)	LOD^c^	PVE (%)^d^	ADD^e^	Known loci
Locus 1	*qRL-2*	DS	2	Block3371-Block3403	19,434,165-20,120,087	5.48	8.63	-0.7003	
Locus 2	*qCV-2*	WS	2	Block3775-Block3787	25,220,799-25,376,516	4.74	7.40	0.3058	(*qAG-2*, Jiang et al., 2004)
	*qCSA-2*	WS	2	Block3780-Block3787	25,304,101-25,376,516	4.31	6.61	0.0154	
Locus 3	*qSSD-2*	WS	2	Block3943-Block3970	26,713,837-27,333,897	6.58	10.89	0.0162	(*qAG-2*, Jiang et al., 2004; *qSAT-2-B*,Wang, 2009; *qAG2*, Septiningsih et al., 2013)
	*qSSD-2*	DS	2	Block3943-Block3970	26,713,837-27,333,897	3.86	6.77	0.0121	
	*qCD-2-1*	DS	2	Block3943-Block3968	26,713,837-27,298,326	6.73	9.35	0.0094	
Locus 4	*qCD-2-2*	WS	2	Block3988-Block3998	27,829,823-27,932,432	6.60	11.28	0.0092	(*qAG-2*, Jiang et al., 2004 *qSAT-2-B*,Wang, 2009; *qAG2*, Septiningsih et al., 2013)
	*qSFW-2*	WS	2	Block3988-Block3998	27,829,823-27,932,432	4.07	7.46	0.0007	
Locus 5	*qCD-3-1*	DS	3	Block5779-Block5782	26,642,814-26,700,736	8.60	12.17	0.0108	
Locus 6	*qCD-3-2*	WS	3	Block6004-Block6012	31,084,034-31,304,364	4.60	7.66	0.0076	
	*qCSA-3*	WS	3	Block6009-Block6012	31,220,745-31,304,364	4.63	7.00	0.0160	
	*qCV-3*	WS	3	Block6009-Block6012	31,220,745-31,304,364	5.81	9.14	0.3425	
Locus 7	*qCD-4-1*	WS	4	Block6696-Block6755	5,559,903-6,480,536	3.51	5.88	0.0067	
	*qCD-4-1*	DS	4	Block6696-Block6755	5,559,903-6,480,536	5.73	7.91	0.0086	
Locus 8	*qRSA-4*	WS	4	Block8028-Block8047	29,345,642-29,819,706	2.75	4.63	-0.0231	
	*qRV-4*	WS	4	Block8028-Block8047	29,345,642-29,819,706	3.19	5.26	-0.2099	
Locus 9	*qCD-4-2*	DS	4	Block8102-Block8143	30,576,597-31,111,423	2.51	3.36	0.0056	
Locus 10	*qCV-5*	WS	5	Block9860-Block9980	23,685,519-27,654,405	2.50	4.21	0.0068	
Locus 11	*qCL-6*	DS	6	Block11298-Block11400	25,197,786-26,333,712	2.55	3.34	0.0683	
	*qCSA-6*	DS	6	Block11298-Block11400	25,197,786-26,333,712	2.85	4.37	0.0154	
Locus 12	*qRD-6*	DS	6	Block11313-Block11557	25,541,908-28,811,647	3.18	5.65	0.0104	
Locus 13	*qCSA-7*	DS	7	Block12037-Block12088	11,776,612-13,217,331	3.90	5.99	0.0180	(*qAG7-1*, Angaji et al., 2010)
	*qCV-7*	DS	7	Block12037-Block12088	11,776,612-13,217,331	4.12	6.68	0.3275	
Locus 14	*qCD-7*	DS	7	Block12247-Block12295	16,999,393-17,785,826	3.32	4.49	0.0065	(*qAG7.2*, Septiningsih et al., 2013; *qAG7*, Baltazar et al., 2014; Hsu and Tung, 2015)
Locus 15	*qSH-7*	DS	7	Block12476-Block12480	22,965,779-23,064,070	2.99	4.08	0.1625	
Locus 16	*qCL-8*	DS	8	Block12756-Block12814	714,151-2,466,683	3.22	4.23	-0.0776	
Locus 17	*qCSA-8*	WS	8	Block13087-Block13127	5,429,796-6,001,857	2.94	4.41	-0.0127	(*qSAT-8-B*, Wang, 2009; *qGS8*,Chen et al., 2012; Hsu and Tung, 2015)
	*qCSA-8*	DS	8	Block13087-Block13127	5,429,796-6,001,857	3.14	4.80	-0.0163	
	*qCV-8*	WS	8	Block13087-Block13127	5,429,796-6,001,857	2.67	4.09	-0.2291	
	*qCV-8*	DS	8	Block13087-Block13127	5,429,796-6,001,857	3.09	4.97	-0.2855	
Locus 18	*qSSD-8*	DS	8	Block13593-Block13899	15,856,708-18,183,642	2.54	4.41	-0.0097	
Locus 19	*qRL-10*	DS	10	Block16606-Block16617	4,481,303-4,549,204	7.62	11.18	-1.0452	
Locus 20	*qSH-10*	DS	10	Block16658-Block16661	4,917,750-4,985,707	4.12	5.68	-0.1928	
	*qRSA-10*	DS	10	Block16658-Block16661	4,917,750-4,985,707	4.50	4.05	-0.0928	

a Env., WS is wet season in 2017; DS is dry season in 2017.

b Chr., chromosome.

c LOD, logarithm of odds.

d PVE (%), phenotypic variation explained (%).

e ADD, additive effect; positive values indicate the superiority of H335.

QTLs which overlapped due to physical position were categorized as same locus. Eventually, a total of 20 loci were found (**Table 3**). Remarkably, 3 out of 20 loci were repetitively identified across the two seasons: locus 3 (*qCD-2-1*, DS; *qSSD-2*, WS and DS), locus 7 (*qCD-4-1*, WS and DS), and locus 17 (*qCSA-8*, WS and DS; *qCV-8*, WS and DS). To further authenticate the correctness these results, loci of present study were compared with reported QTLs previously identified by linkage or association mapping approach. We discovered that up to six loci have previously been reported. Locus 2 associated with CV and CSA, was detected within genomic interval of *qAG-2*, was shown to be related to the length of shoots, including the coleoptile (Jiang et al., 2004). Multiple reported QTLs were clustered at the end of chromosome 2 (approximately 27 Mb). Specifically, *qAG-2* (Jiang et al., 2004), *qSAT-2-B* (Wang, 2009) and *qAG2* (Septiningsih et al., 2013) were shown to be associated with shoot length, the anaerobic response index and survival rate, respectively. We found two loci within this interval: locus 3, which is associated with SSD and CD, and locus 4, which is associated with SFW and CD. On the chromosome 7, locus 13, which is associated with CSA and CV, mapped to genomic region of *qAG7-1*. Another locus, locus 14, which is associated with CD, colocalized with a reported locus detected by the use of both linkage mapping as well as association mapping approaches (Septiningsih et al., 2013; Baltazar et al., 2014; Hsu and Tung, 2015). In addition, locus 17 on chromosome 7 overlapped with a reported locus that was also detected by the use both linkage mapping and GWAS approaches (Wang, 2009; Chen et al., 2012; Hsu and Tung, 2015).

### Genetic loci pleiotropy

Gene pleiotropy is a common phenomenon in plant genetics. A matrix summarizing of all the QTLs associated with 11 traits associated to anaerobic tolerance at the germination and budding is shown in **Figure 3C**. In our study, nine of the 20 loci were associated with multiple traits at both stages. Among these nine loci, five associated with multiple traits only at the germination stage. Specifically, locus 2, 13 and 17 were associated with both CSA and CV; locus 6 was associated with CSA, CV and CD; and locus 11 was linked with CL and CSA. Two loci associated with multiple traits only at the bud stage. Specifically, locus 8 was associated with both RV and RSA, and locus 20 was associated with both SH and RSA, indicating that locus 20 affects both the aboveground and the belowground traits of seedlings at the bud stage. In addition, there are two loci affecting traits at both the germination and budding; i.e., locus 3 was linked with CD at germination and SSD at bud stage. Locus 4 was associated with CD and SFW at the germination and bud stage, respectively. Notably, these results were also supported by Pearson correlation analyses based on traits measured at both stages in the two environments (**Figure 2**). Moreover, to further recognize the action relationship between these genetic loci as well as different traits, we constructed the action network between QTLs and traits (**Figure 3D**). The locus 3, 6 and 17 had higher connectivity, which were the ideal gene donor for direct seeding rice breeding. In summary, these QTLs will help us efficiently select rice varieties suitable for direct seeding.

### Screening materials that pyramid elite alleles

In general, some degree of transgressive segregation may occur in progeny due to elite parental alleles that sufficiently recombined in offspring. We determined the source of the elite alleles according to their additive effect value. The distribution of these 20 loci in individual RILs was analyzed in detail (**Table S4** and **Figure S3**). Most individuals harbored six to ten elite alleles. The fewest harbored only one elite allele, and the most harbored up to 16 favorable alleles. Combined with the phenotypes across both seasons, we selected five RILs (G289, G379, G403, G430 and G454) that exhibited excellent phenotypes and that can serve as favorable alleles donor parents during breeding process.

### Identification of the candidate genes from reliable locus

**Figure 4:**
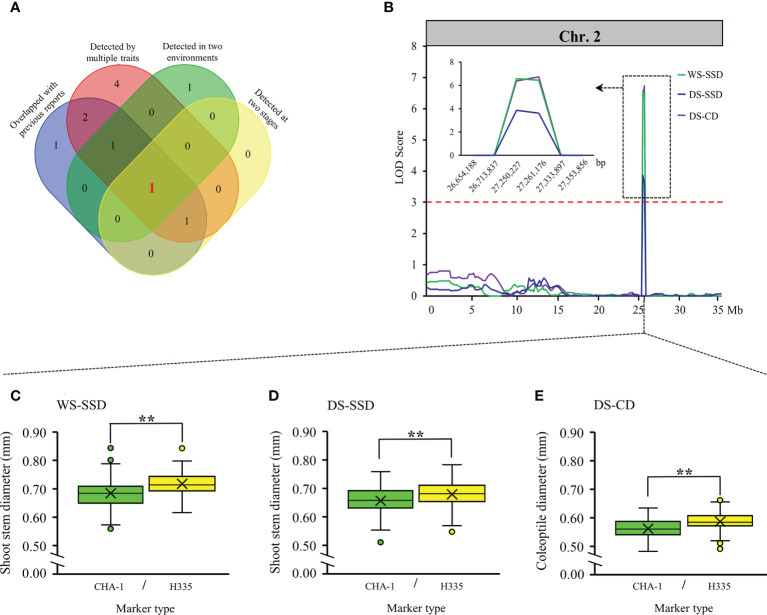
Validation of reliable loci. **(A)** Venn diagrams showing unique and shared criteria for QTLs detected in our study. **(B)** locus 3 located on chromosome 2 identified by linkage analysis. The box shows a magnification of the QTL candidate interval region on chromosome 2. **(C-E)** Boxplots showing the distribution of phenotypic differences between the two alleles of locus 3. Differences between the genotypes were analyzed using Student’s t-test, ** represents significance at the 0.01 level.

Among 20 loci we detected, locus 3 was implicated by multiple traits in both environments. In addition, locus 3 affects traits at both the germination and bud stages (**Figure 4A**). Notably, although it overlapped with previous reports (Jiang et al., 2004; Wang, 2009; Septiningsih et al., 2013), but no candidate genes were identified. To confirm the contributions of locus 3, present study summarize the phenotypic differences among two alleles of every QTL, and results illustrated that the phenotypic variations between two alleles were extremely significant within population (*p* ≤ 0.01) (**Figures 4C-E**). Therefore, we believe that locus 3 is very reliable and is valuable for further research and utilization.

Within the locus 3 interval (26,713,837 to 27,333,897 bp) on chromosome 2 (**Figure 4B**), 103 genes (**Table S5**) were identified from MSU Rice Genome Annotation Project database of version 7. To reduce number of candidate genes, we take benefit of three earlier expression profiles reported that were obtained from cultivars with respect to the aerobic and an anoxic germination (Lasanthi-Kudahettige et al., 2007; Narsai et al., 2009; Hsu and Tung, 2017). Based on gene differential expression multiples and the gene annotation, we focused on four candidate genes: *expansin precursor* (LOC_Os02g44108), *trehalose-6-phosphate phosphatase* (*OsTPP1*, LOC_Os02g44230), and two *LTPL113-Protease inhibitor/seed storage/LTP family protein precursor* (LOC_Os02g44310 and LOC_Os02g44320).

**Figure 5:**
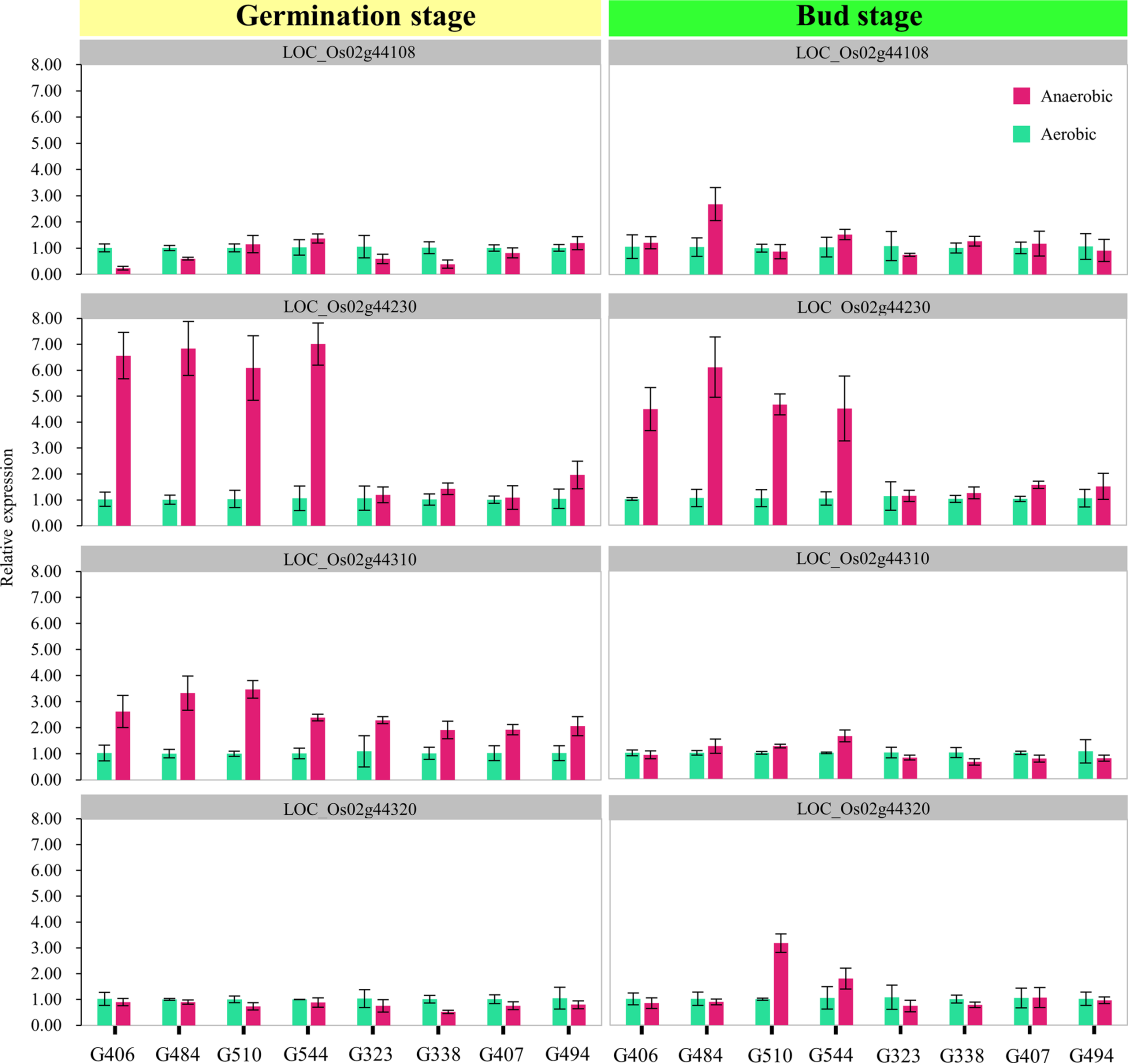
Expression analysis of candidate genes. The expression levels of four candidate genes in tissues (seeds + seedlings) of treated four-day-old seeds at the germination stage and bud stage respectively were measured using quantitative RT-PCR. The x-axis lists the eight genotypes whose phenotypes contrast (tolerant lines carrying H335 marker type alleles: G406, G484, G510, and G544; sensitive lines carrying CHA-1 marker type alleles: G323, G338, G407, and G494). The magenta and dark green boxes represent the control and submerged samples, respectively. The error bars correspond to the standard deviations (n = 3).

To examine the expression patterns of these four candidate genes in our materials, we chose eight RILs with contrasting phenotypic performances on SSD and CD (tolerant lines carrying H335 marker type alleles: G406, G484, G510, and G544; sensitive lines carrying CHA-1 marker type alleles: G323, G338, G407, and G494). The qRT-PCR was carried out using total RNA isolated from the tissues (seeds + seedlings) of treated four-day-old seeds at the germination stage and bud stage, respectively (**Figure 5**). Notably, the expression level of LOC_Os02g44230 (*OsTPP1*) was sharply upregulated in submerged plants for four tolerant RILs at both the germination and bud stage. However, the expression level of *OsTPP1* was almost unchanged in the four sensitive RILs and was not induced by anaerobic conditions.

## Discussion

### Using a RIL population to develop a high-density bin map could enhance the efficiency of genetic analyses of quantitative traits in rice

Mapping populations can be divided into temporary populations and permanent populations depending on the homozygosity. Temporary populations, for example, F_2_ population, is highly genetically diverse, but they cannot produce repeated observations. In addition, due to the complexity of the genetic background, the positioning of the QTL effect and location accuracy were low, which was suitable for the positioning of only major genes. Permanent populations, for example, RIL population, can produce repeated observations and can reduce the complexity of the genetic background. The location and effects of QTLs can be well estimated (Yin, 2015). The ability of rice seeds to develop into anaerobic seedlings is a quantitative trait. Therefore, a RIL population is an ideal mapping population. In our study, we constructed a RIL population, and this population will help us to identify more genetic loci effectively.

QTL mapping resolution depends on both density of marker and mapping population size (Da et al., 2000). Usually, the markers increasing density is an efficient approach to enhance the QTL mapping resolution (Liu et al., 2008). High-throughput resequencing strategies are currently being used to map QTLs accurately. In this experiment, map of high density for RIL population was constructed *via* the GBS strategy and was utilized for the QTL mapping in rice. Mean physical distance among two markers was 149.38 kb, and the smallest physical QTL interval was approximately 50 kb (**Table 3**). Compared with that in previous QTL mapping results concerning AG ability through restriction fragment length polymorphism (RFLP) or by the simple sequence repeat (SSR) markers, this distance has been narrowed significantly (Jiang et al., 2006; Angaji, 2008; Angaji et al., 2010; Septiningsih et al., 2013; Manangkil et al., 2013). The results confirmed that, compared with the use of traditional markers, GBS has significantly increased the QTL mapping resolution.

### Additional indicators should be developed for the estimation of anaerobic seedling establishment

Few studies have evaluated genetic loci known to be associated with anaerobic seedling establishment. One of the main reasons for this is that researchers have focused only on two traits directly related to anaerobic seedling establishment. The first trait is the rate of seedling survival after twenty one days submerged under the 10 or 20 cm of water. This was a technique developed by the International Institute of Rice Research (Angaji et al., 2010). The second trait is coleoptile elongation under anaerobic conditions. In previous studies on QTL mapping with these two indicators, some of the genetic loci identified overlapped with each other (Hsu and Tung, 2015; Zhang et al., 2017). The results indicate that these two indicators are reasonable. In present research, traits of coleoptile were validated by QTL mapping (**Table 3**). While, two loci were detected for CL, 18 loci were detected for CV, CSA, and CD. Notably, some genetic loci obtained using CSA, CV, CD, SSD and SFW overlapped with the previously reported using survival rate (**Table 3**). These results indicate that our indicators are feasible and that more high-efficiency indicators are necessary.

The ability of rice seeds to develop into anaerobic seedlings is a complex trait. It is well known that, in the process of anaerobic seedling establishment, both survival rate and the quality of the surviving seedlings are traits that should receive attention. However, neither survival rate nor coleoptile elongation are indicators that have directly and effectively been used to evaluate the quality of the surviving seedlings. Notably, seeds whose shoot length was approximately 3 mm were treated with anaerobic conditions, can successfully grew roots and leaves. In addition, many farmers prefer pregermination before sowing, indicating that the ability to survive and grow under anaerobic conditions at the bud stage is also critical. Therefore, seeds whose shoots were approximately 3 mm in length were treated with anaerobic conditions, which can help us evaluate relatively more phenotypes and represents conditions that are close to those of actual production.

### Different genetic mechanisms control anaerobic tolerance at germination stage and bud stage in the rice

In previous studies, we did not observe the emergence of genetic loci in mapping studies associated to anaerobic tolerance at bud stage. In our preliminary experiment, seeds at the pigeon breast stage or those whose shoot length was approximately 1 mm were treated with anaerobic treatment, just as the seeds without pre germination were treated directly. The final length of the rice coleoptiles under anaerobic conditions exceeded that of the rice coleoptiles under aerobic conditions, while the roots and primary leaves failed to grow. However, when rice seeds whose shoot length was approximately 3 mm were selected for anaerobic treatment, we observed the growth of roots and leaves. Of course, compared with that under aerobic conditions, the growth of buds and roots under anaerobic conditions was inhibited. In addition, among the 20 loci we detected, only locus 3 and 4 were detected at germination and bud stages. Present findings revealed that the genetic mechanisms of anaerobic tolerance at germination and bud stage are different. With respect to anaerobic tolerance, locus 3 and 4 were identified at various stages, revealing that there may be an overlap between metabolic pathways of anaerobic tolerance at various stages of rice.

### Considerable evidence suggests that *OsTPP1* might involved in mediating the variations in anaerobic seedling establishment ability

Kretzschmar et al. (2015) find T6P phosphatase gene, *OsTPP7*, fine mapping within *qAG-9-2* (Angaji et al., 2010), a key QTL for AG tolerance. *OsTPP7* is involved in metabolism of T6P, which is central to the energy sensor which determine catabolism or anabolism depending on the availability of local sucrose (Paul, 2008; Zhang et al., 2009). By representing low availability of sugar *via* enhanced T6P turnover, *OsTPP7* activity might increased the sink strength in the proliferating heterotrophic tissues, thus increasing starch mobilization to drive the kinetics of growth in germinating embryo and elongating coleoptile, which as a result enhances AG tolerance (Kretzschmar et al., 2015).

In the yeast and microbes, trehalose produced from glucose by the enzymes i.e., trehalose-6-phosphate synthase (TPS) and trehalose-6-phosphate phosphatase (TPP) works as metabolic regulator, sugar storage molecule and offers protection against abiotic stress (Wiemken, 1990; Strom and Kaasen, 1993). Thirteen *TREHALOSE-6-PHOSPHATE PHOSPHATASE* (*TPP*)-like genes are present in the rice (Ge et al., 2008), of which the products of *OsTPP1* and *OsTPP2* have reported to convert T6P to trehalose (Shima et al., 2007). In addition, *OsTPP1* over expression in the rice enhances tolerance to cold and salt stress (Ge et al., 2008). In particular, a recent study showed that *OsTPP1* regulates seed germination by crosstalk with abscisic acid in the rice (Wang et al., 2021). In our study, *OsTPP1* was derived from a genetic site that had a relatively high contribution rate and reliability. Expression analysis demonstrated that expression of *OsTPP1* was significantly induced by the anaerobic environment during both the germination and bud stages. Based on this evidence, we speculate that *OsTPP1* may be one of a main factors influencing the phenotypic changes in our RIL population. This might be engaged in mediating the variations in anaerobic seedling establishment.

## Conclusion

In our study, using the GBS approach, we developed a RIL population along with the construction of a map of genetic linkage having an average of 0.95 cM genetic distance among adjacent markers. Genetic mapping of quantitative traits was performed for 12 traits related to anaerobic seedling establishment at germination as well as bud stage during both the cropping seasons, a total of 20 loci were obtained. Among them, locus 3 was highly reliable and valuable for fine mapping. Within the interval, by combining gene annotation and expression analysis data, we found a promising candidate gene, *OsTPP1*. Present findings are useful for increasing our understanding of the genetic mechanism of anaerobic seedling establishment.

## Data availability statement

The data presented in the study are deposited in the Sequence Read Archive database (www.ncbi.nlm.nih.gov/sra) at NCBI (National Center for Biotechnology Information), accession number PRJNA857157. The names of the repository/repositories and accession number(s) can be found in the article/**Supplementary Material**.

## Author contributions

HW, SF and JL designed the project, and JY performed all the experiments and wrote the manuscript. JW, JX, YX and GD assisted in conducting the experiments and analyzing the data. HW, SF and JY provided the direction for the study and the corrections of the manuscript. All authors contributed to the article and approved the submitted version.

## Funding

This research was funded by the grant from the Yunnan Fundamental Research Project (Grant No. 202201AU070059, 202201AT070034, 202201AT070150) and the scientific research fund project of Yunnan Provincial Department of Education (Grant No. 2022J0134). The funding agency had no input into experimental design, the conduct of the research or the analysis, interpretation of experimental results and in writing the manuscript.

## Conflict of interest

The authors declare that the research was conducted in the absence of any commercial or financial relationships that could be construed as a potential conflict of interest.

## Publisher’s note

All claims expressed in this article are solely those of the authors and do not necessarily represent those of their affiliated organizations, or those of the publisher, the editors and the reviewers. Any product that may be evaluated in this article, or claim that may be made by its manufacturer, is not guaranteed or endorsed by the publisher.

## Abbreviations

RIL, recombinant inbred line; CL, coleoptile length; CSA, coleoptile surface area; CD, coleoptile diameter; CV, coleoptile volume; SSD, shoot stem diameter; SH, shoot height; SFW, shoot fresh weight; RL, root length; RSA, root surface area; RV, root volume; RD, root diameter; RFW, root fresh weight; AG, anaerobic germination; QTL, quantitative trait loci; GWAS, genome-wide sequencing analysis; DSR, direct seeding rice; WS, wet season; DS, wet season; GBS, genotyping-by-sequencing; LOD, Logarithm of odds; PVE, Phenotypic variation explained.
