# Supplementary material for: Mapping QTLs for anaerobic tolerance at germination and bud stages using new high density genetic map of rice
Source: Front Plant Sci. 2022 Oct 17;13:985080. doi: 10.3389/fpls.2022.985080 (PMC9618957; doi:10.3389/fpls.2022.985080)
Supplement: Supplementary file 4 [file Table_1.docx]

| **Supplementary Table S1** Primers used for qRT-PCR. | |  |  |
| --- | --- | --- | --- |
| Gene ID | Forward Sequence | Reverse Sequence | Product Size (bp) |
| LOC_Os02g44108 | GCACCTTGGTACTACTACTTGT | CTACCGGTGTAGTTGTTGTGAG | 151 |
| LOC_Os02g44230 | GAGCTTTATTATGCTGGAAGCC | CTCATCGATCATTGGCAGAAAG | 132 |
| LOC_Os02g44310 | GATTAGTTGGCTTTGACGACTC | AATCCAAACTGGTACCGTGATA | 148 |
| LOC_Os02g44320 | CTCTCTCTAGTTGCAGACCATC | GTGAAGAAGAGGACCAGGTTTA | 134 |
| *Acting* | CCCTCCTGAAAGGAAGTACAGTGT | GTCCGAAGAATTAGAAGCATTTCC | 146 |
