# Supplementary material for: Mapping QTLs for anaerobic tolerance at germination and bud stages using new high density genetic map of rice
Source: Front Plant Sci. 2022 Oct 17;13:985080. doi: 10.3389/fpls.2022.985080 (PMC9618957; doi:10.3389/fpls.2022.985080)
Supplement: Supplementary file 5 [file Table_2.docx]

| **Supplementary Table S2** Basic quality control of sequencing data. | | | | | | |  |  |  |  |  |  |  |
| --- | --- | --- | --- | --- | --- | --- | --- | --- | --- | --- | --- | --- | --- |
| **Sample** | **Raw Base (bp)** | **Clean Base (bp)** | **Effective Rate (%)** | **Error Rate (%)** | **Q20 (%)** | **Q30 (%)** | **GC Content (%)** | **Clean reads** | **Mapped reads** | **Mapping rate (%)** | **Average depth (X)** | **Coverage at least 1X (%)** | **Coverage at least 4X (%)** |
| G195 | 461588544 | 461582784 | 100.00 | 0.03 | 95.88 | 90.10 | 40.41 | 3205436 | 3102440 | 96.79 | 15.81 | 12.20 | 6.79 |
| G196 | 848033568 | 847966752 | 99.99 | 0.03 | 96.06 | 90.43 | 39.41 | 5888658 | 5711036 | 96.98 | 24.11 | 17.00 | 8.18 |
| G197 | 355107456 | 355078656 | 99.99 | 0.03 | 96.19 | 90.72 | 40.02 | 2465824 | 2392460 | 97.02 | 12.63 | 12.80 | 6.20 |
| G198 | 348488640 | 348459840 | 99.99 | 0.03 | 96.09 | 90.50 | 40.37 | 2419860 | 2349702 | 97.10 | 12.90 | 11.84 | 6.07 |
| G199 | 386483040 | 386447040 | 99.99 | 0.03 | 96.20 | 90.80 | 39.69 | 2683660 | 2603810 | 97.02 | 14.22 | 12.47 | 6.10 |
| G200 | 386263584 | 386227872 | 99.99 | 0.03 | 96.34 | 91.10 | 39.52 | 2682138 | 2607093 | 97.20 | 11.88 | 16.24 | 6.76 |
| G201 | 396147168 | 396114336 | 99.99 | 0.03 | 96.19 | 90.71 | 40.44 | 2750794 | 2663149 | 96.81 | 14.46 | 11.28 | 6.35 |
| G202 | 393097536 | 393055776 | 99.99 | 0.03 | 95.75 | 89.66 | 40.19 | 2729554 | 2649757 | 97.08 | 13.22 | 14.00 | 6.51 |
| G203 | 434945376 | 434908224 | 99.99 | 0.03 | 96.28 | 91.04 | 40.12 | 3020196 | 2928935 | 96.98 | 13.23 | 14.09 | 7.43 |
| G204 | 412525728 | 412487424 | 99.99 | 0.03 | 96.03 | 90.36 | 40.22 | 2864496 | 2727077 | 95.20 | 13.39 | 13.45 | 6.77 |
| G205 | 467299008 | 467257536 | 99.99 | 0.03 | 96.26 | 90.89 | 40.36 | 3244844 | 3146295 | 96.96 | 15.07 | 13.19 | 7.16 |
| G206 | 445226112 | 445185504 | 99.99 | 0.03 | 96.23 | 90.86 | 40.44 | 3091566 | 2994500 | 96.86 | 14.62 | 12.82 | 7.02 |
| G207 | 472439808 | 472405248 | 99.99 | 0.03 | 96.09 | 90.41 | 39.88 | 3280592 | 3182585 | 97.01 | 16.62 | 12.62 | 6.57 |
| G208 | 341147808 | 341120736 | 99.99 | 0.03 | 95.84 | 89.91 | 40.12 | 2368894 | 2284103 | 96.42 | 12.11 | 12.90 | 6.09 |
| G209 | 358625088 | 358591392 | 99.99 | 0.03 | 96.07 | 90.40 | 40.49 | 2490218 | 2416943 | 97.06 | 12.20 | 12.84 | 6.55 |
| G210 | 325963296 | 325930752 | 99.99 | 0.03 | 95.91 | 90.02 | 40.56 | 2263408 | 2191804 | 96.84 | 12.28 | 11.61 | 5.90 |
| G211 | 345779136 | 345750048 | 99.99 | 0.03 | 96.21 | 90.73 | 40.44 | 2401042 | 2325567 | 96.86 | 12.37 | 12.12 | 6.24 |
| G212 | 397689408 | 397653408 | 99.99 | 0.03 | 96.22 | 90.96 | 40.21 | 2761482 | 2676899 | 96.94 | 12.40 | 13.83 | 7.15 |
| G213 | 393997824 | 393964128 | 99.99 | 0.03 | 96.21 | 90.82 | 40.26 | 2735862 | 2652408 | 96.95 | 13.85 | 12.18 | 6.49 |
| G214 | 410154336 | 410113440 | 99.99 | 0.03 | 96.20 | 90.81 | 40.07 | 2848010 | 2759761 | 96.90 | 12.72 | 14.62 | 7.06 |
| G215 | 412060032 | 412028064 | 99.99 | 0.03 | 96.14 | 90.81 | 39.96 | 2861306 | 2772567 | 96.90 | 12.63 | 14.61 | 7.17 |
| G216 | 423061920 | 423021600 | 99.99 | 0.03 | 96.34 | 91.09 | 40.38 | 2937650 | 2852602 | 97.10 | 13.36 | 13.53 | 7.19 |
| G217 | 407856384 | 407816928 | 99.99 | 0.03 | 95.77 | 89.69 | 40.28 | 2832062 | 2743591 | 96.88 | 13.55 | 13.63 | 6.69 |
| G218 | 408550752 | 408518496 | 99.99 | 0.03 | 95.98 | 90.23 | 40.52 | 2836934 | 2753386 | 97.05 | 14.66 | 11.90 | 6.43 |
| G219 | 449003520 | 448971264 | 99.99 | 0.03 | 96.34 | 91.09 | 40.41 | 3117856 | 3004427 | 96.36 | 14.56 | 13.12 | 7.03 |
| G220 | 367350048 | 367319520 | 99.99 | 0.03 | 95.99 | 90.16 | 40.56 | 2550830 | 2475330 | 97.04 | 13.26 | 11.98 | 6.27 |
| G221 | 392518656 | 392486688 | 99.99 | 0.03 | 96.31 | 91.11 | 40.17 | 2725602 | 2641457 | 96.91 | 12.66 | 13.33 | 6.95 |
| G222 | 426580416 | 426541824 | 99.99 | 0.03 | 96.19 | 90.88 | 40.24 | 2962096 | 2872004 | 96.96 | 14.63 | 12.08 | 6.77 |
| G223 | 404440992 | 404407296 | 99.99 | 0.03 | 96.22 | 90.80 | 40.38 | 2808384 | 2723043 | 96.96 | 12.97 | 13.55 | 7.00 |
| G224 | 386208576 | 386176320 | 99.99 | 0.03 | 96.30 | 91.03 | 39.96 | 2681780 | 2592359 | 96.67 | 12.65 | 14.26 | 6.59 |
| G225 | 416492064 | 416459808 | 99.99 | 0.03 | 95.94 | 90.14 | 40.42 | 2892082 | 2802446 | 96.90 | 13.07 | 14.36 | 7.05 |
| G226 | 369218880 | 369187488 | 99.99 | 0.03 | 95.76 | 89.62 | 40.14 | 2563802 | 2466167 | 96.19 | 12.17 | 13.99 | 6.49 |
| G227 | 431753184 | 431714016 | 99.99 | 0.03 | 96.14 | 90.59 | 40.04 | 2998014 | 2912766 | 97.16 | 14.82 | 13.14 | 6.62 |
| G228 | 409818240 | 409786272 | 99.99 | 0.03 | 96.07 | 90.41 | 40.30 | 2845738 | 2764710 | 97.15 | 13.63 | 13.55 | 6.75 |
| G229 | 385214112 | 385182720 | 99.99 | 0.03 | 94.97 | 87.81 | 40.44 | 2674880 | 2597896 | 97.12 | 14.93 | 10.99 | 5.97 |
| G230 | 434800224 | 434764800 | 99.99 | 0.03 | 96.14 | 90.65 | 40.24 | 3019200 | 2929603 | 97.03 | 14.91 | 12.68 | 6.70 |
| G231 | 469211904 | 469171872 | 99.99 | 0.03 | 96.06 | 90.47 | 40.38 | 3258138 | 3157425 | 96.91 | 15.64 | 12.85 | 6.93 |
| G232 | 388885536 | 388876896 | 100.00 | 0.03 | 95.88 | 89.92 | 39.58 | 2700534 | 2615294 | 96.84 | 14.62 | 12.41 | 5.95 |
| G233 | 375119136 | 375108192 | 100.00 | 0.03 | 96.00 | 90.20 | 40.17 | 2604918 | 2524464 | 96.91 | 13.01 | 13.17 | 6.37 |
| G234 | 371189088 | 371179296 | 100.00 | 0.03 | 95.94 | 90.02 | 40.26 | 2577634 | 2503174 | 97.11 | 13.36 | 12.09 | 6.30 |
| G235 | 388284480 | 388275552 | 100.00 | 0.03 | 96.09 | 90.42 | 39.77 | 2696358 | 2619875 | 97.16 | 14.59 | 12.02 | 6.05 |
| G236 | 360719712 | 360712800 | 100.00 | 0.03 | 96.19 | 90.66 | 39.74 | 2504950 | 2431837 | 97.08 | 12.27 | 14.54 | 6.20 |
| G237 | 378508032 | 378498816 | 100.00 | 0.03 | 96.06 | 90.31 | 40.54 | 2628464 | 2555706 | 97.23 | 12.37 | 13.22 | 6.87 |
| G238 | 322504128 | 322498080 | 100.00 | 0.03 | 95.46 | 88.91 | 39.89 | 2239570 | 2154376 | 96.20 | 11.00 | 14.52 | 5.90 |
| G239 | 376784640 | 376771968 | 100.00 | 0.03 | 96.14 | 90.62 | 40.37 | 2616472 | 2533661 | 96.84 | 12.89 | 12.56 | 6.58 |
| G240 | 395468928 | 395459424 | 100.00 | 0.03 | 95.75 | 89.66 | 40.27 | 2746246 | 2640892 | 96.16 | 13.80 | 12.68 | 6.41 |
| G241 | 411712992 | 411702624 | 100.00 | 0.03 | 96.10 | 90.44 | 40.36 | 2859046 | 2770818 | 96.91 | 13.66 | 13.17 | 6.81 |
| G242 | 416887488 | 416874528 | 100.00 | 0.03 | 96.07 | 90.39 | 40.50 | 2894962 | 2806573 | 96.95 | 14.06 | 12.51 | 6.81 |
| G243 | 422410464 | 422403264 | 100.00 | 0.03 | 95.86 | 89.77 | 39.88 | 2933356 | 2853293 | 97.27 | 15.41 | 12.26 | 6.31 |
| G244 | 389204352 | 389198016 | 100.00 | 0.03 | 95.57 | 89.20 | 40.04 | 2702764 | 2616983 | 96.83 | 13.74 | 12.56 | 6.38 |
| G245 | 344307168 | 344297376 | 100.00 | 0.03 | 95.77 | 89.59 | 40.42 | 2390954 | 2321968 | 97.11 | 12.33 | 12.30 | 6.24 |
| G246 | 313359264 | 313352352 | 100.00 | 0.03 | 95.63 | 89.28 | 40.39 | 2176058 | 2110662 | 96.99 | 11.54 | 12.30 | 5.91 |
| G247 | 355574880 | 355566816 | 100.00 | 0.03 | 95.93 | 90.02 | 40.50 | 2469214 | 2393877 | 96.95 | 13.40 | 11.27 | 6.06 |
| G248 | 377819712 | 377810208 | 100.00 | 0.03 | 96.12 | 90.61 | 40.32 | 2623682 | 2549679 | 97.18 | 13.05 | 12.57 | 6.55 |
| G249 | 415177056 | 415164672 | 100.00 | 0.03 | 96.10 | 90.44 | 40.17 | 2883088 | 2801963 | 97.19 | 13.55 | 13.74 | 6.87 |
| G250 | 416052864 | 416042784 | 100.00 | 0.03 | 96.00 | 90.25 | 40.16 | 2889186 | 2804487 | 97.07 | 14.24 | 12.97 | 6.63 |
| G251 | 433669536 | 433659456 | 100.00 | 0.03 | 96.00 | 90.38 | 40.22 | 3011524 | 2891117 | 96.00 | 14.01 | 13.45 | 6.94 |
| G252 | 373162176 | 373156416 | 100.00 | 0.03 | 96.16 | 90.59 | 40.48 | 2591364 | 2512508 | 96.96 | 12.93 | 12.39 | 6.52 |
| G253 | 370621152 | 370612512 | 100.00 | 0.03 | 95.38 | 88.74 | 40.22 | 2573698 | 2494187 | 96.91 | 13.24 | 12.29 | 6.30 |
| G254 | 366686496 | 366677856 | 100.00 | 0.03 | 95.77 | 89.63 | 40.41 | 2546374 | 2471041 | 97.04 | 13.88 | 10.98 | 6.11 |
| G255 | 390902688 | 390890304 | 100.00 | 0.03 | 96.18 | 90.58 | 40.44 | 2714516 | 2628724 | 96.84 | 14.09 | 11.47 | 6.40 |
| G256 | 364277664 | 364271328 | 100.00 | 0.03 | 96.38 | 90.91 | 39.27 | 2529662 | 2436509 | 96.32 | 14.24 | 12.52 | 5.61 |
| G257 | 357526944 | 357519168 | 100.00 | 0.03 | 96.48 | 91.18 | 39.71 | 2482772 | 2318288 | 93.37 | 12.49 | 13.18 | 5.98 |
| G258 | 338526720 | 338521248 | 100.00 | 0.03 | 96.36 | 90.87 | 40.19 | 2350842 | 2275337 | 96.79 | 12.59 | 12.26 | 5.96 |
| G259 | 340393536 | 340387200 | 100.00 | 0.03 | 96.54 | 91.34 | 39.38 | 2363800 | 2298127 | 97.22 | 12.97 | 12.83 | 5.72 |
| G260 | 368572896 | 368565696 | 100.00 | 0.03 | 96.65 | 91.58 | 39.47 | 2559484 | 2478798 | 96.85 | 12.31 | 14.90 | 6.29 |
| G261 | 340895520 | 340890336 | 100.00 | 0.03 | 96.56 | 91.32 | 40.28 | 2367294 | 2289343 | 96.71 | 11.80 | 13.11 | 6.32 |
| G262 | 313327872 | 313324416 | 100.00 | 0.03 | 96.02 | 90.00 | 39.98 | 2175864 | 2109699 | 96.96 | 10.99 | 14.12 | 5.87 |
| G263 | 343865088 | 343854144 | 100.00 | 0.03 | 96.53 | 91.44 | 39.98 | 2387876 | 2316381 | 97.01 | 11.89 | 13.28 | 6.33 |
| G264 | 353365344 | 353359872 | 100.00 | 0.03 | 96.16 | 90.45 | 39.95 | 2453888 | 2377606 | 96.89 | 12.73 | 13.10 | 6.08 |
| G265 | 381051072 | 381042432 | 100.00 | 0.03 | 96.52 | 91.29 | 40.13 | 2646128 | 2562644 | 96.85 | 12.78 | 13.39 | 6.62 |
| G266 | 357508224 | 357499296 | 100.00 | 0.03 | 96.62 | 91.52 | 40.27 | 2482634 | 2407803 | 96.99 | 12.47 | 12.91 | 6.35 |
| G267 | 405992736 | 405984960 | 100.00 | 0.03 | 96.43 | 90.92 | 39.73 | 2819340 | 2710184 | 96.13 | 14.98 | 12.54 | 6.09 |
| G268 | 440663616 | 440659584 | 100.00 | 0.03 | 96.13 | 90.24 | 39.94 | 3060136 | 2967997 | 96.99 | 14.45 | 14.29 | 6.86 |
| G269 | 242105184 | 242102304 | 100.00 | 0.03 | 96.32 | 90.67 | 40.29 | 1681266 | 1626509 | 96.74 | 10.22 | 11.19 | 4.96 |
| G270 | 339515712 | 339511968 | 100.00 | 0.03 | 96.13 | 90.27 | 40.35 | 2357722 | 2285796 | 96.95 | 11.78 | 13.41 | 6.23 |
| G271 | 377894304 | 377887392 | 100.00 | 0.03 | 96.45 | 91.07 | 40.19 | 2624218 | 2522919 | 96.14 | 12.43 | 13.73 | 6.63 |
| G272 | 392976000 | 392964192 | 100.00 | 0.03 | 96.53 | 91.43 | 40.07 | 2728918 | 2650304 | 97.12 | 12.49 | 14.20 | 7.00 |
| G273 | 354026880 | 354020544 | 100.00 | 0.03 | 96.53 | 91.34 | 39.91 | 2458476 | 2380124 | 96.81 | 12.18 | 13.66 | 6.29 |
| G274 | 393574464 | 393565536 | 100.00 | 0.03 | 96.44 | 91.12 | 40.02 | 2733094 | 2650464 | 96.98 | 13.34 | 13.69 | 6.53 |
| G275 | 413068320 | 413056224 | 100.00 | 0.03 | 96.47 | 91.35 | 39.88 | 2868446 | 2770141 | 96.57 | 12.72 | 15.01 | 7.06 |
| G276 | 359572320 | 359564544 | 100.00 | 0.03 | 96.61 | 91.51 | 40.23 | 2496976 | 2415727 | 96.75 | 12.35 | 13.23 | 6.42 |
| G277 | 356665824 | 356663232 | 100.00 | 0.03 | 96.06 | 90.03 | 40.17 | 2476828 | 2404829 | 97.09 | 12.34 | 13.45 | 6.35 |
| G278 | 330554304 | 330549984 | 100.00 | 0.03 | 96.23 | 90.54 | 40.33 | 2295486 | 2220341 | 96.73 | 12.87 | 11.50 | 5.75 |
| G279 | 376161696 | 376156800 | 100.00 | 0.03 | 96.64 | 91.52 | 40.28 | 2612200 | 2508399 | 96.03 | 13.08 | 12.79 | 6.39 |
| G280 | 360824544 | 360820800 | 100.00 | 0.03 | 96.27 | 90.50 | 40.41 | 2505700 | 2430568 | 97.00 | 13.26 | 12.20 | 6.12 |
| G281 | 420920352 | 420908544 | 100.00 | 0.03 | 96.51 | 91.41 | 40.08 | 2922976 | 2832649 | 96.91 | 13.75 | 13.78 | 6.88 |
| G282 | 393512832 | 393499584 | 100.00 | 0.03 | 96.54 | 91.50 | 39.91 | 2732636 | 2655018 | 97.16 | 13.37 | 13.21 | 6.65 |
| G283 | 377338752 | 377329248 | 100.00 | 0.03 | 96.50 | 91.22 | 40.23 | 2620342 | 2540947 | 96.97 | 12.28 | 13.91 | 6.74 |
| G284 | 386418240 | 386412768 | 100.00 | 0.03 | 96.53 | 91.33 | 39.92 | 2683422 | 2602865 | 97.00 | 13.13 | 13.98 | 6.43 |
| G285 | 355468032 | 355465152 | 100.00 | 0.03 | 96.22 | 90.48 | 40.33 | 2468508 | 2394982 | 97.02 | 12.77 | 12.71 | 6.19 |
| G286 | 349240320 | 349236288 | 100.00 | 0.03 | 95.94 | 89.72 | 40.09 | 2425252 | 2354146 | 97.07 | 12.91 | 12.61 | 5.99 |
| G287 | 373797504 | 373791744 | 100.00 | 0.03 | 96.47 | 91.11 | 39.99 | 2595776 | 2505799 | 96.53 | 13.77 | 12.50 | 6.04 |
| G288 | 352993248 | 352990080 | 100.00 | 0.03 | 96.37 | 90.75 | 40.38 | 2451320 | 2351223 | 95.92 | 13.00 | 12.33 | 5.97 |
| G289 | 284498496 | 284496768 | 100.00 | 0.04 | 95.04 | 87.56 | 40.58 | 1975672 | 1918654 | 97.11 | 12.15 | 10.82 | 5.16 |
| G290 | 348707808 | 348702048 | 100.00 | 0.03 | 96.41 | 91.03 | 40.05 | 2421542 | 2348157 | 96.97 | 13.21 | 12.14 | 5.89 |
| G291 | 374213664 | 374207904 | 100.00 | 0.03 | 96.40 | 90.98 | 40.32 | 2598666 | 2514747 | 96.77 | 13.98 | 11.86 | 6.07 |
| G292 | 421343424 | 421234560 | 99.97 | 0.02 | 96.98 | 91.82 | 40.33 | 2925240 | 2845059 | 97.26 | 15.95 | 11.68 | 6.13 |
| G293 | 488369088 | 488239200 | 99.97 | 0.02 | 97.16 | 92.31 | 40.75 | 3390550 | 3287999 | 96.98 | 17.44 | 12.25 | 6.48 |
| G294 | 411924960 | 411821568 | 99.97 | 0.02 | 97.00 | 91.93 | 40.54 | 2859872 | 2772513 | 96.95 | 15.46 | 11.77 | 6.13 |
| G295 | 353917440 | 353806848 | 99.97 | 0.02 | 97.25 | 92.62 | 40.65 | 2456992 | 2387081 | 97.15 | 13.88 | 11.08 | 5.81 |
| G296 | 344043072 | 343921536 | 99.96 | 0.02 | 97.29 | 92.88 | 40.53 | 2388344 | 2319219 | 97.11 | 13.39 | 11.07 | 5.83 |
| G297 | 336476160 | 336367872 | 99.97 | 0.02 | 97.27 | 92.72 | 40.27 | 2335888 | 2265992 | 97.01 | 12.69 | 12.15 | 5.83 |
| G298 | 370547712 | 370429056 | 99.97 | 0.02 | 97.20 | 92.59 | 40.45 | 2572424 | 2497300 | 97.08 | 14.69 | 10.86 | 5.80 |
| G299 | 379116576 | 378999936 | 99.97 | 0.02 | 97.29 | 92.88 | 40.34 | 2631944 | 2554830 | 97.07 | 13.89 | 12.37 | 6.11 |
| G300 | 391869216 | 391743072 | 99.97 | 0.02 | 97.34 | 92.91 | 40.69 | 2720438 | 2645326 | 97.24 | 14.66 | 11.54 | 6.12 |
| G301 | 359148096 | 359042976 | 99.97 | 0.03 | 96.87 | 91.59 | 40.56 | 2493354 | 2400174 | 96.26 | 14.58 | 10.74 | 5.60 |
| G302 | 349580736 | 349485696 | 99.97 | 0.02 | 96.97 | 91.97 | 40.91 | 2426984 | 2349500 | 96.81 | 14.67 | 10.05 | 5.47 |
| G303 | 366053184 | 365930208 | 99.97 | 0.02 | 97.38 | 92.97 | 40.75 | 2541182 | 2467196 | 97.09 | 13.98 | 11.25 | 5.97 |
| G304 | 392300352 | 392191200 | 99.97 | 0.03 | 96.94 | 91.78 | 40.70 | 2723550 | 2630988 | 96.60 | 15.36 | 10.89 | 5.87 |
| G305 | 400434912 | 400308768 | 99.97 | 0.02 | 97.32 | 92.96 | 40.44 | 2779922 | 2699956 | 97.12 | 14.94 | 11.64 | 6.16 |
| G306 | 323084448 | 322983072 | 99.97 | 0.02 | 97.40 | 93.12 | 40.41 | 2242938 | 2178606 | 97.13 | 12.88 | 10.89 | 5.64 |
| G307 | 378028800 | 377923104 | 99.97 | 0.02 | 97.25 | 92.67 | 40.64 | 2624466 | 2546929 | 97.05 | 13.88 | 11.88 | 6.15 |
| G308 | 375627168 | 375513984 | 99.97 | 0.02 | 97.26 | 92.72 | 40.31 | 2607736 | 2531119 | 97.06 | 14.21 | 11.86 | 5.94 |
| G309 | 334997280 | 334894464 | 99.97 | 0.02 | 97.00 | 92.05 | 40.71 | 2325656 | 2254259 | 96.93 | 13.56 | 10.66 | 5.59 |
| G310 | 329945184 | 329855328 | 99.97 | 0.03 | 96.67 | 91.14 | 40.45 | 2290662 | 2222294 | 97.02 | 13.02 | 11.39 | 5.62 |
| G311 | 382351392 | 382247424 | 99.97 | 0.02 | 97.28 | 92.65 | 40.16 | 2654496 | 2562265 | 96.53 | 14.77 | 11.57 | 5.86 |
| G312 | 391587552 | 391479264 | 99.97 | 0.02 | 97.03 | 92.06 | 40.64 | 2718606 | 2639408 | 97.09 | 15.24 | 11.14 | 5.92 |
| G313 | 349695360 | 349618176 | 99.98 | 0.03 | 95.91 | 89.33 | 40.87 | 2427904 | 2353270 | 96.93 | 14.84 | 10.08 | 5.41 |
| G314 | 389157120 | 389051424 | 99.97 | 0.02 | 97.23 | 92.62 | 40.56 | 2701746 | 2623599 | 97.11 | 14.95 | 11.23 | 5.98 |
| G315 | 821050560 | 820987200 | 99.99 | 0.03 | 96.55 | 94.53 | 39.50 | 5701300 | 5506748 | 96.59 | 27.94 | 11.34 | 7.14 |
| G316 | 636816960 | 636763104 | 99.99 | 0.03 | 96.47 | 94.42 | 40.17 | 4421966 | 4276415 | 96.71 | 20.80 | 11.85 | 7.35 |
| G317 | 530002656 | 529961184 | 99.99 | 0.03 | 96.14 | 93.89 | 40.31 | 3680286 | 3564048 | 96.84 | 18.43 | 11.17 | 6.87 |
| G318 | 503112384 | 503066016 | 99.99 | 0.03 | 96.83 | 94.97 | 39.89 | 3493514 | 3388854 | 97.00 | 18.20 | 10.78 | 6.61 |
| G319 | 529811136 | 529769952 | 99.99 | 0.03 | 96.78 | 94.90 | 40.08 | 3678958 | 3555803 | 96.65 | 16.00 | 13.07 | 7.74 |
| G320 | 406000512 | 405974016 | 99.99 | 0.03 | 96.54 | 94.53 | 40.25 | 2819264 | 2727703 | 96.75 | 15.48 | 10.35 | 6.19 |
| G321 | 557275680 | 557224416 | 99.99 | 0.03 | 95.76 | 93.31 | 40.50 | 3869614 | 3755540 | 97.05 | 17.10 | 12.91 | 7.68 |
| G322 | 610342560 | 610290720 | 99.99 | 0.02 | 97.19 | 95.50 | 40.11 | 4238130 | 4116658 | 97.13 | 16.58 | 14.51 | 8.76 |
| G323 | 514703520 | 514657728 | 99.99 | 0.03 | 96.61 | 94.63 | 40.24 | 3574012 | 3445692 | 96.41 | 16.88 | 12.03 | 7.18 |
| G324 | 546121440 | 546077088 | 99.99 | 0.03 | 96.94 | 95.16 | 40.29 | 3792202 | 3673664 | 96.87 | 16.94 | 12.73 | 7.60 |
| G325 | 576040032 | 575997408 | 99.99 | 0.03 | 96.88 | 95.04 | 40.35 | 3999982 | 3831185 | 95.78 | 16.31 | 13.57 | 8.21 |
| G326 | 443913696 | 443878272 | 99.99 | 0.03 | 96.30 | 94.14 | 39.78 | 3082488 | 2984202 | 96.81 | 16.50 | 10.92 | 6.32 |
| G327 | 510480000 | 510441696 | 99.99 | 0.03 | 96.20 | 94.01 | 40.26 | 3544734 | 3392126 | 95.69 | 17.78 | 11.37 | 6.71 |
| G328 | 437264640 | 437226912 | 99.99 | 0.03 | 96.16 | 93.92 | 40.52 | 3036298 | 2937183 | 96.74 | 14.90 | 11.86 | 6.84 |
| G329 | 428846688 | 428812992 | 99.99 | 0.03 | 96.02 | 93.72 | 40.45 | 2977868 | 2888163 | 96.99 | 14.76 | 11.73 | 6.77 |
| G330 | 431358336 | 431323776 | 99.99 | 0.03 | 96.56 | 94.57 | 40.49 | 2995304 | 2898828 | 96.78 | 13.63 | 12.86 | 7.27 |
| G331 | 397294272 | 397266624 | 99.99 | 0.03 | 96.97 | 95.19 | 40.23 | 2758796 | 2675186 | 96.97 | 14.56 | 10.88 | 6.38 |
| G332 | 370988928 | 370960128 | 99.99 | 0.03 | 96.61 | 94.65 | 40.32 | 2576112 | 2495459 | 96.87 | 14.51 | 10.08 | 5.99 |
| G333 | 408312864 | 408286368 | 99.99 | 0.03 | 96.55 | 94.55 | 40.26 | 2835322 | 2743520 | 96.76 | 14.31 | 11.48 | 6.62 |
| G334 | 390734208 | 390700512 | 99.99 | 0.02 | 97.12 | 95.42 | 40.27 | 2713198 | 2586562 | 95.33 | 14.02 | 11.09 | 6.38 |
| G335 | 384134976 | 384106176 | 99.99 | 0.03 | 96.84 | 94.97 | 40.33 | 2667404 | 2587507 | 97.00 | 13.47 | 11.54 | 6.59 |
| G336 | 421723296 | 421688448 | 99.99 | 0.03 | 95.74 | 93.30 | 40.42 | 2928392 | 2837505 | 96.90 | 14.69 | 11.50 | 6.69 |
| G337 | 358849728 | 358822656 | 99.99 | 0.03 | 95.89 | 93.53 | 40.50 | 2491824 | 2404063 | 96.48 | 13.55 | 10.64 | 6.09 |
| G338 | 411562944 | 411528672 | 99.99 | 0.03 | 96.50 | 94.45 | 40.32 | 2857838 | 2767663 | 96.84 | 14.09 | 11.71 | 6.75 |
| G339 | 425724768 | 425693952 | 99.99 | 0.03 | 95.47 | 92.86 | 40.39 | 2956208 | 2864768 | 96.91 | 14.42 | 11.97 | 6.86 |
| G340 | 364761792 | 364730688 | 99.99 | 0.02 | 97.20 | 95.55 | 40.17 | 2532852 | 2461790 | 97.19 | 13.65 | 10.70 | 6.21 |
| G341 | 354859200 | 354828672 | 99.99 | 0.02 | 97.29 | 95.66 | 40.09 | 2464088 | 2393657 | 97.14 | 13.28 | 10.72 | 6.21 |
| G342 | 386812224 | 386780544 | 99.99 | 0.03 | 96.41 | 94.31 | 40.33 | 2685976 | 2607803 | 97.09 | 14.18 | 10.99 | 6.36 |
| G343 | 386320320 | 386291808 | 99.99 | 0.03 | 96.72 | 94.81 | 40.27 | 2682582 | 2594752 | 96.73 | 14.55 | 10.45 | 6.19 |
| G344 | 315734112 | 315704448 | 99.99 | 0.03 | 96.00 | 93.69 | 40.35 | 2192392 | 2128674 | 97.09 | 13.64 | 9.10 | 5.43 |
| G345 | 425985696 | 425953152 | 99.99 | 0.03 | 95.69 | 93.22 | 40.39 | 2958008 | 2842625 | 96.10 | 14.39 | 11.65 | 6.82 |
| G346 | 415006848 | 414976608 | 99.99 | 0.03 | 96.53 | 94.49 | 39.94 | 2881782 | 2766885 | 96.01 | 15.64 | 10.45 | 6.21 |
| G347 | 378594720 | 378564768 | 99.99 | 0.03 | 95.94 | 93.62 | 40.14 | 2628922 | 2503566 | 95.23 | 15.07 | 9.46 | 5.85 |
| G348 | 399835584 | 399801600 | 99.99 | 0.03 | 94.31 | 90.98 | 40.60 | 2776400 | 2665605 | 96.01 | 14.68 | 10.80 | 6.28 |
| G349 | 376139232 | 376106976 | 99.99 | 0.03 | 96.63 | 94.67 | 40.09 | 2611854 | 2503012 | 95.83 | 14.68 | 9.96 | 5.95 |
| G350 | 410707584 | 410670432 | 99.99 | 0.03 | 96.42 | 94.34 | 40.17 | 2851878 | 2759686 | 96.77 | 14.63 | 11.10 | 6.60 |
| G351 | 379085472 | 379035936 | 99.99 | 0.03 | 95.91 | 93.66 | 39.23 | 2632194 | 2551722 | 96.94 | 13.76 | 11.59 | 6.32 |
| G352 | 390270528 | 390214080 | 99.99 | 0.03 | 95.79 | 93.48 | 39.77 | 2709820 | 2623546 | 96.82 | 14.38 | 11.05 | 6.31 |
| G353 | 367799904 | 367746912 | 99.99 | 0.03 | 95.37 | 92.83 | 39.90 | 2553798 | 2472198 | 96.80 | 13.86 | 10.89 | 6.14 |
| G354 | 389364480 | 389305728 | 99.98 | 0.03 | 96.24 | 94.16 | 39.50 | 2703512 | 2620006 | 96.91 | 14.59 | 10.90 | 6.23 |
| G355 | 375681024 | 375630624 | 99.99 | 0.03 | 96.16 | 94.07 | 39.54 | 2608546 | 2527286 | 96.88 | 13.11 | 12.09 | 6.52 |
| G356 | 335677248 | 335630592 | 99.99 | 0.03 | 95.78 | 93.47 | 39.97 | 2330768 | 2255147 | 96.76 | 13.05 | 10.69 | 5.89 |
| G357 | 350448768 | 350403264 | 99.99 | 0.03 | 94.68 | 91.75 | 40.06 | 2433356 | 2356131 | 96.83 | 13.08 | 11.14 | 6.13 |
| G358 | 356029344 | 355979808 | 99.99 | 0.03 | 96.82 | 95.07 | 39.86 | 2472082 | 2400425 | 97.10 | 12.69 | 11.77 | 6.39 |
| G359 | 346908960 | 346858560 | 99.99 | 0.03 | 96.02 | 93.84 | 39.84 | 2408740 | 2344176 | 97.32 | 12.82 | 11.23 | 6.23 |
| G360 | 346968576 | 346923648 | 99.99 | 0.03 | 96.48 | 94.56 | 40.00 | 2409192 | 2307362 | 95.77 | 12.91 | 11.03 | 6.08 |
| G361 | 341034336 | 340983360 | 99.99 | 0.03 | 96.28 | 94.24 | 39.92 | 2367940 | 2299467 | 97.11 | 12.54 | 11.49 | 6.17 |
| G362 | 313791840 | 313742304 | 99.98 | 0.03 | 95.45 | 92.94 | 39.55 | 2178766 | 2114565 | 97.05 | 13.53 | 9.39 | 5.40 |
| G363 | 302987808 | 302945760 | 99.99 | 0.03 | 95.37 | 92.80 | 39.80 | 2103790 | 2034532 | 96.71 | 12.54 | 10.15 | 5.48 |
| G364 | 291525696 | 291482784 | 99.99 | 0.03 | 95.13 | 92.44 | 40.11 | 2024186 | 1963846 | 97.02 | 11.81 | 10.58 | 5.53 |
| G365 | 325911744 | 325857888 | 99.98 | 0.03 | 95.07 | 92.35 | 40.03 | 2262902 | 2191451 | 96.84 | 11.13 | 12.46 | 6.48 |
| G366 | 339562944 | 339511104 | 99.98 | 0.03 | 95.88 | 93.63 | 40.00 | 2357716 | 2230248 | 94.59 | 12.39 | 11.23 | 6.06 |
| G367 | 314679744 | 314634528 | 99.99 | 0.03 | 96.52 | 94.60 | 39.88 | 2184962 | 2114028 | 96.75 | 12.38 | 10.58 | 5.77 |
| G368 | 312045696 | 312001632 | 99.99 | 0.03 | 95.98 | 93.80 | 39.92 | 2166678 | 2101697 | 97.00 | 12.40 | 10.46 | 5.73 |
| G369 | 308351232 | 308307168 | 99.99 | 0.03 | 95.88 | 93.61 | 39.96 | 2141022 | 2074736 | 96.90 | 11.71 | 11.21 | 5.88 |
| G370 | 340576128 | 340528032 | 99.99 | 0.03 | 96.81 | 95.03 | 39.88 | 2364778 | 2265258 | 95.79 | 12.64 | 11.16 | 6.06 |
| G371 | 309227616 | 309187296 | 99.99 | 0.03 | 96.28 | 94.25 | 39.95 | 2147134 | 2080001 | 96.87 | 11.73 | 11.10 | 5.92 |
| G372 | 324313056 | 324265248 | 99.99 | 0.03 | 94.56 | 91.56 | 40.03 | 2251842 | 2177878 | 96.72 | 12.58 | 10.78 | 5.85 |
| G373 | 307399392 | 307357056 | 99.99 | 0.03 | 95.00 | 92.23 | 40.05 | 2134424 | 2067929 | 96.88 | 11.98 | 10.89 | 5.77 |
| G374 | 362321568 | 362269440 | 99.99 | 0.03 | 95.77 | 93.43 | 39.98 | 2515760 | 2441265 | 97.04 | 13.92 | 10.74 | 6.05 |
| G375 | 350857440 | 350811072 | 99.99 | 0.03 | 94.39 | 91.30 | 40.12 | 2436188 | 2362926 | 96.99 | 12.15 | 12.11 | 6.52 |
| G376 | 313297632 | 313256448 | 99.99 | 0.03 | 96.81 | 95.05 | 39.77 | 2175392 | 2107645 | 96.89 | 12.28 | 10.66 | 5.80 |
| G377 | 318360960 | 318311136 | 99.98 | 0.03 | 96.97 | 95.28 | 39.77 | 2210494 | 2146917 | 97.12 | 12.32 | 10.74 | 5.89 |
| G378 | 321721632 | 321676416 | 99.99 | 0.03 | 95.77 | 93.42 | 40.02 | 2233864 | 2164833 | 96.91 | 12.65 | 10.69 | 5.78 |
| G379 | 298269504 | 298224288 | 99.98 | 0.03 | 96.11 | 93.98 | 39.82 | 2071002 | 2004652 | 96.80 | 11.96 | 10.32 | 5.65 |
| G380 | 291765312 | 291721824 | 99.99 | 0.03 | 94.95 | 92.17 | 40.03 | 2025846 | 1961213 | 96.81 | 12.71 | 9.42 | 5.26 |
| G381 | 340790400 | 340733664 | 99.98 | 0.03 | 94.58 | 91.59 | 40.03 | 2366206 | 2284673 | 96.55 | 12.52 | 11.29 | 6.17 |
| G382 | 336550752 | 336507264 | 99.99 | 0.03 | 95.87 | 93.59 | 39.72 | 2336856 | 2264282 | 96.89 | 12.88 | 10.78 | 6.00 |
| G383 | 311612256 | 311568192 | 99.99 | 0.03 | 94.97 | 92.23 | 39.77 | 2163668 | 2096734 | 96.91 | 12.96 | 9.68 | 5.55 |
| G384 | 283122432 | 283088736 | 99.99 | 0.04 | 92.72 | 88.63 | 40.34 | 1965894 | 1895839 | 96.44 | 12.04 | 9.72 | 5.31 |
| G385 | 342741024 | 342683136 | 99.98 | 0.03 | 96.06 | 93.89 | 39.84 | 2379744 | 2309577 | 97.05 | 12.94 | 10.86 | 6.08 |
| G386 | 322456032 | 322405632 | 99.98 | 0.03 | 95.87 | 93.59 | 40.03 | 2238928 | 2155738 | 96.28 | 12.55 | 10.76 | 5.80 |
| G387 | 610382592 | 610348608 | 99.99 | 0.03 | 96.78 | 94.89 | 39.31 | 4238532 | 4107146 | 96.90 | 20.99 | 11.96 | 6.97 |
| G388 | 490603680 | 490571424 | 99.99 | 0.03 | 96.73 | 94.81 | 39.78 | 3406746 | 3299104 | 96.84 | 17.14 | 11.79 | 6.76 |
| G389 | 484308864 | 484280928 | 99.99 | 0.03 | 96.39 | 94.22 | 39.91 | 3363062 | 3262558 | 97.01 | 16.67 | 12.03 | 6.87 |
| G390 | 509390208 | 509354496 | 99.99 | 0.02 | 97.06 | 95.37 | 39.48 | 3537184 | 3424776 | 96.82 | 17.46 | 12.15 | 6.90 |
| G391 | 479867616 | 479831328 | 99.99 | 0.02 | 96.98 | 95.24 | 39.66 | 3332162 | 3233621 | 97.04 | 17.00 | 11.96 | 6.64 |
| G392 | 425941056 | 425915712 | 99.99 | 0.03 | 96.72 | 94.81 | 40.02 | 2957748 | 2861762 | 96.75 | 14.43 | 12.46 | 6.81 |
| G393 | 532268352 | 532234368 | 99.99 | 0.03 | 95.94 | 93.47 | 40.20 | 3696072 | 3571292 | 96.62 | 14.83 | 14.46 | 8.31 |
| G394 | 508667040 | 508627584 | 99.99 | 0.02 | 97.51 | 96.12 | 39.86 | 3532136 | 3433131 | 97.20 | 14.47 | 14.32 | 8.20 |
| G395 | 517769568 | 517736160 | 99.99 | 0.03 | 96.91 | 95.12 | 39.94 | 3595390 | 3485519 | 96.94 | 15.01 | 14.16 | 8.03 |
| G396 | 567995904 | 567956736 | 99.99 | 0.02 | 97.27 | 95.69 | 40.04 | 3944144 | 3823731 | 96.95 | 15.31 | 14.83 | 8.67 |
| G397 | 483112224 | 483083136 | 99.99 | 0.02 | 97.12 | 95.44 | 40.01 | 3354744 | 3254388 | 97.01 | 14.26 | 13.89 | 7.85 |
| G398 | 392637312 | 392610528 | 99.99 | 0.03 | 96.50 | 94.45 | 39.70 | 2726462 | 2647925 | 97.12 | 14.80 | 11.03 | 6.20 |
| G399 | 554920416 | 554882400 | 99.99 | 0.03 | 96.47 | 94.35 | 39.94 | 3853350 | 3731376 | 96.83 | 12.51 | 17.32 | 10.15 |
| G400 | 557361504 | 557322048 | 99.99 | 0.03 | 96.30 | 94.06 | 40.27 | 3870292 | 3752281 | 96.95 | 14.32 | 15.37 | 9.05 |
| G401 | 574224480 | 574180992 | 99.99 | 0.03 | 96.20 | 93.91 | 40.16 | 3987368 | 3865735 | 96.95 | 14.55 | 15.49 | 9.22 |
| G402 | 583002720 | 582968160 | 99.99 | 0.03 | 96.83 | 94.96 | 40.08 | 4048390 | 3917036 | 96.76 | 15.37 | 15.03 | 8.88 |
| G403 | 543675168 | 543638016 | 99.99 | 0.02 | 97.27 | 95.70 | 39.94 | 3775264 | 3657866 | 96.89 | 15.18 | 14.55 | 8.35 |
| G404 | 479886912 | 479852640 | 99.99 | 0.03 | 96.87 | 95.05 | 40.03 | 3332310 | 3233262 | 97.03 | 15.03 | 13.29 | 7.43 |
| G405 | 483525792 | 483498720 | 99.99 | 0.03 | 96.77 | 94.88 | 39.98 | 3357630 | 3255963 | 96.97 | 15.28 | 13.14 | 7.37 |
| G406 | 490896576 | 490862016 | 99.99 | 0.02 | 97.45 | 95.99 | 39.82 | 3408764 | 3273770 | 96.04 | 15.41 | 13.10 | 7.36 |
| G407 | 468609696 | 468579168 | 99.99 | 0.02 | 97.08 | 95.39 | 39.98 | 3254022 | 3160664 | 97.13 | 14.52 | 13.38 | 7.49 |
| G408 | 493151616 | 493120800 | 99.99 | 0.03 | 95.81 | 93.26 | 40.12 | 3424450 | 3317086 | 96.86 | 15.33 | 13.26 | 7.48 |
| G409 | 489717792 | 489682368 | 99.99 | 0.03 | 96.12 | 93.77 | 40.11 | 3400572 | 3304031 | 97.16 | 15.67 | 12.98 | 7.33 |
| G410 | 446747328 | 446713632 | 99.99 | 0.03 | 96.70 | 94.74 | 40.09 | 3102178 | 3010807 | 97.05 | 15.14 | 12.40 | 6.88 |
| G411 | 492262848 | 492259680 | 100.00 | 0.03 | 96.00 | 93.54 | 40.16 | 3418470 | 3318094 | 97.06 | 17.23 | 11.57 | 6.77 |
| G412 | 497259360 | 497257344 | 100.00 | 0.03 | 95.87 | 93.30 | 40.47 | 3453176 | 3349194 | 96.99 | 15.99 | 12.60 | 7.27 |
| G413 | 383791104 | 383789952 | 100.00 | 0.03 | 95.76 | 93.14 | 40.28 | 2665208 | 2589344 | 97.15 | 13.49 | 11.80 | 6.56 |
| G414 | 382560768 | 382557312 | 100.00 | 0.03 | 96.53 | 94.46 | 40.37 | 2656648 | 2576436 | 96.98 | 13.93 | 11.33 | 6.34 |
| G415 | 356410656 | 356408352 | 100.00 | 0.03 | 97.10 | 95.40 | 40.11 | 2475058 | 2401728 | 97.04 | 13.26 | 11.13 | 6.17 |
| G416 | 351093888 | 351091296 | 100.00 | 0.03 | 96.58 | 94.55 | 40.15 | 2438134 | 2362087 | 96.88 | 14.07 | 10.20 | 5.78 |
| G417 | 367227360 | 367224480 | 100.00 | 0.03 | 96.48 | 94.39 | 40.09 | 2550170 | 2472325 | 96.95 | 13.55 | 11.29 | 6.21 |
| G418 | 360766080 | 360764064 | 100.00 | 0.02 | 97.34 | 95.76 | 39.95 | 2505306 | 2431308 | 97.05 | 13.67 | 10.83 | 6.10 |
| G419 | 353883168 | 353882016 | 100.00 | 0.03 | 96.87 | 95.00 | 40.15 | 2457514 | 2388295 | 97.18 | 13.86 | 10.35 | 5.94 |
| G420 | 351570816 | 351569664 | 100.00 | 0.03 | 95.34 | 92.47 | 40.53 | 2441456 | 2356697 | 96.53 | 12.90 | 11.46 | 6.15 |
| G421 | 389242080 | 389239488 | 100.00 | 0.03 | 95.75 | 93.14 | 40.36 | 2703052 | 2621810 | 96.99 | 14.43 | 11.11 | 6.27 |
| G422 | 402541344 | 402539904 | 100.00 | 0.03 | 96.47 | 94.34 | 40.24 | 2795416 | 2713099 | 97.06 | 13.61 | 12.16 | 6.82 |
| G423 | 388196064 | 388195200 | 100.00 | 0.03 | 95.16 | 92.12 | 40.45 | 2695800 | 2613935 | 96.96 | 14.53 | 10.94 | 6.21 |
| G424 | 413892864 | 413889696 | 100.00 | 0.02 | 97.36 | 95.81 | 40.03 | 2874234 | 2780593 | 96.74 | 14.98 | 11.29 | 6.44 |
| G425 | 378430560 | 378426816 | 100.00 | 0.02 | 97.48 | 96.01 | 39.95 | 2627964 | 2553046 | 97.15 | 14.93 | 10.17 | 5.98 |
| G426 | 381544992 | 381542400 | 100.00 | 0.03 | 96.48 | 94.36 | 40.27 | 2649600 | 2572049 | 97.07 | 14.25 | 11.08 | 6.22 |
| G427 | 357366528 | 357364512 | 100.00 | 0.03 | 96.72 | 94.76 | 40.20 | 2481698 | 2413902 | 97.27 | 14.17 | 10.19 | 5.91 |
| G428 | 366634368 | 366631488 | 100.00 | 0.03 | 95.70 | 93.05 | 40.50 | 2546052 | 2468006 | 96.93 | 14.08 | 10.78 | 6.02 |
| G429 | 355620960 | 355618944 | 100.00 | 0.03 | 95.28 | 92.34 | 40.38 | 2469576 | 2367852 | 95.88 | 13.39 | 10.78 | 6.03 |
| G430 | 410134176 | 410130432 | 100.00 | 0.03 | 96.44 | 94.32 | 40.09 | 2848128 | 2727633 | 95.77 | 15.44 | 10.58 | 6.16 |
| G431 | 376628544 | 376626240 | 100.00 | 0.03 | 95.59 | 92.89 | 40.28 | 2615460 | 2509110 | 95.93 | 14.35 | 10.59 | 6.03 |
| G432 | 375057504 | 375054912 | 100.00 | 0.03 | 93.69 | 89.46 | 40.44 | 2604548 | 2501830 | 96.06 | 15.34 | 9.71 | 5.70 |
| G433 | 457734240 | 457729632 | 100.00 | 0.03 | 96.68 | 94.71 | 40.09 | 3178678 | 3039786 | 95.63 | 16.30 | 11.31 | 6.51 |
| G434 | 442190880 | 442187136 | 100.00 | 0.03 | 96.53 | 94.47 | 40.14 | 3070744 | 2952991 | 96.17 | 15.63 | 11.50 | 6.55 |
| G435 | 483113952 | 483107040 | 100.00 | 0.03 | 95.80 | 89.93 | 39.45 | 3354910 | 3251330 | 96.91 | 16.89 | 13.24 | 6.55 |
| G436 | 419280768 | 419273280 | 100.00 | 0.03 | 96.07 | 90.50 | 40.00 | 2911620 | 2829160 | 97.17 | 14.47 | 13.07 | 6.56 |
| G437 | 412297344 | 412290720 | 100.00 | 0.03 | 95.91 | 90.12 | 40.27 | 2863130 | 2781310 | 97.14 | 14.48 | 12.12 | 6.58 |
| G438 | 424120896 | 424115424 | 100.00 | 0.03 | 96.08 | 90.56 | 39.72 | 2945246 | 2830956 | 96.12 | 15.77 | 11.68 | 6.15 |
| G439 | 433144800 | 433137600 | 100.00 | 0.03 | 96.13 | 90.69 | 39.79 | 3007900 | 2925889 | 97.27 | 15.06 | 13.41 | 6.49 |
| G440 | 402114816 | 402111648 | 100.00 | 0.03 | 96.12 | 90.56 | 40.38 | 2792442 | 2708193 | 96.98 | 13.66 | 12.36 | 6.74 |
| G441 | 400825152 | 400819104 | 100.00 | 0.03 | 95.48 | 89.14 | 39.94 | 2783466 | 2700189 | 97.01 | 14.27 | 12.77 | 6.33 |
| G442 | 429485184 | 429476256 | 100.00 | 0.03 | 96.04 | 90.59 | 40.28 | 2982474 | 2897804 | 97.16 | 14.64 | 12.49 | 6.78 |
| G443 | 433469664 | 433460736 | 100.00 | 0.03 | 95.59 | 89.49 | 40.07 | 3010144 | 2920031 | 97.01 | 15.61 | 11.87 | 6.44 |
| G444 | 453509568 | 453502080 | 100.00 | 0.03 | 96.07 | 90.53 | 40.31 | 3149320 | 3056838 | 97.06 | 14.90 | 12.99 | 7.01 |
| G445 | 430505856 | 430502112 | 100.00 | 0.03 | 96.16 | 90.70 | 40.47 | 2989598 | 2897815 | 96.93 | 14.67 | 12.20 | 6.80 |
| G446 | 499198752 | 499193280 | 100.00 | 0.03 | 95.92 | 90.08 | 39.79 | 3466620 | 3358470 | 96.88 | 17.23 | 12.78 | 6.72 |
| G447 | 414733536 | 414727488 | 100.00 | 0.03 | 95.50 | 89.16 | 40.00 | 2880052 | 2790180 | 96.88 | 15.15 | 11.69 | 6.33 |
| G448 | 347617152 | 347613408 | 100.00 | 0.03 | 95.94 | 90.10 | 40.40 | 2413982 | 2338772 | 96.88 | 12.98 | 11.24 | 6.11 |
| G449 | 381722688 | 381717792 | 100.00 | 0.03 | 95.65 | 89.50 | 40.36 | 2650818 | 2572369 | 97.04 | 13.67 | 12.04 | 6.37 |
| G450 | 385385760 | 385380288 | 100.00 | 0.03 | 95.99 | 90.30 | 40.41 | 2676252 | 2578957 | 96.36 | 13.58 | 11.91 | 6.45 |
| G451 | 441566496 | 441558720 | 100.00 | 0.03 | 96.06 | 90.64 | 40.34 | 3066380 | 2972387 | 96.93 | 14.90 | 12.40 | 6.87 |
| G452 | 396896256 | 396890784 | 100.00 | 0.03 | 96.03 | 90.44 | 40.14 | 2756186 | 2668481 | 96.82 | 14.05 | 12.41 | 6.40 |
| G453 | 429041088 | 429036480 | 100.00 | 0.03 | 95.95 | 90.31 | 40.06 | 2979420 | 2892544 | 97.08 | 14.78 | 12.66 | 6.66 |
| G454 | 443905056 | 443897856 | 100.00 | 0.03 | 95.94 | 90.44 | 40.17 | 3082624 | 2980633 | 96.69 | 14.46 | 13.70 | 6.92 |
| G455 | 412303680 | 412294464 | 100.00 | 0.03 | 96.08 | 90.59 | 40.33 | 2863156 | 2775296 | 96.93 | 14.75 | 11.68 | 6.48 |
| G456 | 465295680 | 465285888 | 100.00 | 0.03 | 95.48 | 89.12 | 39.94 | 3231152 | 3061540 | 94.75 | 15.54 | 12.72 | 6.75 |
| G457 | 451826496 | 451821024 | 100.00 | 0.03 | 95.77 | 89.81 | 40.20 | 3137646 | 3032991 | 96.66 | 15.78 | 11.90 | 6.67 |
| G458 | 445864608 | 445858272 | 100.00 | 0.03 | 96.20 | 90.78 | 40.31 | 3096238 | 2995145 | 96.73 | 15.89 | 11.41 | 6.59 |
| G459 | 438407136 | 438401664 | 100.00 | 0.03 | 95.79 | 89.77 | 40.13 | 3044456 | 2951042 | 96.93 | 15.80 | 11.23 | 6.53 |
| G460 | 427702176 | 427694688 | 100.00 | 0.03 | 96.05 | 90.62 | 40.42 | 2970102 | 2867611 | 96.55 | 14.34 | 12.50 | 6.83 |
| G461 | 420135264 | 420128640 | 100.00 | 0.03 | 96.06 | 90.66 | 40.43 | 2917560 | 2808747 | 96.27 | 14.50 | 11.88 | 6.66 |
| G462 | 428618592 | 428611968 | 100.00 | 0.03 | 96.04 | 90.44 | 40.33 | 2976472 | 2884091 | 96.90 | 14.40 | 12.70 | 6.84 |
| G463 | 426876768 | 426871584 | 100.00 | 0.03 | 96.04 | 90.50 | 40.03 | 2964386 | 2856834 | 96.37 | 14.20 | 13.65 | 6.71 |
| G464 | 417826368 | 417820896 | 100.00 | 0.03 | 95.80 | 89.84 | 40.23 | 2901534 | 2813617 | 96.97 | 14.33 | 12.72 | 6.66 |
| G465 | 427836672 | 427828896 | 100.00 | 0.03 | 95.31 | 88.75 | 39.83 | 2971034 | 2868126 | 96.54 | 15.72 | 11.34 | 6.32 |
| G466 | 462703968 | 462696768 | 100.00 | 0.03 | 95.98 | 90.30 | 40.08 | 3213172 | 3116163 | 96.98 | 15.26 | 13.21 | 6.95 |
| G467 | 453149856 | 453144384 | 100.00 | 0.03 | 95.83 | 89.90 | 39.95 | 3146836 | 3042325 | 96.68 | 14.55 | 13.90 | 7.04 |
| G468 | 419159232 | 419153184 | 100.00 | 0.04 | 94.48 | 86.89 | 39.91 | 2910786 | 2823403 | 97.00 | 15.60 | 11.01 | 6.31 |
| G469 | 429165792 | 429160032 | 100.00 | 0.03 | 95.91 | 90.21 | 40.24 | 2980278 | 2893827 | 97.10 | 15.19 | 11.99 | 6.57 |
