# Supplementary material for: Mapping QTLs for anaerobic tolerance at germination and bud stages using new high density genetic map of rice
Source: Front Plant Sci. 2022 Oct 17;13:985080. doi: 10.3389/fpls.2022.985080 (PMC9618957; doi:10.3389/fpls.2022.985080)
Supplement: Supplementary file 6 [file Table_3.docx]

| **Supplementary Table S3** Phenotypic data of all samples (wet season). | | | | | | |  |  |  |  |  |  |
| --- | --- | --- | --- | --- | --- | --- | --- | --- | --- | --- | --- | --- |
| **RILs** | **CL (cm)** | **CSA（cm^2^）** | **CV（mm^3^）** | **CD（mm）** | **SSD（mm）** | **SH（cm）** | **SFW (mg)** | **RL（cm）** | **RSA（cm^2^）** | **RV（mm^3^）** | **RD（mm）** | **RFW (mg)** |
| G195 | 2.6691 | 0.5315 | 8.5098 | 0.6362 | 0.6886 | 5.1878 | 14.7633 | 3.7880 | 0.5029 | 5.3667 | 0.4432 | 6.6467 |
| G196 | 2.6852 | 0.4520 | 6.0718 | 0.5342 | 0.7019 | 3.1099 | 11.9459 | 2.8301 | 0.4389 | 5.4250 | 0.4942 | 5.6400 |
| G197 | 2.6100 | 0.4938 | 7.4615 | 0.5967 | 0.7053 | 4.4774 | 15.0296 | 2.7537 | 0.4215 | 5.1333 | 0.4870 | 7.0000 |
| G198 | 2.6711 | 0.5313 | 8.4211 | 0.6314 | 0.7976 | 3.5479 | 17.0400 | 1.8357 | 0.3045 | 4.0167 | 0.5289 | 5.2400 |
| G199 | 2.5927 | 0.4614 | 6.5409 | 0.5663 | 0.6899 | 3.9903 | 14.5944 | 2.4877 | 0.3874 | 4.4185 | 0.4944 | 5.5395 |
| G200 | 2.9724 | 0.5652 | 8.5728 | 0.6004 | 0.7211 | 5.8551 | 20.8600 | 2.8823 | 0.3986 | 4.1963 | 0.4408 | 6.8544 |
| G201 | 2.5504 | 0.4691 | 6.8780 | 0.5833 | 0.6993 | 2.5261 | 10.2767 | 1.8554 | 0.2851 | 3.5000 | 0.5014 | 3.9900 |
| G202 | 2.5651 | 0.4731 | 6.9678 | 0.5884 | 0.6525 | 5.2379 | 16.6104 | 2.8114 | 0.4178 | 4.9296 | 0.4731 | 6.4689 |
| G203 | 2.3416 | 0.4358 | 6.4649 | 0.5924 | 0.6979 | 4.8177 | 15.5274 | 2.6920 | 0.3989 | 4.7833 | 0.4863 | 5.5825 |
| G204 | 2.6635 | 0.5385 | 8.7254 | 0.6411 | 0.6984 | 5.9736 | 19.9700 | 3.0591 | 0.4313 | 4.8852 | 0.4570 | 6.6070 |
| G205 | 2.8220 | 0.5346 | 8.1362 | 0.6054 | 0.6294 | 5.4572 | 15.8719 | 2.3860 | 0.3490 | 3.9259 | 0.4652 | 4.9163 |
| G206 | 2.6202 | 0.4792 | 6.9889 | 0.5836 | 0.7636 | 6.7736 | 20.3956 | 2.9713 | 0.4174 | 4.6815 | 0.4482 | 6.5400 |
| G207 | 2.5196 | 0.4370 | 6.0482 | 0.5518 | 0.6846 | 3.5662 | 11.4200 | 2.8373 | 0.3844 | 4.1667 | 0.4333 | 5.1833 |
| G208 | 2.9364 | 0.5589 | 8.4956 | 0.6057 | 0.7403 | 4.8624 | 15.5974 | 3.1870 | 0.4358 | 4.6667 | 0.4544 | 3.1250 |
| G209 | 2.4770 | 0.4089 | 5.3960 | 0.5259 | 0.6424 | 2.7137 | 9.2430 | 2.1123 | 0.3175 | 3.8333 | 0.4864 | 4.9475 |
| G210 | 2.7656 | 0.5467 | 8.6455 | 0.6238 | 0.7777 | 4.8389 | 17.6900 | 2.6832 | 0.4027 | 4.8296 | 0.4899 | 6.3470 |
| G211 | 3.0278 | 0.5324 | 7.4595 | 0.5596 | 0.6902 | 3.3344 | 12.0200 | 2.1131 | 0.3279 | 4.0667 | 0.4949 | 4.9667 |
| G212 | 2.9063 | 0.5463 | 8.1764 | 0.5981 | 0.7521 | 4.4109 | 14.6463 | 2.3035 | 0.3538 | 4.2074 | 0.4919 | 5.7569 |
| G213 | 2.5192 | 0.4818 | 7.3465 | 0.6097 | 0.7015 | 3.6207 | 11.9794 | 2.7598 | 0.3687 | 3.7974 | 0.4411 | 5.4204 |
| G214 | 2.4201 | 0.4764 | 7.4629 | 0.6273 | 0.8428 | 4.6628 | 17.9259 | 4.4856 | 0.6387 | 7.0944 | 0.4750 | 8.8569 |
| G215 | 2.6724 | 0.5338 | 8.5040 | 0.6330 | 0.7573 | 3.9160 | 13.2770 | 2.4399 | 0.3922 | 4.7963 | 0.5120 | 6.5730 |
| G216 | 2.8895 | 0.5221 | 7.5074 | 0.5752 | 0.8440 | 5.6006 | 16.2652 | 4.0562 | 0.5067 | 5.0037 | 0.4236 | 8.5430 |
| G217 | 2.5487 | 0.4608 | 6.6185 | 0.5748 | 0.7212 | 2.5029 | 11.5437 | 2.5861 | 0.3990 | 4.9333 | 0.4888 | 5.6874 |
| G218 | 2.0687 | 0.3636 | 5.0857 | 0.5591 | 0.7052 | 3.3248 | 12.9170 | 2.1349 | 0.3511 | 4.4185 | 0.5245 | 5.8178 |
| G219 | 2.5945 | 0.5041 | 7.7798 | 0.6190 | 0.6600 | 4.1713 | 12.2313 | 3.1030 | 0.4212 | 4.0357 | 0.4456 | 5.4232 |
| G220 | 2.6865 | 0.4741 | 6.6640 | 0.5613 | 0.6992 | 4.5453 | 14.0675 | 2.3278 | 0.3222 | 3.2704 | 0.4548 | 4.2702 |
| G221 | 2.5002 | 0.4674 | 6.9510 | 0.5950 | 0.7130 | 4.8148 | 15.3282 | 2.6180 | 0.3913 | 4.6741 | 0.4886 | 6.8152 |
| G222 | 2.5890 | 0.4587 | 6.4842 | 0.5635 | 0.6466 | 3.6073 | 11.7496 | 1.6821 | 0.2387 | 2.7101 | 0.4674 | 3.7201 |
| G223 | 2.9388 | 0.5750 | 8.9649 | 0.6205 | 0.6776 | 4.5870 | 15.6319 | 2.5862 | 0.3654 | 3.9444 | 0.4472 | 5.6439 |
| G224 | 3.0853 | 0.6374 | 10.4870 | 0.6569 | 0.7616 | 6.0364 | 21.5815 | 4.1710 | 0.5571 | 5.9704 | 0.4614 | 9.3370 |
| G225 | 2.7720 | 0.5212 | 7.7922 | 0.5973 | 0.7145 | 6.3596 | 19.0000 | 6.9868 | 0.6927 | 5.7000 | 0.3715 | 9.2367 |
| G226 | 2.2824 | 0.4148 | 6.0137 | 0.5786 | 0.6748 | 4.6242 | 13.9600 | 2.9303 | 0.3974 | 4.2667 | 0.4280 | 5.8667 |
| G227 | 2.4508 | 0.4637 | 7.0000 | 0.6029 | 0.7621 | 4.3653 | 15.0848 | 2.7700 | 0.3891 | 4.0333 | 0.4592 | 6.0805 |
| G228 | 3.1517 | 0.6445 | 10.4673 | 0.6499 | 0.7094 | 5.0247 | 14.8952 | 3.6191 | 0.4669 | 4.7667 | 0.4493 | 6.0356 |
| G229 | 2.5114 | 0.4242 | 5.7143 | 0.5381 | 0.6693 | 3.6396 | 11.5433 | 3.8706 | 0.5227 | 6.1778 | 0.4758 | 6.7081 |
| G230 | 2.6272 | 0.4733 | 6.7948 | 0.5726 | 0.7158 | 5.7422 | 17.5452 | 3.7656 | 0.4961 | 5.3704 | 0.4456 | 8.5593 |
| G231 | 2.3417 | 0.4065 | 5.6264 | 0.5521 | 0.6750 | 3.4885 | 12.2341 | 2.3403 | 0.3564 | 4.1444 | 0.4853 | 5.4519 |
| G232 | 2.4887 | 0.4472 | 6.3973 | 0.5733 | 0.7604 | 6.1555 | 18.9721 | 3.1323 | 0.4578 | 5.0000 | 0.4663 | 7.5750 |
| G233 | 2.6274 | 0.5005 | 7.5965 | 0.6057 | 0.6792 | 4.8037 | 13.1067 | 2.1330 | 0.3184 | 3.6667 | 0.4784 | 4.1370 |
| G234 | 2.5475 | 0.4600 | 6.6221 | 0.5745 | 0.7207 | 2.3014 | 9.7556 | 1.9602 | 0.2931 | 3.3889 | 0.4773 | 4.0526 |
| G235 | 2.6153 | 0.4913 | 7.3385 | 0.5987 | 0.7363 | 4.2796 | 15.0257 | 3.0924 | 0.3927 | 4.0127 | 0.4208 | 5.3894 |
| G236 | 3.0390 | 0.6027 | 9.5346 | 0.6301 | 0.7037 | 6.1103 | 20.2800 | 2.8526 | 0.4148 | 4.8500 | 0.4650 | 7.0450 |
| G237 | 2.4465 | 0.4266 | 5.9106 | 0.5556 | 0.6525 | 2.8894 | 10.5867 | 2.3255 | 0.3553 | 4.3250 | 0.4893 | 5.2533 |
| G238 | 2.3271 | 0.4217 | 6.0925 | 0.5762 | 0.6923 | 4.2673 | 14.7922 | 2.5030 | 0.4130 | 5.1444 | 0.5256 | 6.9551 |
| G239 | 2.4706 | 0.4608 | 6.8599 | 0.5926 | 0.7454 | 4.2072 | 13.9470 | 2.8688 | 0.4251 | 5.0083 | 0.4990 | 6.4277 |
| G240 | 2.7785 | 0.5186 | 7.7258 | 0.5940 | 0.7015 | 6.1391 | 18.1978 | 2.9413 | 0.4130 | 4.4667 | 0.4579 | 6.8789 |
| G241 | 2.8223 | 0.5182 | 7.5847 | 0.5838 | 0.6806 | 4.4689 | 13.8496 | 1.9349 | 0.2863 | 3.3148 | 0.4661 | 4.2193 |
| G242 | 2.4173 | 0.3907 | 5.0446 | 0.5133 | 0.5737 | 4.7046 | 10.5542 | 3.5258 | 0.4147 | 3.9167 | 0.3766 | 6.0758 |
| G243 | 2.6940 | 0.4924 | 7.1754 | 0.5797 | 0.6065 | 5.5839 | 14.5400 | 3.2206 | 0.4265 | 4.3583 | 0.4280 | 7.4739 |
| G244 | 3.1909 | 0.6146 | 9.4027 | 0.6133 | 0.7706 | 5.9960 | 20.1763 | 2.3149 | 0.3397 | 3.7963 | 0.4825 | 5.9888 |
| G245 | 3.2362 | 0.5973 | 8.7905 | 0.5869 | 0.6747 | 5.1816 | 16.1458 | 2.1875 | 0.3305 | 3.6926 | 0.4810 | 6.2054 |
| G246 | 2.7858 | 0.5049 | 7.2869 | 0.5767 | 0.6644 | 5.8508 | 18.1757 | 3.3999 | 0.4380 | 4.5333 | 0.4134 | 6.4256 |
| G247 | 3.0684 | 0.5866 | 8.9246 | 0.6071 | 0.6871 | 7.5150 | 22.3370 | 6.2928 | 0.7241 | 6.3083 | 0.3842 | 8.6733 |
| G248 | 3.0250 | 0.5474 | 7.8997 | 0.5756 | 0.6690 | 3.7929 | 12.4706 | 1.7665 | 0.2704 | 3.2917 | 0.4864 | 4.5417 |
| G249 | 3.0536 | 0.5614 | 8.2431 | 0.5849 | 0.7190 | 5.1273 | 16.6900 | 4.1099 | 0.4965 | 4.8238 | 0.3976 | 7.8919 |
| G250 | 2.6302 | 0.4762 | 6.8671 | 0.5739 | 0.7025 | 4.7185 | 15.0657 | 2.0922 | 0.3049 | 3.2750 | 0.4634 | 5.4042 |
| G251 | 2.8463 | 0.5172 | 7.4992 | 0.5781 | 0.7056 | 6.2823 | 19.6650 | 3.1920 | 0.4164 | 4.3714 | 0.4399 | 19.1293 |
| G252 | 2.7189 | 0.5187 | 7.8863 | 0.6063 | 0.7214 | 5.2252 | 16.2633 | 2.4296 | 0.3488 | 3.9667 | 0.4587 | 5.5633 |
| G253 | 2.6280 | 0.4987 | 7.5417 | 0.6040 | 0.6691 | 6.0512 | 18.7717 | 2.9992 | 0.3796 | 3.5500 | 0.4139 | 6.9250 |
| G254 | 2.8732 | 0.5476 | 8.3129 | 0.6064 | 0.7209 | 6.1939 | 18.5670 | 2.8519 | 0.4062 | 4.4407 | 0.4548 | 5.1552 |
| G255 | 2.3464 | 0.4291 | 6.2811 | 0.5810 | 0.6725 | 4.0452 | 12.7194 | 2.3362 | 0.3163 | 3.1917 | 0.4424 | 4.4821 |
| G256 | 3.1249 | 0.5943 | 9.0260 | 0.6039 | 0.7289 | 4.7870 | 16.8711 | 3.2016 | 0.4815 | 5.8111 | 0.4809 | 6.1648 |
| G257 | 2.3288 | 0.3708 | 4.6913 | 0.5080 | 0.5863 | 4.5833 | 10.5956 | 2.6894 | 0.3378 | 3.1963 | 0.3983 | 4.3415 |
| G258 | 2.3409 | 0.3628 | 4.4805 | 0.4941 | 0.5733 | 3.5091 | 9.6317 | 3.0844 | 0.3601 | 3.1667 | 0.3712 | 4.5517 |
| G259 | 2.7647 | 0.4934 | 7.0421 | 0.5683 | 0.7627 | 5.3093 | 11.3181 | 3.5756 | 0.4843 | 5.1000 | 0.4294 | 4.1952 |
| G260 | 2.9486 | 0.5718 | 8.8447 | 0.6138 | 0.7055 | 6.1826 | 20.2733 | 4.0523 | 0.4703 | 4.3667 | 0.3697 | 7.1700 |
| G261 | 2.7571 | 0.5081 | 7.4491 | 0.5870 | 0.6929 | 5.2744 | 17.3800 | 7.1459 | 0.6537 | 5.3333 | 0.4065 | 8.5467 |
| G262 | 2.7634 | 0.5428 | 8.4965 | 0.6241 | 0.7524 | 6.2592 | 19.8493 | 7.0143 | 0.7238 | 6.0333 | 0.3845 | 8.7085 |
| G263 | 3.1299 | 0.6086 | 9.4659 | 0.6175 | 0.6688 | 4.5310 | 15.6235 | 2.5161 | 0.3351 | 3.2667 | 0.4218 | 4.6667 |
| G264 | 2.6480 | 0.4629 | 6.4423 | 0.5568 | 0.6116 | 5.3586 | 13.9242 | 2.7777 | 0.3879 | 4.3000 | 0.4456 | 5.8325 |
| G265 | 2.8180 | 0.5402 | 8.2542 | 0.6102 | 0.6985 | 6.3984 | 17.1770 | 3.8540 | 0.4902 | 4.7741 | 0.4044 | 7.3178 |
| G266 | 2.5853 | 0.4690 | 6.7955 | 0.5797 | 0.6200 | 3.5378 | 11.1485 | 2.0132 | 0.2731 | 2.8778 | 0.4309 | 4.0070 |
| G267 | 3.3526 | 0.6523 | 10.1482 | 0.6187 | 0.7037 | 6.4805 | 18.8963 | 3.3636 | 0.4924 | 5.8370 | 0.4761 | 7.8426 |
| G268 | 3.1728 | 0.6178 | 9.6349 | 0.6163 | 0.6532 | 5.0933 | 15.5950 | 2.2121 | 0.3280 | 3.0917 | 0.4707 | 4.9433 |
| G269 | 2.9082 | 0.6112 | 10.2736 | 0.6681 | 0.7032 | 5.1016 | 16.3126 | 2.8647 | 0.3919 | 4.2667 | 0.4369 | 6.1600 |
| G270 | 2.5161 | 0.5029 | 8.0064 | 0.6375 | 0.7572 | 3.7908 | 15.5900 | 3.4181 | 0.6126 | 9.0833 | 0.5445 | 6.9308 |
| G271 | NA | NA | NA | NA | NA | NA | NA | NA | NA | NA | NA | NA |
| G272 | 2.6504 | 0.4913 | 7.3555 | 0.5863 | 0.7496 | 2.9911 | 11.9043 | 1.8396 | 0.2885 | 3.6000 | 0.5022 | 3.5967 |
| G273 | 2.6801 | 0.5389 | 8.6361 | 0.6387 | 0.7435 | 5.9320 | 19.6730 | 2.5829 | 0.3657 | 4.0741 | 0.4622 | 6.4041 |
| G274 | 2.4950 | 0.4913 | 7.7488 | 0.6256 | 0.6573 | 4.1375 | 13.5104 | 2.0233 | 0.2927 | 3.0667 | 0.4636 | 4.2984 |
| G275 | 2.2943 | 0.4412 | 6.8143 | 0.6097 | 0.6450 | 5.6161 | 16.3874 | 4.9190 | 0.5242 | 4.4741 | 0.3800 | 3.8523 |
| G276 | 2.3013 | 0.4537 | 7.1393 | 0.6250 | 0.7044 | 3.0938 | 12.4900 | 1.9789 | 0.2917 | 3.4333 | 0.4719 | 4.6067 |
| G277 | 3.0204 | 0.6359 | 10.6679 | 0.6700 | 0.7615 | 4.9239 | 17.5975 | 1.8702 | 0.3008 | 3.6407 | 0.5158 | 5.7550 |
| G278 | 2.8435 | 0.5251 | 7.7624 | 0.5897 | 0.6880 | 5.2232 | 16.6158 | 3.8658 | 0.4634 | 4.2037 | 0.4144 | 4.3193 |
| G279 | 2.4779 | 0.4771 | 7.3250 | 0.6130 | 0.7877 | 2.2888 | 11.8533 | 1.8216 | 0.2841 | 3.5833 | 0.5019 | 3.1994 |
| G280 | 2.3820 | 0.4304 | 6.2180 | 0.5752 | 0.7094 | 4.4372 | 15.1751 | 2.0402 | 0.3099 | 3.6545 | 0.5072 | 6.3968 |
| G281 | 2.7654 | 0.5163 | 7.7317 | 0.5937 | 0.6823 | 3.8965 | 14.8567 | 4.6885 | 0.5308 | 4.9667 | 0.4201 | 6.6285 |
| G282 | 2.4281 | 0.4135 | 5.6122 | 0.5418 | 0.6349 | 5.0475 | 13.4933 | 3.7895 | 0.4432 | 4.1667 | 0.3914 | 5.7133 |
| G283 | 2.4373 | 0.4293 | 6.0311 | 0.5588 | 0.6106 | 5.3014 | 15.0374 | 2.3835 | 0.3200 | 3.4889 | 0.4388 | 5.2007 |
| G284 | 2.4860 | 0.4895 | 7.6962 | 0.6268 | 0.7544 | 5.7057 | 18.8833 | 5.4206 | 0.6129 | 5.4778 | 0.4207 | 8.9789 |
| G285 | 2.4916 | 0.4494 | 6.4568 | 0.5743 | 0.6762 | 6.3185 | 17.6130 | 4.7079 | 0.5532 | 5.1333 | 0.4024 | 7.6463 |
| G286 | 2.8271 | 0.5177 | 7.5418 | 0.5844 | 0.6162 | 5.2784 | 16.6467 | 5.5848 | 0.5439 | 4.5148 | 0.3796 | 7.1663 |
| G287 | 2.4348 | 0.4628 | 7.0140 | 0.6037 | 0.6876 | 4.5793 | 13.5367 | 2.2419 | 0.3011 | 3.2083 | 0.4251 | 4.5742 |
| G288 | 3.3823 | 0.6488 | 9.9222 | 0.6097 | 0.7211 | 5.2774 | 16.5867 | 3.1877 | 0.3893 | 3.9667 | 0.4189 | 6.7867 |
| G289 | 2.2232 | 0.4025 | 5.8320 | 0.5763 | 0.6780 | 6.6843 | 29.3900 | 6.4819 | 0.6708 | 5.7917 | 0.3919 | 7.9033 |
| G290 | 3.1096 | 0.6341 | 10.2865 | 0.6484 | 0.6789 | 6.1385 | 14.2407 | 3.2498 | 0.4570 | 5.0074 | 0.4561 | 6.0659 |
| G291 | 2.8311 | 0.5150 | 7.4768 | 0.5777 | 0.6430 | 5.0359 | 15.2156 | 6.2758 | 0.5767 | 4.2296 | 0.2993 | 6.6267 |
| G292 | 2.5979 | 0.4708 | 6.7833 | 0.5772 | 0.7281 | 6.0074 | 18.9122 | 3.0585 | 0.4263 | 4.7852 | 0.4632 | 7.7274 |
| G293 | 3.3589 | 0.6522 | 10.1077 | 0.6169 | 0.6504 | 6.2947 | 16.6768 | 2.7780 | 0.3895 | 3.8917 | 0.4469 | 5.7090 |
| G294 | 2.6882 | 0.4441 | 5.8451 | 0.5256 | 0.6851 | 3.9563 | 12.2740 | 2.3295 | 0.3429 | 3.7065 | 0.4686 | 5.6476 |
| G295 | 2.4950 | 0.4488 | 6.4503 | 0.5726 | 0.6424 | 4.7059 | 14.6970 | 2.3631 | 0.3367 | 3.2222 | 0.4547 | 5.6506 |
| G296 | 2.6821 | 0.4859 | 7.0044 | 0.5754 | 0.6911 | 6.5346 | 20.1533 | 5.4368 | 0.6021 | 5.4556 | 0.3767 | 5.0479 |
| G297 | 2.3796 | 0.4217 | 5.9442 | 0.5641 | 0.7220 | 3.9356 | 13.7181 | 2.1864 | 0.3301 | 3.8704 | 0.4861 | 5.0530 |
| G298 | 3.0080 | 0.5513 | 8.0518 | 0.5823 | 0.6971 | 4.8342 | 15.5733 | 2.3734 | 0.3578 | 4.3074 | 0.4773 | 6.0167 |
| G299 | 2.7044 | 0.5047 | 7.5000 | 0.5936 | 0.7064 | 4.7139 | 16.1889 | 2.5419 | 0.3706 | 4.1905 | 0.4679 | 6.1556 |
| G300 | 2.8546 | 0.5301 | 7.8509 | 0.5909 | 0.6996 | 5.7591 | 17.1400 | 3.0902 | 0.4358 | 4.9333 | 0.4511 | 7.2028 |
| G301 | 2.3863 | 0.3917 | 5.1349 | 0.5233 | 0.6646 | 3.6769 | 12.0041 | 2.5335 | 0.3824 | 4.6037 | 0.4786 | 5.9515 |
| G302 | 2.6897 | 0.4916 | 7.1507 | 0.5822 | 0.6611 | 4.3903 | 14.2433 | 2.5139 | 0.3700 | 4.2333 | 0.4726 | 5.4641 |
| G303 | 2.4915 | 0.4574 | 6.6993 | 0.5839 | 0.7694 | 6.2578 | 17.0567 | 6.1502 | 0.7223 | 6.8333 | 0.3899 | 9.4567 |
| G304 | 2.4371 | 0.4644 | 7.0820 | 0.6035 | 0.6622 | 3.9108 | 11.7281 | 2.7272 | 0.3829 | 4.1333 | 0.4470 | 5.1863 |
| G305 | 2.4544 | 0.4482 | 6.5299 | 0.5810 | 0.7246 | 2.7037 | 9.4233 | 2.1510 | 0.2990 | 3.3000 | 0.4432 | 3.8400 |
| G306 | 2.8011 | 0.5095 | 7.3699 | 0.5788 | 0.7982 | 5.6279 | 19.5133 | 5.5240 | 0.6226 | 5.7333 | 0.3963 | 8.5400 |
| G307 | 2.7316 | 0.4841 | 6.8210 | 0.5647 | 0.6686 | 4.0857 | 12.4300 | 2.0188 | 0.3355 | 4.3000 | 0.5321 | 4.9167 |
| G308 | 2.8836 | 0.5552 | 8.5037 | 0.6124 | 0.8013 | 5.7858 | 18.8314 | 3.6530 | 0.4437 | 4.4190 | 0.4236 | 6.7362 |
| G309 | 2.7040 | 0.5047 | 7.4926 | 0.5928 | 0.6926 | 5.1507 | 23.1354 | 2.4229 | 0.3873 | 4.7810 | 0.5151 | 8.7833 |
| G310 | 2.8631 | 0.5474 | 8.3259 | 0.6078 | 0.6409 | 4.6946 | 14.8059 | 2.3782 | 0.3529 | 4.1926 | 0.4836 | 5.6300 |
| G311 | 2.6342 | 0.4907 | 7.2760 | 0.5923 | 0.8121 | 6.2732 | 20.0611 | 2.9576 | 0.4594 | 5.5667 | 0.5059 | 8.8033 |
| G312 | 2.3657 | 0.4544 | 6.9737 | 0.6125 | 0.7041 | 3.5567 | 14.0915 | 2.1407 | 0.2906 | 3.1583 | 0.4490 | 3.9700 |
| G313 | 2.7632 | 0.5145 | 7.6440 | 0.5932 | 0.7322 | 5.9174 | 18.4067 | 2.4301 | 0.3713 | 4.5429 | 0.4924 | 6.0481 |
| G314 | 2.9276 | 0.4983 | 6.7680 | 0.5408 | 0.6574 | 5.1410 | 15.0648 | 3.3975 | 0.4693 | 5.1889 | 0.4506 | 7.0433 |
| G315 | 2.7454 | 0.5258 | 8.0175 | 0.6099 | 0.7193 | 4.3088 | 13.8856 | 2.0780 | 0.2999 | 3.3333 | 0.4476 | 4.9724 |
| G316 | 2.6640 | 0.4943 | 7.2939 | 0.5910 | 0.6988 | 6.6622 | 19.6167 | 4.9753 | 0.6045 | 6.0000 | 0.4158 | 8.7833 |
| G317 | 2.6961 | 0.5088 | 7.6444 | 0.6010 | 0.6972 | 5.8029 | 17.0067 | 2.8115 | 0.4175 | 4.9667 | 0.4783 | 6.4967 |
| G318 | 2.5650 | 0.5123 | 8.1535 | 0.6357 | 0.6990 | 5.6651 | 18.8467 | 6.0026 | 0.6295 | 5.5963 | 0.3847 | 8.6219 |
| G319 | 2.8002 | 0.5385 | 8.2528 | 0.6117 | 0.7256 | 5.3465 | 19.6867 | 2.0878 | 0.3184 | 3.8667 | 0.4850 | 6.2467 |
| G320 | 2.6011 | 0.4736 | 6.8703 | 0.5804 | 0.6545 | 4.6906 | 14.8633 | 2.8527 | 0.4092 | 4.7107 | 0.4547 | 6.6438 |
| G321 | 2.4583 | 0.4001 | 5.2000 | 0.5177 | 0.6051 | 7.0472 | 16.1496 | 3.6628 | 0.4314 | 4.1333 | 0.4023 | 5.8376 |
| G322 | 2.6991 | 0.4626 | 6.3061 | 0.5459 | 0.6831 | 5.8950 | 18.5533 | 3.1593 | 0.4718 | 5.2370 | 0.4752 | 8.4819 |
| G323 | 3.0450 | 0.5981 | 9.3591 | 0.6253 | 0.7787 | 6.0311 | 20.3300 | 2.5865 | 0.3915 | 4.6000 | 0.4844 | 6.2204 |
| G324 | 2.9107 | 0.5411 | 8.0298 | 0.5915 | 0.7484 | 5.8159 | 18.7833 | 3.9883 | 0.5144 | 5.4333 | 0.4551 | 3.3230 |
| G325 | 2.6759 | 0.4932 | 7.2421 | 0.5859 | 0.7845 | 3.9399 | 14.0700 | 3.2394 | 0.4077 | 4.0333 | 0.4225 | 5.7507 |
| G326 | 2.3778 | 0.4192 | 5.8917 | 0.5602 | 0.5920 | 4.1696 | 12.7900 | 4.8354 | 0.5468 | 5.1963 | 0.4157 | 7.8493 |
| G327 | 2.5497 | 0.4485 | 6.2962 | 0.5601 | 0.7062 | 3.5211 | 12.2700 | 2.3415 | 0.3506 | 4.1741 | 0.4755 | 5.4467 |
| G328 | 2.9465 | 0.5979 | 9.6562 | 0.6459 | 0.7448 | 5.0543 | 16.1833 | 2.4626 | 0.3924 | 5.0000 | 0.5083 | 6.4400 |
| G329 | 2.9245 | 0.5536 | 8.3505 | 0.6013 | 0.6919 | 4.9819 | 16.2144 | 2.9990 | 0.3924 | 4.1963 | 0.4355 | 6.3137 |
| G330 | 2.9369 | 0.5636 | 8.6189 | 0.6106 | 0.7502 | 3.7828 | 17.0037 | 4.6981 | 0.5346 | 5.1074 | 0.4184 | 7.5400 |
| G331 | 2.5817 | 0.4602 | 6.5509 | 0.5677 | 0.6248 | 4.0108 | 12.7100 | 2.6693 | 0.3825 | 4.2143 | 0.4753 | 5.6115 |
| G332 | 2.7636 | 0.4973 | 7.1216 | 0.5725 | 0.7669 | 4.2000 | 15.6067 | 2.7476 | 0.4233 | 5.2143 | 0.4974 | 7.2633 |
| G333 | 2.7156 | 0.4750 | 6.6133 | 0.5572 | 0.7026 | 3.3595 | 11.8289 | 1.8720 | 0.2855 | 3.5259 | 0.4839 | 4.1452 |
| G334 | 3.0226 | 0.5554 | 8.1296 | 0.5850 | 0.7154 | 5.6565 | 18.5333 | 3.6494 | 0.5535 | 6.8667 | 0.5292 | 8.9300 |
| G335 | 2.4734 | 0.4717 | 7.1516 | 0.6072 | 0.7322 | 4.4005 | 14.5933 | 2.3002 | 0.3473 | 4.1667 | 0.4825 | 5.2133 |
| G336 | 2.8063 | 0.5139 | 7.4853 | 0.5825 | 0.7262 | 4.4214 | 15.2967 | 3.0928 | 0.4770 | 5.8667 | 0.4954 | 7.2567 |
| G337 | 2.7500 | 0.4609 | 6.1705 | 0.5338 | 0.6611 | 5.1015 | 15.1933 | 2.4464 | 0.3644 | 4.3333 | 0.4831 | 6.0833 |
| G338 | 2.5119 | 0.4557 | 6.5741 | 0.5779 | 0.6907 | 5.5251 | 18.6250 | 4.5042 | 0.5359 | 5.1417 | 0.4401 | 8.0861 |
| G339 | 2.6887 | 0.4575 | 6.1973 | 0.5416 | 0.6713 | 3.1145 | 11.3733 | 1.9654 | 0.3202 | 4.1667 | 0.5228 | 5.5233 |
| G340 | 2.8492 | 0.4925 | 6.7746 | 0.5504 | 0.7576 | 7.0886 | 18.2300 | 6.9148 | 0.7443 | 6.4667 | 0.3707 | 9.8733 |
| G341 | 2.9164 | 0.5446 | 8.0858 | 0.5951 | 0.6537 | 6.4738 | 18.9648 | 2.6424 | 0.3938 | 4.1091 | 0.4735 | 6.5536 |
| G342 | 2.7437 | 0.5100 | 7.5514 | 0.5919 | 0.7007 | 5.0646 | 17.3454 | 1.4421 | 0.2292 | 2.7833 | 0.5578 | 6.2667 |
| G343 | 2.3003 | 0.3984 | 5.4816 | 0.5514 | 0.6848 | 5.8257 | 18.6533 | 3.5336 | 0.4831 | 5.3000 | 0.4457 | 5.6370 |
| G344 | 2.6770 | 0.5016 | 7.5318 | 0.5990 | 0.6712 | 5.9395 | 18.1267 | 2.4667 | 0.3494 | 3.6833 | 0.4554 | 5.4625 |
| G345 | 2.8231 | 0.4902 | 6.7848 | 0.5524 | 0.6702 | 6.2120 | 17.3337 | 5.1248 | 0.5836 | 5.2667 | 0.3897 | 8.9885 |
| G346 | 3.1136 | 0.5774 | 8.5088 | 0.5909 | 0.6891 | 5.6313 | 18.4100 | 4.3700 | 0.5131 | 5.0000 | 0.4176 | 7.5122 |
| G347 | 2.5267 | 0.4499 | 6.3675 | 0.5674 | 0.6653 | 4.8854 | 13.2571 | 3.0571 | 0.4281 | 4.3000 | 0.4459 | 6.0233 |
| G348 | NA | NA | NA | NA | NA | NA | NA | NA | NA | NA | NA | NA |
| G349 | 2.4066 | 0.4199 | 5.8311 | 0.5559 | 0.6344 | 4.8187 | 13.8344 | 2.7794 | 0.3982 | 3.9974 | 0.4584 | 5.9619 |
| G350 | 3.0976 | 0.6080 | 9.5139 | 0.6223 | 0.8079 | 4.2652 | 18.1400 | 4.2508 | 0.5088 | 4.9370 | 0.4187 | 7.2307 |
| G351 | 2.8950 | 0.5102 | 7.1778 | 0.5605 | 0.7013 | 5.3341 | 16.3730 | 3.0507 | 0.4509 | 5.1000 | 0.4799 | 6.5696 |
| G352 | 2.8245 | 0.5129 | 7.4347 | 0.5772 | 0.7419 | 5.9298 | 18.8186 | 2.8052 | 0.4144 | 4.7833 | 0.4741 | 7.7645 |
| G353 | 2.8974 | 0.4979 | 6.8084 | 0.5460 | 0.6297 | 5.0386 | 13.5533 | 2.9444 | 0.4689 | 5.7417 | 0.5078 | 8.3167 |
| G354 | 2.7546 | 0.5134 | 7.6257 | 0.5927 | 0.7853 | 6.0273 | 19.5500 | 8.3523 | 0.8594 | 7.4333 | 0.3937 | 11.0100 |
| G355 | 3.1126 | 0.5829 | 8.6944 | 0.5961 | 0.7526 | 6.7912 | 20.9435 | 5.6131 | 0.6300 | 5.6250 | 0.3801 | 9.0849 |
| G356 | 2.6317 | 0.4570 | 6.2997 | 0.5533 | 0.6164 | 6.0007 | 15.9133 | 3.2676 | 0.4213 | 4.3333 | 0.4131 | 6.1133 |
| G357 | 2.6663 | 0.4718 | 6.6467 | 0.5629 | 0.7100 | 4.5978 | 17.9159 | 3.3567 | 0.4372 | 4.3778 | 0.4198 | 7.6500 |
| G358 | 2.3142 | 0.4022 | 5.5719 | 0.5532 | 0.6661 | 5.0848 | 13.8996 | 4.7432 | 0.5315 | 4.8667 | 0.4029 | 6.8100 |
| G359 | 2.8340 | 0.5191 | 7.5847 | 0.5826 | 0.6638 | 4.3849 | 15.1433 | 2.8457 | 0.4351 | 5.2667 | 0.4879 | 6.9767 |
| G360 | 2.7465 | 0.5178 | 7.7838 | 0.5991 | 0.6488 | 4.8150 | 13.7533 | 2.1754 | 0.3365 | 3.9750 | 0.4942 | 4.8641 |
| G361 | 2.7160 | 0.4774 | 6.6807 | 0.5600 | 0.6974 | 4.7611 | 15.2874 | 2.3323 | 0.3525 | 4.2741 | 0.4877 | 5.3596 |
| G362 | 3.0922 | 0.5902 | 8.9771 | 0.6077 | 0.6858 | 6.2364 | 18.4663 | 2.5888 | 0.3890 | 4.7095 | 0.4947 | 7.1258 |
| G363 | 2.8528 | 0.5322 | 7.8826 | 0.5938 | 0.6933 | 5.0861 | 15.8163 | 1.7860 | 0.2675 | 3.2159 | 0.4807 | 3.8157 |
| G364 | 2.9824 | 0.5390 | 7.7500 | 0.5757 | 0.7560 | 4.1509 | 14.8600 | 2.8190 | 0.4154 | 4.7667 | 0.4751 | 7.0544 |
| G365 | 3.1152 | 0.5711 | 8.3275 | 0.5835 | 0.6845 | 4.6055 | 14.9433 | 2.2160 | 0.3630 | 4.4574 | 0.5242 | 5.9380 |
| G366 | 2.6197 | 0.4930 | 7.3908 | 0.5986 | 0.7666 | 5.2802 | 17.9293 | 3.1886 | 0.4547 | 5.0704 | 0.4669 | 7.3422 |
| G367 | 3.3998 | 0.6317 | 9.3584 | 0.5909 | 0.7044 | 4.5358 | 15.4300 | 1.9369 | 0.2977 | 3.6481 | 0.4968 | 5.0811 |
| G368 | 2.6997 | 0.4684 | 6.4559 | 0.5523 | 0.7276 | 4.0854 | 14.9330 | 2.5209 | 0.3459 | 3.6963 | 0.4413 | 5.2026 |
| G369 | 2.7636 | 0.5496 | 8.6888 | 0.6313 | 0.7330 | 4.9592 | 18.3905 | 2.0692 | 0.3315 | 3.6333 | 0.5098 | 5.5456 |
| G370 | 2.7660 | 0.5099 | 7.4881 | 0.5871 | 0.7129 | 5.8674 | 17.9467 | 2.7986 | 0.4278 | 5.2000 | 0.4874 | 7.0133 |
| G371 | 3.2363 | 0.6214 | 9.5185 | 0.6078 | 0.7171 | 5.2105 | 17.5267 | 2.6993 | 0.3965 | 4.6593 | 0.4673 | 7.0033 |
| G372 | 3.2373 | 0.6152 | 9.3263 | 0.6038 | 0.7087 | 5.4022 | 17.9467 | 3.3933 | 0.4498 | 4.7333 | 0.4207 | 7.9200 |
| G373 | 3.3881 | 0.6483 | 9.8950 | 0.6094 | 0.6920 | 4.9542 | 16.8078 | 2.3202 | 0.3329 | 3.4778 | 0.4564 | 22.7256 |
| G374 | 3.0626 | 0.5906 | 9.0614 | 0.6138 | 0.7067 | 5.1356 | 16.9952 | 2.3499 | 0.3648 | 4.3815 | 0.5029 | 5.9852 |
| G375 | 2.7100 | 0.4683 | 6.4325 | 0.5498 | 0.6510 | 3.5772 | 11.6125 | 2.6816 | 0.4084 | 4.5667 | 0.4861 | 5.7833 |
| G376 | 2.7612 | 0.4795 | 6.6219 | 0.5532 | 0.6392 | 3.4921 | 10.9256 | 2.2832 | 0.3123 | 3.2667 | 0.4340 | 4.7537 |
| G377 | 2.4201 | 0.4000 | 5.2639 | 0.5273 | 0.6631 | 4.6512 | 13.7138 | 2.7650 | 0.4146 | 4.5407 | 0.4757 | 6.7281 |
| G378 | 2.5223 | 0.4242 | 5.6862 | 0.5360 | 0.7259 | 6.9521 | 18.7667 | 4.5209 | 0.5589 | 5.5593 | 0.4112 | 9.3248 |
| G379 | 2.9151 | 0.5471 | 8.1778 | 0.5975 | 0.7158 | 4.7076 | 16.7778 | 3.1135 | 0.4216 | 4.3667 | 0.4306 | 19.9096 |
| G380 | 2.2588 | 0.3729 | 4.9044 | 0.5250 | 0.6799 | 5.0980 | 15.5752 | 2.6875 | 0.3705 | 3.9417 | 0.4411 | 6.4139 |
| G381 | 2.5799 | 0.4518 | 6.3125 | 0.5570 | 0.6647 | 5.8645 | 16.7000 | 4.6400 | 0.5167 | 4.5407 | 0.3708 | 7.0963 |
| G382 | 2.4747 | 0.4988 | 8.0223 | 0.6417 | 0.7360 | 4.0161 | 14.9567 | 1.5476 | 0.2371 | 2.7222 | 0.5089 | 3.6541 |
| G383 | 2.1683 | 0.3446 | 4.3561 | 0.5061 | 0.6480 | 6.4490 | 16.9200 | 9.3572 | 0.8740 | 6.4333 | 0.3182 | 10.5533 |
| G384 | 3.0277 | 0.5878 | 9.0982 | 0.6167 | 0.7550 | 4.9678 | 16.3496 | 1.9599 | 0.3147 | 4.0000 | 0.5117 | 4.8375 |
| G385 | 2.7255 | 0.4674 | 6.3671 | 0.5462 | 0.6632 | 6.3509 | 18.1033 | 4.0386 | 0.4655 | 4.3333 | 0.3879 | 7.0400 |
| G386 | 2.5384 | 0.4553 | 6.5104 | 0.5719 | 0.7028 | 5.6159 | 18.9619 | 2.6633 | 0.3491 | 3.2667 | 0.4331 | 5.3171 |
| G387 | 2.4258 | 0.4135 | 5.5959 | 0.5422 | 0.6966 | 4.4271 | 14.9489 | 4.3170 | 0.4683 | 4.1667 | 0.3887 | 7.3289 |
| G388 | 2.5718 | 0.4738 | 6.9544 | 0.5865 | 0.6933 | 4.2354 | 13.4815 | 2.0227 | 0.3141 | 3.9296 | 0.4948 | 4.8519 |
| G389 | 2.9449 | 0.5819 | 9.1500 | 0.6291 | 0.7333 | 5.5875 | 19.9343 | 1.9124 | 0.2939 | 2.8352 | 0.5678 | 5.7398 |
| G390 | 2.7613 | 0.5188 | 7.7729 | 0.5981 | 0.7712 | 5.1105 | 15.4296 | 3.4133 | 0.4896 | 5.4333 | 0.4572 | 7.4185 |
| G391 | 2.6919 | 0.5027 | 7.4725 | 0.5935 | 0.7894 | 4.2383 | 16.5117 | 2.9240 | 0.4258 | 4.9750 | 0.4677 | 6.8450 |
| G392 | 2.4869 | 0.4656 | 6.9611 | 0.5954 | 0.6955 | 6.0561 | 19.4500 | 3.0416 | 0.4334 | 4.9667 | 0.4634 | 7.2100 |
| G393 | 2.6558 | 0.4880 | 7.1174 | 0.5849 | 0.6759 | 5.6786 | 16.7685 | 2.6546 | 0.3807 | 4.0503 | 0.4576 | 5.6903 |
| G394 | 2.6947 | 0.4689 | 6.5218 | 0.5530 | 0.6199 | 4.7792 | 13.6300 | 3.8600 | 0.4480 | 4.3000 | 0.4071 | 5.9900 |
| G395 | 2.7689 | 0.5046 | 7.3158 | 0.5796 | 0.7611 | 4.4265 | 16.9467 | 3.3536 | 0.4278 | 4.4778 | 0.4355 | 6.6707 |
| G396 | 2.6498 | 0.4764 | 6.8125 | 0.5720 | 0.6408 | 4.7340 | 14.6642 | 2.5463 | 0.3350 | 3.5333 | 0.4198 | 5.1667 |
| G397 | 2.9849 | 0.5298 | 7.5042 | 0.5644 | 0.6333 | 5.5365 | 15.0749 | 3.2222 | 0.4349 | 4.6833 | 0.4311 | 5.9486 |
| G398 | 2.8073 | 0.5151 | 7.5175 | 0.5834 | 0.6514 | 3.8472 | 13.0128 | 2.7217 | 0.3534 | 3.7048 | 0.4159 | 4.7411 |
| G399 | 2.4651 | 0.4610 | 6.8875 | 0.5876 | 0.7354 | 3.2345 | 13.8433 | 1.3162 | 0.2217 | 2.9881 | 0.5537 | 3.9469 |
| G400 | 3.0122 | 0.5715 | 8.6484 | 0.6039 | 0.7271 | 4.6996 | 17.2167 | 2.3626 | 0.3131 | 3.3667 | 0.4454 | 4.8133 |
| G401 | 2.7125 | 0.5518 | 8.9256 | 0.6476 | 0.7558 | 4.7397 | 17.4538 | 1.6783 | 0.2475 | 2.7333 | 0.4708 | 4.9267 |
| G402 | 3.0335 | 0.5559 | 8.1089 | 0.5830 | 0.7051 | 4.4661 | 16.0211 | 2.3418 | 0.3291 | 3.5704 | 0.4477 | 4.6811 |
| G403 | 3.1790 | 0.6005 | 9.0278 | 0.6012 | 0.7897 | 4.7719 | 18.0544 | 2.0963 | 0.3145 | 3.6333 | 0.4845 | 5.6637 |
| G404 | 2.8565 | 0.4975 | 6.9167 | 0.5546 | 0.6832 | 3.9030 | 14.4389 | 2.0357 | 0.3327 | 4.3667 | 0.5207 | 5.2833 |
| G405 | 3.1137 | 0.5841 | 8.7369 | 0.5969 | 0.7324 | 5.9677 | 18.8881 | 5.6650 | 0.6795 | 6.5000 | 0.3963 | 10.0250 |
| G406 | 2.8788 | 0.5147 | 7.3370 | 0.5695 | 0.6631 | 4.8892 | 16.2400 | 2.3948 | 0.3536 | 4.2667 | 0.4676 | 5.7300 |
| G407 | 3.1174 | 0.6142 | 9.6386 | 0.6277 | 0.7152 | 4.2763 | 14.6322 | 2.5378 | 0.3563 | 3.8667 | 0.4529 | 5.8148 |
| G408 | 2.8352 | 0.5431 | 8.3105 | 0.6109 | 0.7158 | 5.2290 | 15.5681 | 2.8708 | 0.4018 | 4.3000 | 0.4462 | 7.0211 |
| G409 | 2.6254 | 0.4759 | 6.8519 | 0.5770 | 0.6291 | 5.3812 | 13.8833 | 2.8046 | 0.3745 | 4.0333 | 0.4295 | 5.4667 |
| G410 | 3.0600 | 0.5771 | 8.6589 | 0.6001 | 0.7528 | 4.8279 | 18.0667 | 2.6485 | 0.3786 | 4.3667 | 0.4715 | 6.5067 |
| G411 | 3.1003 | 0.6100 | 9.5436 | 0.6248 | 0.7140 | 3.3959 | 14.1100 | 1.9618 | 0.3098 | 3.8667 | 0.5031 | 4.7500 |
| G412 | 2.7204 | 0.4711 | 6.4845 | 0.5513 | 0.6521 | 3.9606 | 13.9733 | 2.8236 | 0.3919 | 4.3333 | 0.4486 | 5.6333 |
| G413 | 2.3678 | 0.3804 | 4.8693 | 0.5106 | 0.5595 | 5.4391 | 13.9667 | 5.1807 | 0.5337 | 4.3667 | 0.3311 | 5.6100 |
| G414 | 2.6408 | 0.5061 | 7.7167 | 0.6084 | 0.7252 | 5.7876 | 18.6800 | 2.6423 | 0.3957 | 4.7333 | 0.4817 | 6.2200 |
| G415 | 2.8067 | 0.5191 | 7.6318 | 0.5888 | 0.7054 | 5.5570 | 16.3939 | 3.0289 | 0.3899 | 3.7917 | 0.4222 | 5.1450 |
| G416 | 2.6578 | 0.5042 | 7.6167 | 0.6040 | 0.7525 | 5.1588 | 17.0967 | 2.9204 | 0.3777 | 3.7667 | 0.4418 | 6.0233 |
| G417 | 3.1634 | 0.5962 | 8.9492 | 0.6002 | 0.7047 | 4.1829 | 15.2511 | 2.9773 | 0.4334 | 4.8667 | 0.4629 | 7.1052 |
| G418 | 2.7614 | 0.4901 | 6.9410 | 0.5646 | 0.7236 | 3.3115 | 12.3359 | 2.6018 | 0.3798 | 4.2333 | 0.4671 | 5.2683 |
| G419 | 2.8332 | 0.5067 | 7.2189 | 0.5691 | 0.7886 | 4.8679 | 15.9601 | 3.0251 | 0.4106 | 3.9417 | 0.4322 | 6.8129 |
| G420 | 2.8605 | 0.5569 | 8.6351 | 0.6187 | 0.7064 | 7.1374 | 20.6081 | 4.4750 | 0.5277 | 4.8000 | 0.4122 | 7.5403 |
| G421 | 2.9724 | 0.6058 | 9.8936 | 0.6439 | 0.7083 | 5.5921 | 15.1296 | 2.6355 | 0.3542 | 3.6667 | 0.4354 | 6.0091 |
| G422 | 2.4674 | 0.4750 | 7.2880 | 0.6107 | 0.6415 | 6.4157 | 17.0167 | 1.9454 | 0.3062 | 3.8815 | 0.5128 | 5.7181 |
| G423 | 2.6296 | 0.4988 | 7.5523 | 0.6059 | 0.6853 | 4.2520 | 13.9325 | 3.9206 | 0.4235 | 3.8500 | 0.3928 | 5.4933 |
| G424 | 2.8231 | 0.5737 | 9.2947 | 0.6489 | 0.7357 | 5.2007 | 19.0333 | 2.2990 | 0.3261 | 3.6481 | 0.4901 | 5.1481 |
| G425 | NA | NA | NA | NA | NA | NA | NA | NA | NA | NA | NA | NA |
| G426 | 2.6800 | 0.4922 | 7.2113 | 0.5836 | 0.7021 | 4.4081 | 15.1000 | 2.2410 | 0.3548 | 4.1667 | 0.5183 | 5.4792 |
| G427 | 2.8817 | 0.5598 | 8.7203 | 0.6186 | 0.7301 | 4.1987 | 15.0767 | 2.0801 | 0.3097 | 3.5630 | 0.4719 | 4.6493 |
| G428 | 2.7527 | 0.5458 | 8.6333 | 0.6308 | 0.7762 | 4.7501 | 16.4344 | 2.7284 | 0.3860 | 4.3519 | 0.4553 | 6.3670 |
| G429 | 2.8980 | 0.5640 | 8.7573 | 0.6199 | 0.7814 | 5.8574 | 21.1485 | 4.9370 | 0.6032 | 6.0000 | 0.4320 | 8.8667 |
| G430 | 2.4278 | 0.4957 | 8.0482 | 0.6501 | 0.7781 | 6.4832 | 22.4974 | 3.7525 | 0.5110 | 5.5667 | 0.4458 | 8.6637 |
| G431 | 2.5123 | 0.4516 | 6.4737 | 0.5745 | 0.6694 | 6.3480 | 18.5954 | 3.5984 | 0.4594 | 4.7833 | 0.4257 | 7.2422 |
| G432 | 2.7504 | 0.5504 | 8.7884 | 0.6378 | 0.6843 | 3.2192 | 14.2194 | 1.7570 | 0.2605 | 2.9630 | 0.4803 | 4.3574 |
| G433 | 2.7540 | 0.4840 | 6.7754 | 0.5603 | 0.6406 | 4.5131 | 13.8100 | 2.6574 | 0.4030 | 4.8667 | 0.4837 | 6.7367 |
| G434 | 2.8423 | 0.5018 | 7.0485 | 0.5624 | 0.6197 | 4.9127 | 14.3122 | 3.1811 | 0.4152 | 4.2444 | 0.4359 | 6.6604 |
| G435 | 2.8286 | 0.5052 | 7.1589 | 0.5691 | 0.5918 | 5.0046 | 15.7637 | 3.2587 | 0.3988 | 3.9429 | 0.4068 | 6.7619 |
| G436 | 3.0388 | 0.5688 | 8.4833 | 0.5971 | 0.7393 | 4.0922 | 14.5800 | 2.4309 | 0.3821 | 4.4167 | 0.4997 | 6.0783 |
| G437 | 2.8797 | 0.5396 | 8.1091 | 0.5960 | 0.7375 | 7.7627 | 20.6059 | 5.3329 | 0.6588 | 6.5556 | 0.4000 | 9.0656 |
| G438 | 2.8785 | 0.5357 | 7.9700 | 0.5923 | 0.6850 | 4.6536 | 15.9275 | 2.6965 | 0.4023 | 4.7917 | 0.4779 | 6.0658 |
| G439 | 2.8147 | 0.5594 | 8.8622 | 0.6320 | 0.6920 | 6.2623 | 18.1276 | 4.8788 | 0.5324 | 4.8667 | 0.4026 | 7.8567 |
| G440 | 2.9785 | 0.5251 | 7.3684 | 0.5610 | 0.5911 | 5.0651 | 14.2693 | 6.3481 | 0.6506 | 5.4519 | 0.3618 | 8.1504 |
| G441 | 3.1956 | 0.5843 | 8.5043 | 0.5822 | 0.7023 | 3.3416 | 10.7283 | 1.5909 | 0.2465 | 2.9370 | 0.4931 | 3.6809 |
| G442 | 2.6058 | 0.4969 | 7.5614 | 0.6047 | 0.7319 | 4.4668 | 14.9683 | 2.4397 | 0.3672 | 4.2000 | 0.4838 | 22.9675 |
| G443 | 2.6795 | 0.4918 | 7.2069 | 0.5840 | 0.7719 | 4.3529 | 14.9129 | 3.2383 | 0.4392 | 4.4296 | 0.4427 | 7.2339 |
| G444 | 2.8691 | 0.5319 | 7.8757 | 0.5935 | 0.6753 | 6.6793 | 18.1959 | 4.2162 | 0.5121 | 4.9667 | 0.4581 | 7.4833 |
| G445 | 2.7454 | 0.5340 | 8.3053 | 0.6211 | 0.7145 | 3.6572 | 13.9889 | 1.6501 | 0.2605 | 3.1786 | 0.5482 | 4.4131 |
| G446 | 2.9065 | 0.5199 | 7.4167 | 0.5703 | 0.6912 | 5.5426 | 15.8400 | 4.5986 | 0.5607 | 5.3667 | 0.4041 | 7.9770 |
| G447 | 2.8475 | 0.5022 | 7.0596 | 0.5618 | 0.6543 | 4.4161 | 14.1128 | 2.7495 | 0.3784 | 3.8000 | 0.4551 | 5.9133 |
| G448 | 2.8538 | 0.5139 | 7.3838 | 0.5738 | 0.6420 | 6.0833 | 16.9852 | 3.1119 | 0.3995 | 3.9815 | 0.4217 | 6.2074 |
| G449 | 2.6137 | 0.5035 | 7.7353 | 0.6132 | 0.7585 | 4.8009 | 17.1600 | 2.7852 | 0.3741 | 3.7667 | 0.4358 | 5.9962 |
| G450 | 2.5598 | 0.4635 | 6.7092 | 0.5801 | 0.6544 | 5.1333 | 14.7375 | 2.6024 | 0.3530 | 3.7167 | 0.4341 | 5.4051 |
| G451 | 2.7548 | 0.5178 | 7.7485 | 0.5989 | 0.7827 | 5.4200 | 16.4129 | 3.1760 | 0.4337 | 4.5037 | 0.4377 | 6.4053 |
| G452 | 2.8215 | 0.5455 | 8.3972 | 0.6147 | 0.6359 | 5.3346 | 16.1076 | 1.3244 | 0.2322 | 2.9667 | 0.5635 | 3.8556 |
| G453 | 2.6850 | 0.5225 | 8.1000 | 0.6177 | 0.7052 | 3.7993 | 16.5718 | 2.2395 | 0.3164 | 3.3476 | 0.4660 | 5.9942 |
| G454 | 2.8085 | 0.5230 | 7.7513 | 0.5940 | 0.7168 | 4.2681 | 15.7070 | 2.3158 | 0.3646 | 4.5889 | 0.5055 | 5.8607 |
| G455 | 3.0387 | 0.5914 | 9.1590 | 0.6201 | 0.7402 | 5.9928 | 21.4367 | 3.1270 | 0.4307 | 4.7333 | 0.4836 | 7.6467 |
| G456 | 3.0751 | 0.5727 | 8.5020 | 0.5908 | 0.7009 | 3.7949 | 14.2637 | 2.1280 | 0.3163 | 3.7704 | 0.4719 | 5.0452 |
| G457 | 2.4248 | 0.4446 | 6.4981 | 0.5819 | 0.6699 | 5.3977 | 16.2033 | 4.3382 | 0.5405 | 5.5000 | 0.4535 | 8.0667 |
| G458 | 2.7029 | 0.4935 | 7.1688 | 0.5792 | 0.7439 | 3.9066 | 13.9267 | 2.3418 | 0.3311 | 3.7333 | 0.4557 | 5.9100 |
| G459 | 2.8625 | 0.5519 | 8.4860 | 0.6140 | 0.7227 | 6.1273 | 18.4933 | 3.5603 | 0.4562 | 4.8000 | 0.4324 | 7.1167 |
| G460 | 3.1351 | 0.6107 | 9.4863 | 0.6172 | 0.7220 | 4.0779 | 15.8033 | 2.5661 | 0.3679 | 4.2333 | 0.4636 | 28.4267 |
| G461 | 2.6661 | 0.4744 | 6.7193 | 0.5657 | 0.6799 | 3.5992 | 12.0433 | 2.8931 | 0.3377 | 3.2619 | 0.4143 | 4.6057 |
| G462 | 2.6625 | 0.4955 | 7.3495 | 0.5928 | 0.7056 | 5.0864 | 15.6142 | 3.9539 | 0.4779 | 4.0667 | 0.4039 | 6.4878 |
| G463 | 3.1377 | 0.6260 | 9.9428 | 0.6349 | 0.7684 | 5.5134 | 19.1000 | 2.5430 | 0.3738 | 4.3667 | 0.4682 | 6.4000 |
| G464 | 2.9853 | 0.5547 | 8.2113 | 0.5922 | 0.5962 | 4.5407 | 13.3130 | 2.4370 | 0.3445 | 3.7333 | 0.4521 | 5.2563 |
| G465 | 2.3716 | 0.4090 | 5.6267 | 0.5489 | 0.6013 | 4.7921 | 15.1217 | 2.4782 | 0.3287 | 3.2820 | 0.4196 | 6.8690 |
| G466 | 2.7890 | 0.5118 | 7.4795 | 0.5860 | 0.6833 | 5.2402 | 14.4683 | 2.9657 | 0.3892 | 3.8333 | 0.4234 | 5.5925 |
| G467 | 2.4992 | 0.4532 | 6.5519 | 0.5782 | 0.6274 | 5.2833 | 15.0116 | 3.0788 | 0.3671 | 3.0222 | 0.4072 | 5.3556 |
| G468 | 2.9063 | 0.5155 | 7.2784 | 0.5647 | 0.6943 | 3.8039 | 12.6867 | 3.1804 | 0.4621 | 5.4000 | 0.4693 | 6.8800 |
| G469 | 2.8477 | 0.5115 | 7.3042 | 0.5721 | 0.6746 | 4.2423 | 14.1908 | 3.0178 | 0.4285 | 4.5481 | 0.4562 | 6.3080 |
|  |  |  |  |  |  |  |  |  |  |  |  |  |
|  |  |  |  |  |  |  |  |  |  |  |  |  |
|  |  |  |  |  |  |  |  |  |  |  |  |  |
|  |  |  |  |  |  |  |  |  |  |  |  |  |
| **Supplementary Table S3** Phenotypic data of all samples (wet season). | | | | | | |  |  |  |  |  |  |
| **RILs** | **CL (cm)** | **CSA（cm^2^）** | **CV（mm^3^）** | **CD（mm）** | **SSD（mm）** | **SH（cm）** | **SFW (mg)** | **RL（cm）** | **RSA（cm^2^）** | **RV（mm^3^）** | **RD（mm）** | **RFW (mg)** |
| G195 | 2.5514 | 0.4107 | 5.2661 | 0.5106 | 0.6804 | 4.3534 | 13.2583 | 2.6115 | 0.4530 | 6.2917 | 0.5498 | 6.5833 |
| G196 | 3.1133 | 0.5157 | 6.8070 | 0.5277 | 0.6888 | 5.6922 | 16.1958 | 2.7870 | 0.5155 | 7.5833 | 0.5905 | 7.3208 |
| G197 | 2.4482 | 0.4170 | 5.6685 | 0.5418 | 0.7152 | 5.8276 | 15.7708 | 3.8980 | 0.5855 | 7.0417 | 0.4861 | 8.1625 |
| G198 | 2.9360 | 0.5517 | 8.2593 | 0.5972 | 0.7105 | 4.2162 | 14.7831 | 2.7840 | 0.4248 | 5.1726 | 0.4955 | 6.3696 |
| G199 | 3.0259 | 0.5075 | 6.7833 | 0.5330 | 0.6835 | 5.4593 | 18.0292 | 2.7538 | 0.4953 | 7.1250 | 0.5749 | 6.9875 |
| G200 | 2.4150 | 0.4804 | 7.6202 | 0.6283 | 0.8181 | 5.7679 | 25.4083 | 6.3912 | 1.0790 | 14.8333 | 0.5650 | 13.4750 |
| G201 | 2.9218 | 0.5051 | 6.9693 | 0.5485 | 0.6610 | 4.3713 | 12.6667 | 2.7948 | 0.4885 | 6.8667 | 0.5584 | 6.7933 |
| G202 | 2.5589 | 0.4650 | 6.7222 | 0.5754 | 0.6091 | 4.3534 | 13.5111 | 2.7299 | 0.4499 | 5.8889 | 0.5245 | 6.1444 |
| G203 | 2.1779 | 0.3921 | 5.6347 | 0.5738 | 0.6772 | 3.4466 | 11.8111 | 2.0430 | 0.3425 | 4.5556 | 0.5349 | 5.5556 |
| G204 | 2.4942 | 0.4821 | 7.4259 | 0.6152 | 0.7536 | 5.6872 | 20.9917 | 2.9114 | 0.4706 | 6.0833 | 0.5169 | 6.7292 |
| G205 | 2.6171 | 0.4801 | 7.0221 | 0.5829 | 0.5676 | 4.9422 | 13.3750 | 2.3061 | 0.3676 | 4.6250 | 0.5090 | 4.9292 |
| G206 | 2.6299 | 0.4909 | 7.3092 | 0.5947 | 0.6577 | 3.7614 | 12.3833 | 2.2652 | 0.4041 | 5.7500 | 0.5682 | 5.8875 |
| G207 | 2.6287 | 0.4187 | 5.3189 | 0.5073 | 0.7086 | 4.9112 | 13.9250 | 3.4074 | 0.5881 | 8.0833 | 0.5498 | 6.7625 |
| G208 | 2.2133 | 0.4085 | 6.0044 | 0.5885 | 0.6388 | 4.5474 | 13.9208 | 1.8687 | 0.3194 | 4.3333 | 0.5444 | 4.8667 |
| G209 | 2.2930 | 0.3931 | 5.3718 | 0.5432 | 0.6726 | 3.5847 | 11.1750 | 1.9040 | 0.3394 | 4.8333 | 0.5697 | 6.1625 |
| G210 | 2.4153 | 0.4752 | 7.4432 | 0.6267 | 0.7158 | 4.7692 | 14.5000 | 2.3066 | 0.4371 | 6.5833 | 0.6018 | 6.7208 |
| G211 | 2.9404 | 0.5336 | 7.7155 | 0.5773 | 0.5953 | 4.5054 | 14.2333 | 2.3788 | 0.3655 | 4.5000 | 0.4909 | 6.1278 |
| G212 | 2.7130 | 0.5127 | 7.6963 | 0.6015 | 0.6282 | 4.0557 | 13.1917 | 1.7326 | 0.3001 | 4.1250 | 0.5516 | 5.4083 |
| G213 | 2.7357 | 0.5283 | 8.1222 | 0.6158 | 0.6524 | 5.0866 | 15.1952 | 2.1343 | 0.3533 | 4.7143 | 0.5279 | 5.9857 |
| G214 | 2.7305 | 0.5465 | 8.7249 | 0.6338 | 0.6918 | 3.6885 | 14.3750 | 2.2334 | 0.3927 | 5.5000 | 0.5594 | 6.1292 |
| G215 | 2.9944 | 0.5883 | 9.2278 | 0.6268 | 0.7087 | 4.6852 | 13.9500 | 2.9974 | 0.5446 | 7.9444 | 0.5806 | 7.5944 |
| G216 | 2.7773 | 0.4803 | 6.6093 | 0.5508 | 0.6266 | 4.5940 | 11.9083 | 2.7401 | 0.4162 | 5.0000 | 0.4842 | 5.9458 |
| G217 | 1.9736 | 0.3712 | 5.5758 | 0.6004 | 0.6568 | 4.4831 | 15.1333 | 2.3471 | 0.3620 | 4.4444 | 0.4933 | 5.6833 |
| G218 | 1.8473 | 0.3523 | 5.3605 | 0.6066 | 0.6796 | 4.1334 | 14.7542 | 2.1831 | 0.4132 | 6.2083 | 0.6033 | 7.6167 |
| G219 | 2.9420 | 0.5369 | 7.8129 | 0.5810 | 0.7447 | 6.6856 | 22.4625 | 3.1915 | 0.4985 | 6.1667 | 0.4967 | 6.4958 |
| G220 | 2.0527 | 0.3892 | 5.8704 | 0.6055 | 0.6733 | 4.3053 | 16.2600 | 2.1503 | 0.3871 | 5.5333 | 0.5739 | 7.3933 |
| G221 | 2.8282 | 0.5415 | 8.2649 | 0.6083 | 0.7454 | 3.3943 | 11.8250 | 1.7769 | 0.3199 | 4.6250 | 0.5876 | 5.4000 |
| G222 | 2.5614 | 0.4380 | 5.9688 | 0.5428 | 0.6780 | 5.4015 | 16.0958 | 1.5218 | 0.2953 | 4.5833 | 0.6273 | 5.6333 |
| G223 | 2.6338 | 0.4955 | 7.4123 | 0.5972 | 0.6825 | 4.5135 | 14.9583 | 2.0541 | 0.3693 | 5.2917 | 0.5789 | 5.9583 |
| G224 | 2.6136 | 0.5355 | 8.7338 | 0.6557 | 0.6543 | 4.5803 | 16.5381 | 2.9712 | 0.4939 | 6.5238 | 0.5293 | 8.6810 |
| G225 | 2.4355 | 0.4398 | 6.3125 | 0.5753 | 0.7070 | 4.4455 | 14.6542 | 1.8926 | 0.3502 | 5.2083 | 0.6039 | 6.4917 |
| G226 | 2.9234 | 0.5280 | 7.5891 | 0.5763 | 0.6557 | 5.7158 | 15.9042 | 2.5559 | 0.3908 | 4.7500 | 0.4871 | 5.7250 |
| G227 | 3.1604 | 0.6057 | 9.2745 | 0.6084 | 0.7506 | 4.3194 | 13.9571 | 1.5689 | 0.2933 | 4.3810 | 0.6033 | 5.6143 |
| G228 | 2.3445 | 0.4149 | 5.8470 | 0.5621 | 0.6903 | 4.3516 | 13.7167 | 2.1903 | 0.3941 | 5.6667 | 0.5837 | 6.6333 |
| G229 | 2.0864 | 0.3369 | 4.3451 | 0.5125 | 0.6352 | 4.1130 | 11.2667 | 2.1811 | 0.3513 | 4.5000 | 0.5210 | 5.9542 |
| G230 | 2.6560 | 0.4933 | 7.2752 | 0.5909 | 0.7705 | 4.2796 | 13.8667 | 2.2120 | 0.4088 | 6.0000 | 0.6010 | 6.1333 |
| G231 | 2.2022 | 0.3906 | 5.5463 | 0.5655 | 0.6642 | 3.8288 | 11.7708 | 1.6448 | 0.2967 | 4.2500 | 0.5769 | 6.0333 |
| G232 | 2.8197 | 0.4741 | 6.3561 | 0.5358 | 0.6821 | 2.8607 | 10.9458 | 2.1171 | 0.3573 | 4.8333 | 0.5480 | 5.9917 |
| G233 | 2.4039 | 0.4577 | 6.9325 | 0.6054 | 0.7279 | 4.3393 | 13.4042 | 1.9258 | 0.3579 | 5.2917 | 0.5983 | 6.2458 |
| G234 | 2.5075 | 0.4153 | 5.4815 | 0.5272 | 0.7589 | 3.6258 | 13.4500 | 2.7924 | 0.4532 | 5.8750 | 0.5208 | 6.3417 |
| G235 | 2.6830 | 0.5005 | 7.4357 | 0.5929 | 0.7369 | 4.8035 | 14.7750 | 2.1210 | 0.3825 | 5.4583 | 0.5749 | 6.9625 |
| G236 | 2.7897 | 0.5365 | 8.2068 | 0.6132 | 0.7309 | 4.5786 | 16.9917 | 2.1969 | 0.3956 | 5.7083 | 0.5738 | 6.8833 |
| G237 | 2.3660 | 0.4046 | 5.5316 | 0.5458 | 0.6455 | 3.9939 | 11.9042 | 1.7444 | 0.3426 | 5.4167 | 0.6293 | 6.4000 |
| G238 | 1.7961 | 0.3227 | 4.6085 | 0.5719 | 0.6969 | 5.0005 | 14.6917 | 2.2253 | 0.3924 | 5.5417 | 0.5706 | 6.3250 |
| G239 | 2.3597 | 0.4416 | 6.5859 | 0.5950 | 0.7147 | 4.7765 | 16.7319 | 1.9008 | 0.3519 | 5.2917 | 0.6006 | 6.1153 |
| G240 | 2.8898 | 0.5239 | 7.5536 | 0.5761 | 0.7494 | 4.7130 | 15.7417 | 2.0393 | 0.3829 | 5.7083 | 0.5995 | 6.8542 |
| G241 | 2.2908 | 0.3883 | 5.2572 | 0.5349 | 0.6952 | 4.9135 | 16.0583 | 2.3312 | 0.4248 | 6.2083 | 0.5807 | 7.1625 |
| G242 | 1.7683 | 0.2727 | 3.3591 | 0.4905 | 0.5894 | 4.2602 | 11.7167 | 2.1574 | 0.3512 | 4.5417 | 0.5185 | 5.8792 |
| G243 | 2.5021 | 0.4401 | 6.1692 | 0.5601 | 0.7233 | 5.4611 | 14.6583 | 2.5300 | 0.3869 | 4.7500 | 0.4956 | 5.5708 |
| G244 | 2.9014 | 0.5608 | 8.6078 | 0.6157 | 0.7025 | 4.5124 | 14.1875 | 1.7681 | 0.3265 | 4.7917 | 0.5892 | 6.0458 |
| G245 | 2.6259 | 0.4806 | 7.0108 | 0.5828 | 0.7090 | 7.8116 | 20.6042 | 5.6662 | 0.7511 | 8.0000 | 0.4340 | 8.7792 |
| G246 | 2.0740 | 0.3662 | 5.1507 | 0.5616 | 0.6407 | 4.1164 | 13.9083 | 1.9166 | 0.3379 | 4.7500 | 0.5655 | 6.1375 |
| G247 | 2.7873 | 0.5293 | 8.0061 | 0.6040 | 0.6574 | 4.1385 | 13.6458 | 1.6726 | 0.3173 | 4.8194 | 0.6047 | 5.8958 |
| G248 | 2.9909 | 0.5623 | 8.4237 | 0.5984 | 0.7294 | 4.9160 | 15.7250 | 2.1310 | 0.3705 | 5.1667 | 0.5673 | 7.2875 |
| G249 | 2.3238 | 0.4399 | 6.6299 | 0.6018 | 0.7198 | 4.3617 | 14.1583 | 2.0181 | 0.3744 | 5.5417 | 0.5903 | 6.0333 |
| G250 | 2.6761 | 0.5008 | 7.4599 | 0.5957 | 0.6709 | 3.5138 | 11.7556 | 1.6413 | 0.3047 | 4.4722 | 0.5944 | 5.5972 |
| G251 | 2.6480 | 0.4809 | 6.9673 | 0.5782 | 0.7711 | 4.5615 | 16.2042 | 2.2353 | 0.3884 | 5.3750 | 0.5604 | 6.5125 |
| G252 | 2.4745 | 0.4672 | 7.0333 | 0.6020 | 0.7590 | 4.0952 | 14.7083 | 2.6421 | 0.4303 | 5.6250 | 0.5186 | 6.4542 |
| G253 | 2.1844 | 0.4285 | 6.7060 | 0.6230 | 0.6777 | 4.2082 | 13.6042 | 1.7955 | 0.3248 | 4.6667 | 0.5758 | 28.3583 |
| G254 | 2.5293 | 0.4905 | 7.5620 | 0.6167 | 0.7209 | 3.2042 | 14.3917 | 2.2329 | 0.4082 | 5.9583 | 0.5822 | 7.4208 |
| G255 | 2.0403 | 0.3670 | 5.2386 | 0.5754 | 0.6952 | 4.4617 | 12.7708 | 1.7536 | 0.3089 | 4.3333 | 0.5608 | 5.7583 |
| G256 | 2.7775 | 0.5103 | 7.4765 | 0.5790 | 0.6679 | 3.7177 | 15.5917 | 1.8342 | 0.3069 | 4.1250 | 0.5390 | 4.9250 |
| G257 | 2.3012 | 0.3641 | 4.6035 | 0.5026 | 0.5736 | 5.4639 | 12.9919 | 2.8573 | 0.3895 | 4.2401 | 0.4345 | 6.6069 |
| G258 | 2.3177 | 0.3631 | 4.5208 | 0.4997 | 0.6203 | 4.6898 | 12.2875 | 2.7346 | 0.4210 | 5.1250 | 0.4907 | 5.8833 |
| G259 | 3.1996 | 0.5571 | 7.7291 | 0.5542 | 0.6338 | 4.5767 | 11.2611 | 2.0119 | 0.3331 | 4.4028 | 0.5270 | 5.4986 |
| G260 | 2.9397 | 0.5702 | 8.8278 | 0.6160 | 0.7303 | 3.2803 | 13.0292 | 1.9174 | 0.3339 | 4.6250 | 0.5624 | 5.5458 |
| G261 | 2.3959 | 0.4217 | 5.9148 | 0.5609 | 0.6280 | 4.4940 | 12.4958 | 1.7261 | 0.3115 | 4.4583 | 0.5774 | 5.3792 |
| G262 | 2.9330 | 0.5369 | 7.8259 | 0.5829 | 0.6729 | 4.8895 | 15.2708 | 2.2061 | 0.3714 | 5.0000 | 0.5366 | 6.8792 |
| G263 | 3.1447 | 0.6000 | 9.1259 | 0.6061 | 0.6944 | 4.8159 | 16.0833 | 2.3328 | 0.3871 | 5.1111 | 0.5306 | 6.5222 |
| G264 | 2.4368 | 0.4182 | 5.7198 | 0.5476 | 0.6435 | 4.6927 | 13.3458 | 2.3150 | 0.3720 | 4.7500 | 0.5128 | 5.6500 |
| G265 | 2.7924 | 0.5229 | 7.7981 | 0.5947 | 0.6728 | 5.0009 | 15.1857 | 1.8987 | 0.3439 | 4.9524 | 0.5764 | 7.0381 |
| G266 | 2.3238 | 0.3968 | 5.3834 | 0.5433 | 0.6314 | 5.1345 | 13.6083 | 2.4455 | 0.3931 | 5.0417 | 0.5119 | 6.3333 |
| G267 | 2.6703 | 0.4653 | 6.4534 | 0.5545 | 0.6396 | 4.4846 | 13.9583 | 2.4454 | 0.3968 | 5.1250 | 0.5165 | 6.8542 |
| G268 | 2.5916 | 0.4506 | 6.2524 | 0.5529 | 0.6178 | 4.9386 | 13.4500 | 2.5502 | 0.4054 | 5.1667 | 0.5123 | 6.6042 |
| G269 | 2.6040 | 0.5031 | 7.7529 | 0.6110 | 0.6881 | 5.7680 | 19.4208 | 2.9932 | 0.4615 | 5.7083 | 0.4922 | 7.6125 |
| G270 | 2.5523 | 0.4636 | 6.7149 | 0.5775 | 0.6979 | 4.4005 | 16.8250 | 2.9576 | 0.4695 | 5.9583 | 0.5055 | 8.3583 |
| G271 | 2.0791 | 0.3837 | 5.6472 | 0.5897 | 0.6946 | 4.8696 | 16.8375 | 2.8629 | 0.4254 | 5.0417 | 0.4740 | 7.1583 |
| G272 | 3.1365 | 0.5752 | 8.4052 | 0.5846 | 0.6984 | 6.2144 | 18.1548 | 3.2581 | 0.6313 | 9.8452 | 0.6277 | 7.6798 |
| G273 | 2.4673 | 0.3802 | 4.6681 | 0.4907 | 0.5947 | 4.5964 | 11.6375 | 1.8286 | 0.3319 | 4.8750 | 0.5976 | 5.9208 |
| G274 | 2.5705 | 0.4856 | 7.3056 | 0.6019 | 0.6786 | 4.8117 | 16.1048 | 2.1442 | 0.3372 | 4.2381 | 0.5018 | 5.4143 |
| G275 | 2.5936 | 0.4374 | 5.8893 | 0.5369 | 0.6914 | 5.5481 | 18.3250 | 2.3896 | 0.4528 | 6.8631 | 0.6046 | 6.8720 |
| G276 | 2.6798 | 0.4844 | 6.9641 | 0.5759 | 0.7383 | 4.8976 | 16.9208 | 2.2088 | 0.3490 | 4.3750 | 0.5042 | 4.8042 |
| G277 | 3.5601 | 0.6900 | 10.6374 | 0.6183 | 0.7140 | 5.0450 | 17.2000 | 2.5034 | 0.4007 | 5.1250 | 0.5090 | 7.0333 |
| G278 | 1.5702 | 0.2935 | 4.3669 | 0.5923 | 0.6569 | 5.1698 | 14.1667 | 2.5818 | 0.4466 | 6.1667 | 0.5525 | 7.1917 |
| G279 | 2.2382 | 0.4290 | 6.5431 | 0.6103 | 0.7173 | 4.3272 | 17.3167 | 2.6467 | 0.4484 | 6.0417 | 0.5397 | 8.0250 |
| G280 | 2.4227 | 0.3985 | 5.2222 | 0.5242 | 0.6073 | 3.7045 | 13.1667 | 2.7880 | 0.4327 | 5.3750 | 0.4941 | 6.5792 |
| G281 | 2.4404 | 0.3942 | 5.0794 | 0.5130 | 0.6579 | 3.7776 | 11.9125 | 1.7736 | 0.3172 | 4.5000 | 0.5728 | 5.5167 |
| G282 | 2.3733 | 0.3689 | 4.5680 | 0.4949 | 0.5676 | 4.7698 | 13.4833 | 2.5195 | 0.4159 | 5.4583 | 0.5287 | 7.0292 |
| G283 | 2.6358 | 0.4441 | 5.9503 | 0.5369 | 0.7219 | 4.5332 | 12.2792 | 2.2496 | 0.3729 | 4.9167 | 0.5311 | 5.4875 |
| G284 | 3.1152 | 0.5763 | 8.4902 | 0.5896 | 0.6966 | 4.5053 | 9.3350 | 2.0265 | 0.3641 | 5.2083 | 0.5767 | 6.3917 |
| G285 | 2.8432 | 0.5079 | 7.2379 | 0.5683 | 0.7192 | 5.9202 | 17.6292 | 2.4254 | 0.3974 | 5.2500 | 0.5241 | 6.3500 |
| G286 | 2.6398 | 0.4756 | 6.8042 | 0.5730 | 0.6338 | 4.3091 | 14.0778 | 1.9627 | 0.3312 | 4.5000 | 0.5468 | 5.0722 |
| G287 | 2.7139 | 0.4970 | 7.2445 | 0.5854 | 0.7288 | 4.4512 | 13.6708 | 1.7854 | 0.3053 | 4.1250 | 0.5445 | 4.5667 |
| G288 | 2.7943 | 0.5053 | 7.2719 | 0.5757 | 0.6857 | 4.8979 | 15.2542 | 2.1302 | 0.3804 | 5.4167 | 0.5675 | 6.3958 |
| G289 | 2.5513 | 0.4606 | 6.6136 | 0.5747 | 0.6854 | 5.1453 | 14.3000 | 2.6509 | 0.4248 | 5.4167 | 0.5192 | 7.1375 |
| G290 | 3.3864 | 0.6559 | 10.1118 | 0.6170 | 0.6622 | 5.2394 | 13.9375 | 1.9429 | 0.3340 | 4.5833 | 0.5446 | 5.6583 |
| G291 | 3.1032 | 0.5688 | 8.3111 | 0.5835 | 0.6443 | 4.4264 | 13.6458 | 2.0044 | 0.3136 | 4.0000 | 0.5146 | 5.0792 |
| G292 | 3.0116 | 0.5517 | 8.0404 | 0.5826 | 0.6979 | 3.3349 | 12.2056 | 1.8339 | 0.3172 | 4.3889 | 0.5511 | 4.8000 |
| G293 | 2.8788 | 0.5567 | 8.5490 | 0.6164 | 0.6589 | 5.1277 | 16.2333 | 2.0664 | 0.3413 | 4.4881 | 0.5283 | 6.1137 |
| G294 | 2.8521 | 0.4830 | 6.5061 | 0.5393 | 0.7036 | 4.5086 | 13.7833 | 1.9745 | 0.3418 | 4.7083 | 0.5508 | 6.1458 |
| G295 | 2.9003 | 0.5353 | 7.8682 | 0.5877 | 0.6115 | 4.1818 | 13.7000 | 1.9757 | 0.3221 | 4.1667 | 0.5193 | 5.3583 |
| G296 | 2.5932 | 0.4805 | 7.1100 | 0.5906 | 0.6653 | 4.1448 | 14.3000 | 1.9609 | 0.3381 | 4.6667 | 0.5520 | 5.9208 |
| G297 | 3.0388 | 0.5918 | 9.1895 | 0.6213 | 0.6566 | 5.0190 | 15.6708 | 2.2703 | 0.3837 | 5.2083 | 0.5481 | 23.8583 |
| G298 | 2.6815 | 0.4884 | 7.0990 | 0.5788 | 0.6835 | 4.1852 | 14.2500 | 1.7898 | 0.3229 | 4.6667 | 0.5775 | 6.1778 |
| G299 | 3.0390 | 0.5740 | 8.6412 | 0.6029 | 0.6801 | 4.4830 | 13.3833 | 1.8655 | 0.3392 | 4.8889 | 0.5792 | 5.6722 |
| G300 | 2.6229 | 0.4775 | 6.9132 | 0.5798 | 0.6289 | 4.4961 | 14.1458 | 2.0975 | 0.3570 | 4.8750 | 0.5426 | 7.0292 |
| G301 | 2.3788 | 0.3738 | 4.6627 | 0.5003 | 0.6609 | 4.5816 | 12.0000 | 2.3503 | 0.3829 | 4.9524 | 0.5186 | 6.2333 |
| G302 | 2.8080 | 0.4902 | 6.8310 | 0.5533 | 0.6666 | 4.5744 | 13.3625 | 2.0311 | 0.3329 | 4.3333 | 0.5237 | 5.4000 |
| G303 | 2.7497 | 0.4763 | 6.5835 | 0.5507 | 0.6635 | 3.1835 | 10.5000 | 1.8191 | 0.3082 | 4.2083 | 0.5531 | 5.1917 |
| G304 | 2.5131 | 0.4609 | 6.7152 | 0.5825 | 0.6347 | 3.4859 | 10.7857 | 1.9195 | 0.3079 | 3.9524 | 0.5179 | 4.6190 |
| G305 | 2.3235 | 0.4127 | 5.8348 | 0.5682 | 0.6358 | 3.1926 | 10.6095 | 1.7003 | 0.2916 | 4.0000 | 0.5532 | 5.3286 |
| G306 | 2.5102 | 0.4737 | 7.0973 | 0.6007 | 0.6817 | 3.8643 | 12.8375 | 1.8730 | 0.3100 | 4.0833 | 0.5267 | 5.5542 |
| G307 | 2.4949 | 0.4054 | 5.2492 | 0.5177 | 0.7235 | 4.6284 | 15.4917 | 1.9430 | 0.3271 | 4.3750 | 0.5361 | 5.7583 |
| G308 | 3.3700 | 0.6556 | 10.1518 | 0.6194 | 0.7076 | 4.5749 | 14.1667 | 1.7517 | 0.3144 | 4.5000 | 0.5747 | 5.5250 |
| G309 | 2.8677 | 0.5237 | 7.5931 | 0.5819 | 0.6862 | 2.8979 | 10.8000 | 1.8140 | 0.3307 | 4.8512 | 0.5831 | 4.7030 |
| G310 | 2.7467 | 0.5109 | 7.5554 | 0.5931 | 0.6393 | 3.7325 | 53.7982 | 1.7440 | 0.2914 | 3.8750 | 0.5329 | 5.5315 |
| G311 | 3.0632 | 0.5677 | 8.3920 | 0.5869 | 0.7192 | 4.4618 | 16.8958 | 2.6431 | 0.4470 | 6.0417 | 0.5395 | 8.4250 |
| G312 | 2.5158 | 0.4928 | 7.6650 | 0.6242 | 0.6257 | 4.3951 | 14.1333 | 1.5937 | 0.2667 | 3.5833 | 0.5355 | 4.6500 |
| G313 | 3.0726 | 0.5773 | 8.6422 | 0.5996 | 0.6970 | 5.2412 | 16.3417 | 2.3762 | 0.4269 | 6.1250 | 0.5712 | 7.3667 |
| G314 | 2.6044 | 0.4370 | 5.8421 | 0.5344 | 0.5704 | 4.4099 | 12.9750 | 1.7476 | 0.3013 | 4.1250 | 0.5490 | 5.2542 |
| G315 | 2.9812 | 0.5449 | 7.9181 | 0.5824 | 0.6411 | 3.3000 | 11.8708 | 2.2144 | 0.3539 | 4.5000 | 0.5115 | 5.0417 |
| G316 | 2.3562 | 0.4240 | 6.0871 | 0.5733 | 0.6842 | 4.6736 | 16.2774 | 2.4197 | 0.4107 | 5.5417 | 0.5408 | 7.2500 |
| G317 | 2.8262 | 0.5227 | 7.6941 | 0.5888 | 0.6743 | 3.5316 | 12.5417 | 1.8277 | 0.2927 | 3.7083 | 0.5103 | 5.7833 |
| G318 | 2.7610 | 0.5244 | 7.9300 | 0.6040 | 0.6939 | 3.3465 | 13.2833 | 2.1405 | 0.3639 | 4.9167 | 0.5462 | 6.4625 |
| G319 | 3.0612 | 0.6066 | 9.5644 | 0.6304 | 0.7163 | 4.8139 | 17.6125 | 2.3348 | 0.3930 | 5.2917 | 0.5379 | 7.6125 |
| G320 | 2.1553 | 0.3929 | 5.6789 | 0.5787 | 0.6925 | 5.2486 | 17.1667 | 2.7281 | 0.3975 | 4.6250 | 0.4737 | 6.1583 |
| G321 | 2.8810 | 0.4864 | 6.5341 | 0.5371 | 0.5688 | 5.6156 | 13.6875 | 2.1246 | 0.3558 | 4.7083 | 0.5308 | 5.7875 |
| G322 | 3.0929 | 0.5261 | 7.1238 | 0.5407 | 0.6579 | 6.5490 | 18.7667 | 5.2860 | 0.6661 | 6.9167 | 0.4305 | 8.3583 |
| G323 | 2.7177 | 0.5227 | 7.9929 | 0.6125 | 0.6983 | 5.4680 | 12.0413 | 2.1937 | 0.3585 | 4.6667 | 0.5220 | 6.8208 |
| G324 | 2.1220 | 0.4152 | 6.4459 | 0.6230 | 0.7089 | 3.8385 | 13.8762 | 1.8048 | 0.2899 | 3.7143 | 0.5107 | 4.6333 |
| G325 | 1.4206 | 0.2690 | 4.1094 | 0.6018 | 0.7383 | 5.3848 | 15.7375 | 2.0234 | 0.3303 | 4.3056 | 0.5232 | 5.4986 |
| G326 | 2.2003 | 0.3692 | 4.9483 | 0.5335 | 0.5792 | 3.5113 | 11.3048 | 1.9452 | 0.3373 | 4.7143 | 0.5646 | 6.4619 |
| G327 | 2.6115 | 0.4750 | 6.8852 | 0.5794 | 0.6639 | 3.8213 | 12.3208 | 2.0530 | 0.3338 | 4.2917 | 0.5189 | 5.6250 |
| G328 | 2.9574 | 0.5464 | 8.0392 | 0.5878 | 0.6591 | 4.9892 | 14.4417 | 2.6009 | 0.4139 | 5.2500 | 0.5063 | 6.9167 |
| G329 | 2.8148 | 0.5177 | 7.5952 | 0.5860 | 0.6496 | 3.9709 | 13.6917 | 1.9140 | 0.3134 | 4.0833 | 0.5238 | 5.2708 |
| G330 | 3.0440 | 0.5983 | 9.3567 | 0.6255 | 0.7077 | 4.5790 | 16.1875 | 2.5220 | 0.3778 | 4.5000 | 0.4780 | 5.8833 |
| G331 | 2.5962 | 0.4317 | 5.7185 | 0.5293 | 0.5754 | 3.7801 | 11.7458 | 2.1610 | 0.3610 | 4.8333 | 0.5319 | 6.3708 |
| G332 | 2.7750 | 0.5020 | 7.2105 | 0.5756 | 0.7195 | 4.0951 | 14.6958 | 2.1767 | 0.3639 | 4.8750 | 0.5323 | 6.3375 |
| G333 | 2.4543 | 0.4156 | 5.5870 | 0.5392 | 0.6452 | 6.1523 | 16.8500 | 2.8693 | 0.4740 | 6.2083 | 0.5251 | 7.0125 |
| G334 | 2.4557 | 0.4359 | 6.1559 | 0.5650 | 0.6885 | 4.2441 | 14.4125 | 2.6472 | 0.4181 | 5.2917 | 0.5037 | 7.2542 |
| G335 | 2.3908 | 0.4454 | 6.6121 | 0.5926 | 0.6562 | 4.0127 | 13.7083 | 2.3039 | 0.3815 | 5.0417 | 0.5283 | 6.8042 |
| G336 | 2.4932 | 0.4301 | 5.9074 | 0.5471 | 0.5986 | 3.3442 | 10.9375 | 2.1749 | 0.3572 | 4.6667 | 0.5229 | 5.7000 |
| G337 | 3.0704 | 0.5731 | 8.5044 | 0.5939 | 0.6756 | 5.8373 | 19.0500 | 2.6453 | 0.3939 | 4.6667 | 0.4764 | 6.8583 |
| G338 | 2.5879 | 0.4539 | 6.3259 | 0.5586 | 0.6345 | 4.1330 | 13.6917 | 2.2334 | 0.3684 | 4.8333 | 0.5277 | 7.1667 |
| G339 | 2.4131 | 0.3747 | 4.6370 | 0.4949 | 0.5654 | 2.7726 | 8.3000 | 1.7387 | 0.2920 | 3.8750 | 0.5341 | 4.6750 |
| G340 | 2.6190 | 0.4290 | 5.5858 | 0.5209 | 0.5671 | 4.7965 | 13.1958 | 2.2958 | 0.3487 | 4.2083 | 0.4835 | 5.5042 |
| G341 | 2.5814 | 0.4634 | 6.6074 | 0.5705 | 0.6770 | 5.2165 | 16.5583 | 2.3518 | 0.3982 | 5.3333 | 0.5383 | 6.4458 |
| G342 | 2.5399 | 0.4648 | 6.7711 | 0.5825 | 0.6844 | 5.6115 | 16.5208 | 2.4422 | 0.3903 | 4.9583 | 0.5109 | 6.3458 |
| G343 | 2.2131 | 0.3858 | 5.3567 | 0.5555 | 0.6405 | 4.4313 | 12.9292 | 2.2462 | 0.3618 | 4.6250 | 0.5140 | 6.2583 |
| G344 | 2.4494 | 0.4215 | 5.7713 | 0.5472 | 0.6660 | 5.0247 | 15.9708 | 2.3143 | 0.4116 | 5.7917 | 0.5664 | 6.9500 |
| G345 | 2.7976 | 0.4929 | 6.9130 | 0.5603 | 0.6578 | 5.4041 | 16.6875 | 2.4559 | 0.3892 | 4.9167 | 0.5130 | 6.1250 |
| G346 | 2.3020 | 0.4066 | 5.6904 | 0.5616 | 0.7237 | 5.9684 | 21.8250 | 2.8379 | 0.4866 | 6.6667 | 0.5467 | 8.3542 |
| G347 | 2.1362 | 0.3609 | 4.8263 | 0.5376 | 0.6382 | 4.3601 | 12.1542 | 2.0682 | 0.3163 | 3.8333 | 0.4867 | 4.6792 |
| G348 | 1.8492 | 0.3204 | 4.4132 | 0.5521 | 0.6368 | 5.1076 | 15.2917 | 2.5287 | 0.3614 | 4.1667 | 0.4663 | 5.6875 |
| G349 | 1.9559 | 0.3371 | 4.6189 | 0.5513 | 0.6367 | 4.4637 | 12.9048 | 2.3009 | 0.3741 | 4.8571 | 0.5194 | 6.0857 |
| G350 | 2.5891 | 0.5164 | 8.1943 | 0.6341 | 0.6258 | 4.4367 | 15.1000 | 2.5450 | 0.4169 | 5.4583 | 0.5237 | 7.6875 |
| G351 | 2.9474 | 0.5068 | 6.9370 | 0.5460 | 0.6840 | 5.6216 | 15.7000 | 2.6324 | 0.4424 | 5.9167 | 0.5395 | 7.3875 |
| G352 | 2.3134 | 0.4169 | 5.9983 | 0.5774 | 0.6564 | 4.5966 | 13.8333 | 2.2358 | 0.4111 | 6.0000 | 0.5857 | 7.2667 |
| G353 | 2.1790 | 0.3543 | 4.5878 | 0.5165 | 0.6248 | 6.2825 | 17.4167 | 4.6386 | 0.7961 | 11.0417 | 0.5702 | 9.0667 |
| G354 | 1.7365 | 0.2973 | 4.0608 | 0.5435 | 0.6647 | 4.4809 | 12.4917 | 2.1587 | 0.3768 | 5.2917 | 0.5605 | 6.8208 |
| G355 | 2.4696 | 0.4353 | 6.1037 | 0.5611 | 0.6865 | 4.8203 | 15.4792 | 2.1001 | 0.4177 | 6.5833 | 0.6333 | 7.3250 |
| G356 | 2.0641 | 0.3532 | 4.8097 | 0.5462 | 0.6328 | 5.4205 | 15.1458 | 2.5061 | 0.4031 | 5.1667 | 0.5119 | 7.1292 |
| G357 | 1.9440 | 0.3190 | 4.1603 | 0.5192 | 0.6449 | 4.5419 | 12.0542 | 2.1277 | 0.3870 | 5.5833 | 0.5782 | 6.5083 |
| G358 | 2.4055 | 0.4218 | 5.9088 | 0.5604 | 0.6745 | 5.8677 | 16.1167 | 2.8697 | 0.4814 | 6.4167 | 0.5381 | 27.8542 |
| G359 | 2.8819 | 0.5455 | 8.2248 | 0.6018 | 0.5912 | 4.2994 | 12.9500 | 1.6857 | 0.3362 | 5.3333 | 0.6360 | 26.4389 |
| G360 | 2.7770 | 0.4921 | 6.9352 | 0.5604 | 0.5536 | 4.7509 | 11.1458 | 2.0809 | 0.3718 | 5.2917 | 0.5734 | 6.0125 |
| G361 | 2.8062 | 0.5005 | 7.1175 | 0.5670 | 0.6594 | 5.2505 | 15.9125 | 2.3119 | 0.3670 | 4.6250 | 0.5053 | 4.8167 |
| G362 | 3.2572 | 0.6314 | 9.7505 | 0.6168 | 0.6914 | 5.6535 | 17.5000 | 2.7539 | 0.4950 | 7.0833 | 0.5725 | 7.3833 |
| G363 | 2.6683 | 0.4932 | 7.2611 | 0.5855 | 0.6677 | 4.4661 | 14.9708 | 2.2793 | 0.3879 | 5.2500 | 0.5447 | 5.8500 |
| G364 | 2.4369 | 0.4174 | 5.6702 | 0.5435 | 0.7067 | 5.3053 | 17.0750 | 2.8029 | 0.4463 | 5.6667 | 0.5078 | 6.5000 |
| G365 | 2.6636 | 0.4722 | 6.6754 | 0.5632 | 0.7233 | 5.5199 | 20.8000 | 2.5666 | 0.5554 | 9.6250 | 0.6935 | 7.1542 |
| G366 | 2.7607 | 0.5289 | 8.0542 | 0.6080 | 0.6873 | 5.1337 | 16.2095 | 2.7148 | 0.4428 | 5.7619 | 0.5189 | 5.8571 |
| G367 | 2.9423 | 0.5243 | 7.4386 | 0.5657 | 0.6630 | 4.9154 | 15.0750 | 2.0522 | 0.3659 | 5.2083 | 0.5695 | 6.3833 |
| G368 | 2.3090 | 0.3912 | 5.2788 | 0.5390 | 0.6405 | 5.6294 | 15.3375 | 2.2847 | 0.3627 | 4.6250 | 0.5029 | 5.2167 |
| G369 | 2.0268 | 0.4041 | 6.4077 | 0.6302 | 0.6200 | 4.6661 | 16.8083 | 1.9690 | 0.3564 | 5.1250 | 0.5760 | 6.1708 |
| G370 | 2.5501 | 0.4824 | 7.2656 | 0.6012 | 0.6622 | 6.1285 | 18.8042 | 2.7925 | 0.4462 | 5.6667 | 0.5096 | 6.3083 |
| G371 | 3.6085 | 0.7103 | 11.1421 | 0.6241 | 0.6662 | 4.2989 | 14.6750 | 2.5775 | 0.4423 | 6.0833 | 0.5488 | 5.7042 |
| G372 | 2.5981 | 0.4850 | 7.2087 | 0.5945 | 0.7640 | 5.3217 | 19.0083 | 2.4107 | 0.5518 | 10.1667 | 0.7355 | 7.1125 |
| G373 | 3.0256 | 0.5368 | 7.5880 | 0.5646 | 0.6309 | 4.2433 | 13.7958 | 2.3342 | 0.3702 | 4.7083 | 0.5042 | 5.0542 |
| G374 | 2.8757 | 0.5390 | 8.0505 | 0.5962 | 0.7331 | 5.3517 | 17.5375 | 2.6502 | 0.5096 | 7.7917 | 0.6110 | 6.9375 |
| G375 | 2.2499 | 0.3845 | 5.2462 | 0.5416 | 0.6367 | 4.8704 | 13.3778 | 2.9731 | 0.4385 | 5.1528 | 0.4694 | 5.8819 |
| G376 | 2.2275 | 0.3582 | 4.5833 | 0.5110 | 0.5752 | 3.8226 | 10.9556 | 2.6616 | 0.4050 | 4.9444 | 0.4848 | 4.4944 |
| G377 | 2.1805 | 0.3582 | 4.6803 | 0.5223 | 0.6280 | 4.5654 | 13.5167 | 2.1144 | 0.3451 | 4.4583 | 0.5199 | 4.9458 |
| G378 | 2.5456 | 0.4152 | 5.3833 | 0.5189 | 0.6515 | 4.8713 | 14.6708 | 2.6943 | 0.4273 | 5.3750 | 0.5054 | 5.7583 |
| G379 | 2.5705 | 0.4854 | 7.2955 | 0.5995 | 0.6853 | 4.8919 | 16.6292 | 2.5086 | 0.3872 | 4.7500 | 0.4912 | 4.5333 |
| G380 | 2.2676 | 0.3858 | 5.2237 | 0.5400 | 0.6728 | 5.4109 | 18.4083 | 3.1695 | 0.5131 | 6.6250 | 0.5148 | 7.3667 |
| G381 | 3.0498 | 0.5836 | 8.8743 | 0.6092 | 0.6417 | 4.9785 | 15.1833 | 2.3660 | 0.3869 | 5.0000 | 0.5203 | 6.3792 |
| G382 | 1.9605 | 0.4057 | 6.7018 | 0.6616 | 0.7348 | 4.2548 | 16.9917 | 2.3543 | 0.4084 | 5.6667 | 0.5514 | 7.0542 |
| G383 | 2.3527 | 0.3565 | 4.3074 | 0.4819 | 0.6159 | 3.6714 | 10.4429 | 2.5985 | 0.3868 | 4.5714 | 0.4736 | 5.1571 |
| G384 | 2.5788 | 0.5104 | 8.0433 | 0.6299 | 0.7740 | 3.9908 | 16.7542 | 2.6892 | 0.4300 | 5.5417 | 0.5154 | 7.3375 |
| G385 | 2.3593 | 0.4074 | 5.5812 | 0.5500 | 0.6325 | 4.4159 | 12.3500 | 1.7095 | 0.2914 | 4.0000 | 0.5440 | 5.2056 |
| G386 | 1.8673 | 0.3571 | 5.4444 | 0.6093 | 0.6328 | 4.8566 | 16.9905 | 1.8842 | 0.3105 | 4.0952 | 0.5195 | 6.1952 |
| G387 | 2.5022 | 0.4320 | 5.9338 | 0.5497 | 0.7029 | 3.6825 | 12.7042 | 2.1673 | 0.3409 | 4.2917 | 0.5001 | 5.6958 |
| G388 | 2.5102 | 0.4425 | 6.2281 | 0.5603 | 0.6324 | 2.8849 | 9.6208 | 2.5979 | 0.3971 | 4.8333 | 0.4866 | 6.2917 |
| G389 | 2.3368 | 0.4275 | 6.2339 | 0.5817 | 0.6536 | 3.6791 | 13.5611 | 2.0515 | 0.3463 | 4.6667 | 0.5385 | 6.2222 |
| G390 | 2.5517 | 0.4810 | 7.2047 | 0.6000 | 0.6969 | 4.0695 | 12.7542 | 2.0438 | 0.3424 | 4.5833 | 0.5335 | 5.9042 |
| G391 | 2.1234 | 0.4069 | 6.2105 | 0.6045 | 0.6933 | 3.6281 | 12.5458 | 2.0464 | 0.3733 | 5.4583 | 0.5809 | 6.3167 |
| G392 | 1.8913 | 0.3442 | 4.9815 | 0.5771 | 0.6954 | 4.0565 | 17.3500 | 2.3702 | 0.4145 | 5.7917 | 0.5568 | 5.9500 |
| G393 | 2.6564 | 0.4790 | 6.8833 | 0.5722 | 0.6572 | 3.6236 | 12.0542 | 2.0228 | 0.3424 | 4.6250 | 0.5390 | 5.7250 |
| G394 | 2.4463 | 0.4522 | 6.6449 | 0.5882 | 0.5996 | 3.9903 | 11.5667 | 2.0067 | 0.3211 | 4.0833 | 0.5087 | 6.1667 |
| G395 | 2.2625 | 0.4132 | 6.0032 | 0.5789 | 0.6659 | 4.8055 | 14.2292 | 2.6982 | 0.4440 | 5.8333 | 0.5273 | 7.4167 |
| G396 | 1.9163 | 0.3276 | 4.4479 | 0.5421 | 0.6339 | 4.2941 | 13.1583 | 2.3542 | 0.3964 | 5.3333 | 0.5363 | 7.1208 |
| G397 | 2.2057 | 0.3997 | 5.7685 | 0.5743 | 0.6640 | 5.1552 | 15.7030 | 2.7083 | 0.4023 | 4.7694 | 0.4743 | 7.2781 |
| G398 | 2.4500 | 0.4489 | 6.5700 | 0.5801 | 0.7049 | 4.3727 | 15.9571 | 2.6078 | 0.4245 | 5.4762 | 0.5192 | 6.8238 |
| G399 | 2.5807 | 0.4893 | 7.4069 | 0.6018 | 0.6631 | 3.3376 | 13.3292 | 2.0046 | 0.3476 | 4.7917 | 0.5518 | 5.8333 |
| G400 | 2.9560 | 0.5842 | 9.1939 | 0.6285 | 0.7115 | 4.4695 | 16.1875 | 2.3118 | 0.4154 | 5.9583 | 0.5735 | 6.4042 |
| G401 | 2.6659 | 0.5334 | 8.4973 | 0.6365 | 0.7831 | 4.9479 | 18.8625 | 1.8181 | 0.3690 | 6.0417 | 0.6557 | 7.9000 |
| G402 | 3.0779 | 0.5783 | 8.6491 | 0.5985 | 0.6836 | 5.1806 | 15.3785 | 2.6872 | 0.4066 | 4.9167 | 0.4776 | 6.6423 |
| G403 | 2.8412 | 0.5399 | 8.1930 | 0.6037 | 0.7369 | 5.8308 | 20.3833 | 2.4601 | 0.4277 | 5.9583 | 0.5541 | 8.2000 |
| G404 | 2.6228 | 0.4327 | 5.6912 | 0.5239 | 0.6220 | 4.9002 | 14.2000 | 1.9946 | 0.3797 | 5.7500 | 0.6061 | 7.2542 |
| G405 | 2.0663 | 0.3748 | 5.4111 | 0.5700 | 0.5648 | 5.1975 | 15.4000 | 2.4721 | 0.4229 | 5.7917 | 0.5465 | 7.0625 |
| G406 | 2.9311 | 0.5387 | 7.8824 | 0.5843 | 0.6703 | 4.7693 | 16.7292 | 2.0696 | 0.4100 | 6.5000 | 0.6315 | 7.8250 |
| G407 | 2.7957 | 0.5237 | 7.8342 | 0.5970 | 0.6879 | 5.7144 | 18.1042 | 3.7975 | 0.5906 | 7.3333 | 0.4946 | 8.9292 |
| G408 | 2.7138 | 0.4945 | 7.1744 | 0.5787 | 0.6784 | 5.4055 | 16.0417 | 2.6216 | 0.4460 | 6.0833 | 0.5447 | 7.8458 |
| G409 | 2.2275 | 0.3766 | 5.0821 | 0.5369 | 0.6380 | 4.4308 | 11.7750 | 1.8571 | 0.3248 | 4.5417 | 0.5624 | 5.5083 |
| G410 | 2.3291 | 0.4036 | 5.5777 | 0.5501 | 0.6546 | 3.8046 | 12.0444 | 1.6798 | 0.3318 | 5.2222 | 0.6337 | 6.1944 |
| G411 | 2.3055 | 0.4410 | 6.7192 | 0.6035 | 0.7057 | 4.3531 | 17.3625 | 2.5313 | 0.4326 | 5.9583 | 0.5504 | 36.3274 |
| G412 | 2.3934 | 0.4027 | 5.4129 | 0.5351 | 0.6231 | 4.7967 | 16.6417 | 2.3980 | 0.4147 | 5.7083 | 0.5506 | 6.9875 |
| G413 | 2.1301 | 0.3272 | 3.9943 | 0.4839 | 0.5109 | 4.6246 | 11.3958 | 2.5308 | 0.3948 | 4.8750 | 0.4977 | 5.3250 |
| G414 | 2.2951 | 0.4125 | 5.9011 | 0.5721 | 0.6313 | 5.2413 | 16.7542 | 2.1679 | 0.3844 | 5.4583 | 0.5669 | 7.5333 |
| G415 | 2.5981 | 0.4657 | 6.6441 | 0.5701 | 0.5912 | 5.0227 | 13.2000 | 2.8688 | 0.4603 | 5.9167 | 0.5140 | 6.9917 |
| G416 | 2.1079 | 0.3878 | 5.6959 | 0.5813 | 0.7327 | 4.4742 | 14.9875 | 1.9074 | 0.3662 | 5.5833 | 0.6126 | 6.9583 |
| G417 | 2.3864 | 0.4060 | 5.4922 | 0.5401 | 0.7223 | 4.6258 | 15.6083 | 2.3794 | 0.4596 | 7.0833 | 0.6159 | 8.4208 |
| G418 | 2.2983 | 0.4100 | 5.8264 | 0.5654 | 0.5882 | 3.8693 | 10.9125 | 2.2893 | 0.4167 | 6.0417 | 0.5832 | 7.3292 |
| G419 | 2.2797 | 0.3942 | 5.4275 | 0.5436 | 0.6268 | 4.4093 | 14.0556 | 2.5817 | 0.4444 | 6.0556 | 0.5466 | 6.7611 |
| G420 | 2.5756 | 0.5015 | 7.7846 | 0.6195 | 0.7099 | 6.1120 | 18.5625 | 3.0616 | 0.5220 | 7.0833 | 0.5449 | 8.8042 |
| G421 | 2.3105 | 0.4178 | 6.0026 | 0.5746 | 0.6118 | 5.1620 | 13.6167 | 2.1240 | 0.3666 | 5.0417 | 0.5494 | 6.4958 |
| G422 | 2.2568 | 0.4067 | 5.8384 | 0.5707 | 0.6951 | 6.0223 | 17.7583 | 2.6340 | 0.3955 | 4.7083 | 0.4775 | 6.1750 |
| G423 | 2.8492 | 0.5153 | 7.4374 | 0.5750 | 0.6349 | 4.3706 | 13.2905 | 2.0772 | 0.3723 | 5.2857 | 0.5711 | 6.9857 |
| G424 | 2.7113 | 0.5219 | 8.0000 | 0.6121 | 0.6331 | 3.8732 | 12.2542 | 2.0705 | 0.3643 | 5.1250 | 0.5601 | 6.2833 |
| G425 | 2.3895 | 0.4289 | 6.1187 | 0.5712 | 0.6671 | 4.5518 | 13.2056 | 2.3329 | 0.3955 | 5.3333 | 0.5402 | 5.6778 |
| G426 | 2.6816 | 0.4842 | 6.9614 | 0.5744 | 0.6885 | 4.7236 | 13.8750 | 2.1436 | 0.3990 | 5.9167 | 0.5925 | 7.3875 |
| G427 | 2.9097 | 0.5164 | 7.2833 | 0.5657 | 0.6660 | 5.3188 | 16.9458 | 2.6265 | 0.4263 | 5.5417 | 0.5193 | 7.5542 |
| G428 | 2.7229 | 0.4964 | 7.1972 | 0.5803 | 0.7130 | 4.6668 | 14.7750 | 2.1848 | 0.4202 | 6.4583 | 0.6128 | 7.6875 |
| G429 | 2.6788 | 0.5276 | 8.2691 | 0.6273 | 0.7220 | 3.9809 | 15.2389 | 1.9737 | 0.3662 | 5.4444 | 0.5950 | 7.5889 |
| G430 | 2.6443 | 0.5243 | 8.3009 | 0.6308 | 0.6558 | 4.9758 | 15.2810 | 2.0774 | 0.3666 | 5.1429 | 0.5615 | 6.6095 |
| G431 | 2.7075 | 0.4904 | 7.0575 | 0.5767 | 0.6818 | 5.7518 | 15.3500 | 2.8409 | 0.4614 | 5.9583 | 0.5183 | 7.0792 |
| G432 | 2.8212 | 0.5549 | 8.6829 | 0.6252 | 0.7333 | 6.1248 | 19.7083 | 2.5837 | 0.5750 | 10.2083 | 0.7094 | 8.2708 |
| G433 | 2.4770 | 0.4341 | 6.0574 | 0.5575 | 0.7090 | 6.0860 | 20.1875 | 2.0715 | 0.3873 | 5.7500 | 0.5964 | 7.1708 |
| G434 | 2.4716 | 0.4340 | 6.0778 | 0.5585 | 0.6471 | 5.7514 | 16.2167 | 2.7292 | 0.4422 | 5.7083 | 0.5155 | 7.6792 |
| G435 | 2.5157 | 0.4286 | 5.8048 | 0.5430 | 0.6443 | 4.7216 | 12.9250 | 2.4228 | 0.3980 | 5.2083 | 0.5234 | 7.1542 |
| G436 | 2.5409 | 0.4171 | 5.4546 | 0.5225 | 0.7410 | 5.7618 | 18.4389 | 2.8048 | 0.4710 | 6.3333 | 0.5327 | 6.7333 |
| G437 | 2.8692 | 0.4701 | 6.1342 | 0.5214 | 0.6697 | 5.4722 | 15.4667 | 2.7386 | 0.6085 | 10.7917 | 0.7073 | 7.1042 |
| G438 | 2.9369 | 0.5195 | 7.3122 | 0.5632 | 0.6283 | 5.3303 | 13.6208 | 3.6449 | 0.6535 | 9.5000 | 0.5486 | 5.9625 |
| G439 | 2.6528 | 0.4913 | 7.2292 | 0.5892 | 0.7020 | 5.6304 | 18.2750 | 3.9035 | 0.5673 | 6.5833 | 0.4625 | 8.2458 |
| G440 | 2.9852 | 0.4969 | 6.5889 | 0.5304 | 0.7005 | 5.3180 | 14.9500 | 4.0186 | 0.5431 | 5.8333 | 0.4292 | 7.4667 |
| G441 | 2.5989 | 0.4703 | 6.7597 | 0.5759 | 0.5689 | 4.9344 | 13.3667 | 3.4808 | 0.5049 | 5.8750 | 0.4625 | 6.8208 |
| G442 | 1.9895 | 0.3493 | 4.8839 | 0.5606 | 0.6454 | 4.3388 | 13.2708 | 2.8127 | 0.4588 | 5.9583 | 0.5198 | 6.3000 |
| G443 | 2.3267 | 0.4025 | 5.5316 | 0.5505 | 0.7149 | 4.8351 | 13.5042 | 3.4848 | 0.5531 | 7.0417 | 0.5080 | 6.1292 |
| G444 | 2.8177 | 0.4996 | 7.0540 | 0.5647 | 0.5714 | 4.8045 | 14.2792 | 2.9051 | 0.4614 | 5.8333 | 0.5061 | 6.3375 |
| G445 | 1.9071 | 0.3548 | 5.2579 | 0.5876 | 0.5868 | 5.5739 | 15.7875 | 3.3288 | 0.5143 | 6.3333 | 0.4927 | 6.9208 |
| G446 | 2.2668 | 0.3941 | 5.4560 | 0.5518 | 0.6698 | 4.9005 | 13.6500 | 3.0925 | 0.4846 | 6.0278 | 0.4994 | 6.1778 |
| G447 | 2.4383 | 0.4136 | 5.6000 | 0.5395 | 0.5659 | 5.4236 | 14.7167 | 2.4289 | 0.3688 | 4.4583 | 0.4842 | 5.5083 |
| G448 | 2.2082 | 0.3762 | 5.1122 | 0.5436 | 0.6306 | 4.8637 | 13.0792 | 2.3270 | 0.3691 | 4.6667 | 0.5054 | 5.8208 |
| G449 | 2.5408 | 0.4704 | 6.9360 | 0.5883 | 0.6129 | 4.7723 | 12.9083 | 2.3032 | 0.3683 | 4.7083 | 0.5109 | 4.9458 |
| G450 | 2.6721 | 0.4366 | 5.6823 | 0.5199 | 0.6686 | 5.1901 | 16.1667 | 3.3513 | 0.5477 | 7.1667 | 0.5226 | 6.5250 |
| G451 | 2.1147 | 0.3721 | 5.2004 | 0.5600 | 0.6259 | 5.0410 | 14.7167 | 2.9785 | 0.4737 | 6.0000 | 0.5079 | 6.5042 |
| G452 | 2.7189 | 0.4703 | 6.4700 | 0.5509 | 0.6379 | 5.7381 | 18.4208 | 2.6763 | 0.4584 | 6.2500 | 0.5460 | 6.9708 |
| G453 | 2.6855 | 0.4818 | 6.8902 | 0.5715 | 0.6172 | 4.2014 | 12.4542 | 2.8270 | 0.3968 | 4.4583 | 0.4517 | 5.0125 |
| G454 | 2.9541 | 0.5432 | 7.9468 | 0.5852 | 0.6598 | 4.0625 | 14.8500 | 2.5370 | 0.4542 | 6.4583 | 0.5703 | 6.1417 |
| G455 | 2.6405 | 0.4862 | 7.1333 | 0.5869 | 0.6523 | 4.7263 | 16.9250 | 2.4421 | 0.4149 | 5.6250 | 0.5410 | 7.3000 |
| G456 | 2.9794 | 0.5111 | 6.9833 | 0.5465 | 0.6660 | 6.8336 | 21.1958 | 3.9295 | 0.6854 | 9.6250 | 0.5721 | 7.3083 |
| G457 | 2.5964 | 0.4570 | 6.4044 | 0.5603 | 0.5665 | 4.5227 | 13.8750 | 2.8596 | 0.4539 | 5.7500 | 0.5064 | 6.9708 |
| G458 | 2.8642 | 0.5134 | 7.3138 | 0.5707 | 0.6324 | 4.8621 | 16.3208 | 2.9233 | 0.4960 | 6.7083 | 0.5400 | 6.7500 |
| G459 | 2.6400 | 0.4738 | 6.7570 | 0.5715 | 0.6602 | 4.6123 | 13.3792 | 2.5344 | 0.3883 | 4.7083 | 0.4872 | 4.8875 |
| G460 | 2.8921 | 0.5608 | 8.6623 | 0.6174 | 0.6859 | 3.7988 | 17.1583 | 2.8404 | 0.4718 | 6.2500 | 0.5284 | 8.2292 |
| G461 | 2.3294 | 0.3787 | 4.9058 | 0.5174 | 0.6501 | 4.2796 | 13.8042 | 1.9562 | 0.3073 | 3.8750 | 0.5007 | 4.5875 |
| G462 | 1.9073 | 0.3451 | 4.9646 | 0.5760 | 0.7423 | 5.3119 | 17.4583 | 2.7282 | 0.4307 | 5.4167 | 0.5015 | 5.9750 |
| G463 | 2.6877 | 0.5159 | 7.9129 | 0.6143 | 0.7530 | 5.8325 | 19.9875 | 3.0527 | 0.5232 | 7.1667 | 0.5560 | 7.2458 |
| G464 | 2.8890 | 0.5105 | 7.1759 | 0.5621 | 0.6245 | 6.6857 | 18.5417 | 3.5806 | 0.6135 | 8.4583 | 0.5512 | 7.7417 |
| G465 | 1.7818 | 0.3155 | 4.4524 | 0.5691 | 0.6351 | 4.3692 | 14.2583 | 2.6706 | 0.4317 | 5.5417 | 0.5150 | 7.2833 |
| G466 | 2.7790 | 0.4737 | 6.4575 | 0.5419 | 0.6951 | 4.6616 | 15.7583 | 2.5473 | 0.4469 | 6.2500 | 0.5638 | 6.2000 |
| G467 | 2.0723 | 0.3330 | 4.2462 | 0.5109 | 0.5479 | 4.3715 | 12.6833 | 2.3808 | 0.3654 | 4.4583 | 0.4888 | 5.3292 |
| G468 | 2.3781 | 0.4245 | 6.0404 | 0.5675 | 0.6257 | 5.3080 | 15.7583 | 3.6323 | 0.5752 | 7.3333 | 0.5155 | 7.0958 |
| G469 | 2.7635 | 0.4903 | 6.9368 | 0.5646 | 0.6534 | 3.7680 | 13.5000 | 3.3615 | 0.5560 | 7.3750 | 0.5284 | 7.5667 |
|  |  |  |  |  |  |  |  |  |  |  |  |  |
|  |  |  |  |  |  |  |  |  |  |  |  |  |
|  |  |  |  |  |  |  |  |  |  |  |  |  |
|  |  |  |  |  |  |  |  |  |  |  |  |  |
| **Supplementary Table S3** Heading date. | | | |  |  |  |  |  |  |  |  |  |
| **RILs** | Heading date (d) |  |  |  |  |  |  |  |  |  |  |  |
| G195 | 96.54 |  |  |  |  |  |  |  |  |  |  |  |
| G196 | 98.73 |  |  |  |  |  |  |  |  |  |  |  |
| G197 | 98.36 |  |  |  |  |  |  |  |  |  |  |  |
| G198 | 95.33 |  |  |  |  |  |  |  |  |  |  |  |
| G199 | 100.30 |  |  |  |  |  |  |  |  |  |  |  |
| G200 | 91.50 |  |  |  |  |  |  |  |  |  |  |  |
| G201 | 99.82 |  |  |  |  |  |  |  |  |  |  |  |
| G202 | 99.10 |  |  |  |  |  |  |  |  |  |  |  |
| G203 | 102.53 |  |  |  |  |  |  |  |  |  |  |  |
| G204 | 88.80 |  |  |  |  |  |  |  |  |  |  |  |
| G205 | 106.25 |  |  |  |  |  |  |  |  |  |  |  |
| G206 | 99.80 |  |  |  |  |  |  |  |  |  |  |  |
| G207 | 98.94 |  |  |  |  |  |  |  |  |  |  |  |
| G208 | 107.58 |  |  |  |  |  |  |  |  |  |  |  |
| G209 | 111.67 |  |  |  |  |  |  |  |  |  |  |  |
| G210 | 103.75 |  |  |  |  |  |  |  |  |  |  |  |
| G211 | 108.67 |  |  |  |  |  |  |  |  |  |  |  |
| G212 | 110.83 |  |  |  |  |  |  |  |  |  |  |  |
| G213 | 100.73 |  |  |  |  |  |  |  |  |  |  |  |
| G214 | 97.64 |  |  |  |  |  |  |  |  |  |  |  |
| G215 | 100.06 |  |  |  |  |  |  |  |  |  |  |  |
| G216 | 94.73 |  |  |  |  |  |  |  |  |  |  |  |
| G217 | 104.46 |  |  |  |  |  |  |  |  |  |  |  |
| G218 | 101.83 |  |  |  |  |  |  |  |  |  |  |  |
| G219 | 88.77 |  |  |  |  |  |  |  |  |  |  |  |
| G220 | 96.58 |  |  |  |  |  |  |  |  |  |  |  |
| G221 | 92.64 |  |  |  |  |  |  |  |  |  |  |  |
| G222 | 90.08 |  |  |  |  |  |  |  |  |  |  |  |
| G223 | 92.58 |  |  |  |  |  |  |  |  |  |  |  |
| G224 | 100.15 |  |  |  |  |  |  |  |  |  |  |  |
| G225 | 102.70 |  |  |  |  |  |  |  |  |  |  |  |
| G226 | 101.73 |  |  |  |  |  |  |  |  |  |  |  |
| G227 | 98.82 |  |  |  |  |  |  |  |  |  |  |  |
| G228 | 92.45 |  |  |  |  |  |  |  |  |  |  |  |
| G229 | 90.00 |  |  |  |  |  |  |  |  |  |  |  |
| G230 | 97.23 |  |  |  |  |  |  |  |  |  |  |  |
| G231 | 104.77 |  |  |  |  |  |  |  |  |  |  |  |
| G232 | 100.71 |  |  |  |  |  |  |  |  |  |  |  |
| G233 | 94.80 |  |  |  |  |  |  |  |  |  |  |  |
| G234 | 96.46 |  |  |  |  |  |  |  |  |  |  |  |
| G235 | 95.57 |  |  |  |  |  |  |  |  |  |  |  |
| G236 | 91.18 |  |  |  |  |  |  |  |  |  |  |  |
| G237 | 96.45 |  |  |  |  |  |  |  |  |  |  |  |
| G238 | 101.82 |  |  |  |  |  |  |  |  |  |  |  |
| G239 | 100.81 |  |  |  |  |  |  |  |  |  |  |  |
| G240 | 93.08 |  |  |  |  |  |  |  |  |  |  |  |
| G241 | 96.27 |  |  |  |  |  |  |  |  |  |  |  |
| G242 | 110.64 |  |  |  |  |  |  |  |  |  |  |  |
| G243 | 98.60 |  |  |  |  |  |  |  |  |  |  |  |
| G244 | 91.00 |  |  |  |  |  |  |  |  |  |  |  |
| G245 | 89.58 |  |  |  |  |  |  |  |  |  |  |  |
| G246 | 97.08 |  |  |  |  |  |  |  |  |  |  |  |
| G247 | 96.50 |  |  |  |  |  |  |  |  |  |  |  |
| G248 | 97.36 |  |  |  |  |  |  |  |  |  |  |  |
| G249 | 99.27 |  |  |  |  |  |  |  |  |  |  |  |
| G250 | 87.43 |  |  |  |  |  |  |  |  |  |  |  |
| G251 | 99.90 |  |  |  |  |  |  |  |  |  |  |  |
| G252 | 101.38 |  |  |  |  |  |  |  |  |  |  |  |
| G253 | 100.90 |  |  |  |  |  |  |  |  |  |  |  |
| G254 | 93.42 |  |  |  |  |  |  |  |  |  |  |  |
| G255 | 98.00 |  |  |  |  |  |  |  |  |  |  |  |
| G256 | 101.60 |  |  |  |  |  |  |  |  |  |  |  |
| G257 | 98.94 |  |  |  |  |  |  |  |  |  |  |  |
| G258 | 98.46 |  |  |  |  |  |  |  |  |  |  |  |
| G259 | 97.11 |  |  |  |  |  |  |  |  |  |  |  |
| G260 | 93.00 |  |  |  |  |  |  |  |  |  |  |  |
| G261 | 104.10 |  |  |  |  |  |  |  |  |  |  |  |
| G262 | 100.00 |  |  |  |  |  |  |  |  |  |  |  |
| G263 | 89.21 |  |  |  |  |  |  |  |  |  |  |  |
| G264 | 105.08 |  |  |  |  |  |  |  |  |  |  |  |
| G265 | 99.60 |  |  |  |  |  |  |  |  |  |  |  |
| G266 | 113.85 |  |  |  |  |  |  |  |  |  |  |  |
| G267 | 106.43 |  |  |  |  |  |  |  |  |  |  |  |
| G268 | 89.31 |  |  |  |  |  |  |  |  |  |  |  |
| G269 | 95.79 |  |  |  |  |  |  |  |  |  |  |  |
| G270 | 91.00 |  |  |  |  |  |  |  |  |  |  |  |
| G271 | 99.25 |  |  |  |  |  |  |  |  |  |  |  |
| G272 | 95.54 |  |  |  |  |  |  |  |  |  |  |  |
| G273 | 100.29 |  |  |  |  |  |  |  |  |  |  |  |
| G274 | 100.09 |  |  |  |  |  |  |  |  |  |  |  |
| G275 | 94.56 |  |  |  |  |  |  |  |  |  |  |  |
| G276 | 100.08 |  |  |  |  |  |  |  |  |  |  |  |
| G277 | 93.14 |  |  |  |  |  |  |  |  |  |  |  |
| G278 | 109.55 |  |  |  |  |  |  |  |  |  |  |  |
| G279 | 100.44 |  |  |  |  |  |  |  |  |  |  |  |
| G280 | 100.47 |  |  |  |  |  |  |  |  |  |  |  |
| G281 | 98.08 |  |  |  |  |  |  |  |  |  |  |  |
| G282 | 100.75 |  |  |  |  |  |  |  |  |  |  |  |
| G283 | 101.79 |  |  |  |  |  |  |  |  |  |  |  |
| G284 | 96.10 |  |  |  |  |  |  |  |  |  |  |  |
| G285 | 106.10 |  |  |  |  |  |  |  |  |  |  |  |
| G286 | 100.62 |  |  |  |  |  |  |  |  |  |  |  |
| G287 | 89.20 |  |  |  |  |  |  |  |  |  |  |  |
| G288 | 111.40 |  |  |  |  |  |  |  |  |  |  |  |
| G289 | 98.14 |  |  |  |  |  |  |  |  |  |  |  |
| G290 | 95.55 |  |  |  |  |  |  |  |  |  |  |  |
| G291 | 100.15 |  |  |  |  |  |  |  |  |  |  |  |
| G292 | 95.00 |  |  |  |  |  |  |  |  |  |  |  |
| G293 | 100.86 |  |  |  |  |  |  |  |  |  |  |  |
| G294 | 97.19 |  |  |  |  |  |  |  |  |  |  |  |
| G295 | 93.07 |  |  |  |  |  |  |  |  |  |  |  |
| G296 | 106.08 |  |  |  |  |  |  |  |  |  |  |  |
| G297 | 106.91 |  |  |  |  |  |  |  |  |  |  |  |
| G298 | 103.00 |  |  |  |  |  |  |  |  |  |  |  |
| G299 | 108.00 |  |  |  |  |  |  |  |  |  |  |  |
| G300 | 110.77 |  |  |  |  |  |  |  |  |  |  |  |
| G301 | 102.50 |  |  |  |  |  |  |  |  |  |  |  |
| G302 | 100.33 |  |  |  |  |  |  |  |  |  |  |  |
| G303 | 96.25 |  |  |  |  |  |  |  |  |  |  |  |
| G304 | 102.23 |  |  |  |  |  |  |  |  |  |  |  |
| G305 | 98.30 |  |  |  |  |  |  |  |  |  |  |  |
| G306 | 94.93 |  |  |  |  |  |  |  |  |  |  |  |
| G307 | 110.00 |  |  |  |  |  |  |  |  |  |  |  |
| G308 | 97.93 |  |  |  |  |  |  |  |  |  |  |  |
| G309 | 107.82 |  |  |  |  |  |  |  |  |  |  |  |
| G310 | 99.94 |  |  |  |  |  |  |  |  |  |  |  |
| G311 | 102.64 |  |  |  |  |  |  |  |  |  |  |  |
| G312 | 102.25 |  |  |  |  |  |  |  |  |  |  |  |
| G313 | 93.83 |  |  |  |  |  |  |  |  |  |  |  |
| G314 | 103.86 |  |  |  |  |  |  |  |  |  |  |  |
| G315 | 97.71 |  |  |  |  |  |  |  |  |  |  |  |
| G316 | 104.90 |  |  |  |  |  |  |  |  |  |  |  |
| G317 | 102.58 |  |  |  |  |  |  |  |  |  |  |  |
| G318 | 93.07 |  |  |  |  |  |  |  |  |  |  |  |
| G319 | 96.55 |  |  |  |  |  |  |  |  |  |  |  |
| G320 | 99.42 |  |  |  |  |  |  |  |  |  |  |  |
| G321 | 107.30 |  |  |  |  |  |  |  |  |  |  |  |
| G322 | 95.20 |  |  |  |  |  |  |  |  |  |  |  |
| G323 | 96.64 |  |  |  |  |  |  |  |  |  |  |  |
| G324 | 97.81 |  |  |  |  |  |  |  |  |  |  |  |
| G325 | 107.54 |  |  |  |  |  |  |  |  |  |  |  |
| G326 | 99.25 |  |  |  |  |  |  |  |  |  |  |  |
| G327 | 106.25 |  |  |  |  |  |  |  |  |  |  |  |
| G328 | 100.46 |  |  |  |  |  |  |  |  |  |  |  |
| G329 | 92.29 |  |  |  |  |  |  |  |  |  |  |  |
| G330 | 93.67 |  |  |  |  |  |  |  |  |  |  |  |
| G331 | 98.15 |  |  |  |  |  |  |  |  |  |  |  |
| G332 | 107.64 |  |  |  |  |  |  |  |  |  |  |  |
| G333 | 90.21 |  |  |  |  |  |  |  |  |  |  |  |
| G334 | 99.92 |  |  |  |  |  |  |  |  |  |  |  |
| G335 | 100.91 |  |  |  |  |  |  |  |  |  |  |  |
| G336 | 103.54 |  |  |  |  |  |  |  |  |  |  |  |
| G337 | 95.40 |  |  |  |  |  |  |  |  |  |  |  |
| G338 | 99.50 |  |  |  |  |  |  |  |  |  |  |  |
| G339 | 102.56 |  |  |  |  |  |  |  |  |  |  |  |
| G340 | 109.82 |  |  |  |  |  |  |  |  |  |  |  |
| G341 | 94.87 |  |  |  |  |  |  |  |  |  |  |  |
| G342 | 98.12 |  |  |  |  |  |  |  |  |  |  |  |
| G343 | 99.67 |  |  |  |  |  |  |  |  |  |  |  |
| G344 | 96.80 |  |  |  |  |  |  |  |  |  |  |  |
| G345 | 99.44 |  |  |  |  |  |  |  |  |  |  |  |
| G346 | 87.93 |  |  |  |  |  |  |  |  |  |  |  |
| G347 | 99.27 |  |  |  |  |  |  |  |  |  |  |  |
| G348 | 99.56 |  |  |  |  |  |  |  |  |  |  |  |
| G349 | 104.80 |  |  |  |  |  |  |  |  |  |  |  |
| G350 | 97.46 |  |  |  |  |  |  |  |  |  |  |  |
| G351 | 105.43 |  |  |  |  |  |  |  |  |  |  |  |
| G352 | 95.94 |  |  |  |  |  |  |  |  |  |  |  |
| G353 | 104.25 |  |  |  |  |  |  |  |  |  |  |  |
| G354 | 109.23 |  |  |  |  |  |  |  |  |  |  |  |
| G355 | 100.18 |  |  |  |  |  |  |  |  |  |  |  |
| G356 | 110.73 |  |  |  |  |  |  |  |  |  |  |  |
| G357 | 92.10 |  |  |  |  |  |  |  |  |  |  |  |
| G358 | 92.13 |  |  |  |  |  |  |  |  |  |  |  |
| G359 | 99.08 |  |  |  |  |  |  |  |  |  |  |  |
| G360 | 100.25 |  |  |  |  |  |  |  |  |  |  |  |
| G361 | 91.33 |  |  |  |  |  |  |  |  |  |  |  |
| G362 | 95.27 |  |  |  |  |  |  |  |  |  |  |  |
| G363 | 94.75 |  |  |  |  |  |  |  |  |  |  |  |
| G364 | 97.33 |  |  |  |  |  |  |  |  |  |  |  |
| G365 | 99.08 |  |  |  |  |  |  |  |  |  |  |  |
| G366 | 101.44 |  |  |  |  |  |  |  |  |  |  |  |
| G367 | 98.30 |  |  |  |  |  |  |  |  |  |  |  |
| G368 | 105.67 |  |  |  |  |  |  |  |  |  |  |  |
| G369 | 100.00 |  |  |  |  |  |  |  |  |  |  |  |
| G370 | 103.36 |  |  |  |  |  |  |  |  |  |  |  |
| G371 | 87.77 |  |  |  |  |  |  |  |  |  |  |  |
| G372 | 106.70 |  |  |  |  |  |  |  |  |  |  |  |
| G373 | 92.90 |  |  |  |  |  |  |  |  |  |  |  |
| G374 | 97.00 |  |  |  |  |  |  |  |  |  |  |  |
| G375 | 101.82 |  |  |  |  |  |  |  |  |  |  |  |
| G376 | 98.17 |  |  |  |  |  |  |  |  |  |  |  |
| G377 | 108.36 |  |  |  |  |  |  |  |  |  |  |  |
| G378 | 101.36 |  |  |  |  |  |  |  |  |  |  |  |
| G379 | 106.30 |  |  |  |  |  |  |  |  |  |  |  |
| G380 | 95.50 |  |  |  |  |  |  |  |  |  |  |  |
| G381 | 98.43 |  |  |  |  |  |  |  |  |  |  |  |
| G382 | 101.86 |  |  |  |  |  |  |  |  |  |  |  |
| G383 | 101.24 |  |  |  |  |  |  |  |  |  |  |  |
| G384 | 97.00 |  |  |  |  |  |  |  |  |  |  |  |
| G385 | 103.36 |  |  |  |  |  |  |  |  |  |  |  |
| G386 | 102.36 |  |  |  |  |  |  |  |  |  |  |  |
| G387 | 88.00 |  |  |  |  |  |  |  |  |  |  |  |
| G388 | 90.08 |  |  |  |  |  |  |  |  |  |  |  |
| G389 | 93.75 |  |  |  |  |  |  |  |  |  |  |  |
| G390 | 99.88 |  |  |  |  |  |  |  |  |  |  |  |
| G391 | 96.57 |  |  |  |  |  |  |  |  |  |  |  |
| G392 | 109.75 |  |  |  |  |  |  |  |  |  |  |  |
| G393 | 87.71 |  |  |  |  |  |  |  |  |  |  |  |
| G394 | 102.13 |  |  |  |  |  |  |  |  |  |  |  |
| G395 | 107.40 |  |  |  |  |  |  |  |  |  |  |  |
| G396 | 102.77 |  |  |  |  |  |  |  |  |  |  |  |
| G397 | 94.75 |  |  |  |  |  |  |  |  |  |  |  |
| G398 | 97.94 |  |  |  |  |  |  |  |  |  |  |  |
| G399 | 90.40 |  |  |  |  |  |  |  |  |  |  |  |
| G400 | 87.71 |  |  |  |  |  |  |  |  |  |  |  |
| G401 | 93.57 |  |  |  |  |  |  |  |  |  |  |  |
| G402 | 87.81 |  |  |  |  |  |  |  |  |  |  |  |
| G403 | 97.07 |  |  |  |  |  |  |  |  |  |  |  |
| G404 | 111.27 |  |  |  |  |  |  |  |  |  |  |  |
| G405 | 102.80 |  |  |  |  |  |  |  |  |  |  |  |
| G406 | 107.92 |  |  |  |  |  |  |  |  |  |  |  |
| G407 | 97.60 |  |  |  |  |  |  |  |  |  |  |  |
| G408 | 98.64 |  |  |  |  |  |  |  |  |  |  |  |
| G409 | 100.75 |  |  |  |  |  |  |  |  |  |  |  |
| G410 | 101.87 |  |  |  |  |  |  |  |  |  |  |  |
| G411 | 98.60 |  |  |  |  |  |  |  |  |  |  |  |
| G412 | 103.60 |  |  |  |  |  |  |  |  |  |  |  |
| G413 | 99.53 |  |  |  |  |  |  |  |  |  |  |  |
| G414 | 103.50 |  |  |  |  |  |  |  |  |  |  |  |
| G415 | 98.56 |  |  |  |  |  |  |  |  |  |  |  |
| G416 | 98.64 |  |  |  |  |  |  |  |  |  |  |  |
| G417 | 91.94 |  |  |  |  |  |  |  |  |  |  |  |
| G418 | 97.47 |  |  |  |  |  |  |  |  |  |  |  |
| G419 | 92.80 |  |  |  |  |  |  |  |  |  |  |  |
| G420 | 97.75 |  |  |  |  |  |  |  |  |  |  |  |
| G421 | 97.85 |  |  |  |  |  |  |  |  |  |  |  |
| G422 | 96.85 |  |  |  |  |  |  |  |  |  |  |  |
| G423 | 98.33 |  |  |  |  |  |  |  |  |  |  |  |
| G424 | 94.69 |  |  |  |  |  |  |  |  |  |  |  |
| G425 | 96.88 |  |  |  |  |  |  |  |  |  |  |  |
| G426 | 88.06 |  |  |  |  |  |  |  |  |  |  |  |
| G427 | 98.45 |  |  |  |  |  |  |  |  |  |  |  |
| G428 | 90.88 |  |  |  |  |  |  |  |  |  |  |  |
| G429 | 105.92 |  |  |  |  |  |  |  |  |  |  |  |
| G430 | 106.54 |  |  |  |  |  |  |  |  |  |  |  |
| G431 | 96.50 |  |  |  |  |  |  |  |  |  |  |  |
| G432 | 97.63 |  |  |  |  |  |  |  |  |  |  |  |
| G433 | 106.07 |  |  |  |  |  |  |  |  |  |  |  |
| G434 | 106.50 |  |  |  |  |  |  |  |  |  |  |  |
| G435 | 105.71 |  |  |  |  |  |  |  |  |  |  |  |
| G436 | 97.22 |  |  |  |  |  |  |  |  |  |  |  |
| G437 | 102.63 |  |  |  |  |  |  |  |  |  |  |  |
| G438 | 102.00 |  |  |  |  |  |  |  |  |  |  |  |
| G439 | 99.50 |  |  |  |  |  |  |  |  |  |  |  |
| G440 | 98.59 |  |  |  |  |  |  |  |  |  |  |  |
| G441 | 108.82 |  |  |  |  |  |  |  |  |  |  |  |
| G442 | 96.27 |  |  |  |  |  |  |  |  |  |  |  |
| G443 | 101.00 |  |  |  |  |  |  |  |  |  |  |  |
| G444 | 106.64 |  |  |  |  |  |  |  |  |  |  |  |
| G445 | 90.85 |  |  |  |  |  |  |  |  |  |  |  |
| G446 | 106.17 |  |  |  |  |  |  |  |  |  |  |  |
| G447 | 99.67 |  |  |  |  |  |  |  |  |  |  |  |
| G448 | 102.27 |  |  |  |  |  |  |  |  |  |  |  |
| G449 | 89.78 |  |  |  |  |  |  |  |  |  |  |  |
| G450 | 94.60 |  |  |  |  |  |  |  |  |  |  |  |
| G451 | 102.39 |  |  |  |  |  |  |  |  |  |  |  |
| G452 | 93.93 |  |  |  |  |  |  |  |  |  |  |  |
| G453 | 102.00 |  |  |  |  |  |  |  |  |  |  |  |
| G454 | 92.73 |  |  |  |  |  |  |  |  |  |  |  |
| G455 | 97.33 |  |  |  |  |  |  |  |  |  |  |  |
| G456 | 99.42 |  |  |  |  |  |  |  |  |  |  |  |
| G457 | 100.79 |  |  |  |  |  |  |  |  |  |  |  |
| G458 | 97.69 |  |  |  |  |  |  |  |  |  |  |  |
| G459 | 100.32 |  |  |  |  |  |  |  |  |  |  |  |
| G460 | 94.63 |  |  |  |  |  |  |  |  |  |  |  |
| G461 | 113.70 |  |  |  |  |  |  |  |  |  |  |  |
| G462 | 102.69 |  |  |  |  |  |  |  |  |  |  |  |
| G463 | 98.31 |  |  |  |  |  |  |  |  |  |  |  |
| G464 | 107.82 |  |  |  |  |  |  |  |  |  |  |  |
| G465 | 100.47 |  |  |  |  |  |  |  |  |  |  |  |
| G466 | 98.27 |  |  |  |  |  |  |  |  |  |  |  |
| G467 | 104.00 |  |  |  |  |  |  |  |  |  |  |  |
| G468 | 102.40 |  |  |  |  |  |  |  |  |  |  |  |
| G469 | 102.00 |  |  |  |  |  |  |  |  |  |  |  |
