# Supplementary material for: Mapping QTLs for anaerobic tolerance at germination and bud stages using new high density genetic map of rice
Source: Front Plant Sci. 2022 Oct 17;13:985080. doi: 10.3389/fpls.2022.985080 (PMC9618957; doi:10.3389/fpls.2022.985080)
Supplement: Supplementary file 7 [file Table_4.docx]

| **Supplementary Table S4** Number of elite alleles within each recombinant inbred line. | | |
| --- | --- | --- |
| Elite alleles | No. of RILs | RILs |
| 1 | 1 | G486 |
| 2 | 7 | G323,G544,G517,G399,G496,G372,G325 |
| 3 | 4 | G343,G471,G393,G393 |
| 4 | 15 | G287,G318,G421,G498,G532,G293,G295,G537,G405,G489,G403,G522,G513,G349,G500 |
| 5 | 18 | G276,G375,G465,G436,G302,G413,G410,G526,G331,G340,G456,G538,G367,G385,G409,G483,G479,G435 |
| 6 | 27 | G382,G362,G464,G447,G488,G419,G426,G519,G415,G383,G395,G508,G533,G311,G478,G417,G322,G376,G394,G329,G525,G423,G304,G490,G386,G350,G487 |
| 7 | 35 | G277,G296,G347,G392,G459,G374,G422,G352,G414,G308,G303,G506,G290,G497,G313,G280,G448,G342,G473,G286,G520,G512,G299,G346,G518,G529,G360,G452,G438,G301,G359,G381,G357,G475,G492 |
| 8 | 30 | G333,G339,G493,G310,G466,G540,G388,G468,G341,G396,G425,G298,G361,G432,G358,G441,G446,G453,G444,G305,G397,G454,G371,G429,G337,G472,G320,G353,G411,G462 |
| 9 | 28 | G338,G363,G387,G300,G412,G495,G389,G443,G390,G402,G545,G440,G369,G408,G427,G351,G527,G510,G365,G324,G514,G477,G516,G401,G321,G504,G485,G470 |
| 10 | 37 | G288,G521,G373,G499,G494,G458,G507,G407,G297,G420,G406,G278,G312,G306,G307,G345,G391,G523,G433,G314,G550,G334,G316,G531,G543,G384,G482,G404,G467,G416,G355,G380,G431,G292,G505,G400,G463 |
| 11 | 26 | G455,G445,G442,G279,G469,G546,G378,G437,G457,G291,G282,G548,G534,G285,G368,G541,G481,G344,G332,G319,G330,G294,G480,G379,G450,G317 |
| 12 | 16 | G315,G461,G328,G377,G281,G542,G449,G327,G326,G283,G524,G366,G476,G451,G430,G336 |
| 13 | 16 | G289,G284,G398,G528,G501,G549,G348,G434,G509,G474,G515,G356,G335,G503,G502,G309 |
| 14 | 8 | G530,G511,G364,G539,G418,G370,G484,G535 |
| 15 | 3 | G354,G439,G547 |
| 16 | 4 | G428,G491,G424,G460 |
