# Supplementary material for: Mapping QTLs for anaerobic tolerance at germination and bud stages using new high density genetic map of rice
Source: Front Plant Sci. 2022 Oct 17;13:985080. doi: 10.3389/fpls.2022.985080 (PMC9618957; doi:10.3389/fpls.2022.985080)
Supplement: Supplementary file 8 [file Table_5.docx]

| **Supplementary Table S5** Information on approximately 103 genes at locus 3. | | | | | |  |  |  |  |  |  |  |  |  |  |  |  |  |  |  |  |  |  |  |  |  |
| --- | --- | --- | --- | --- | --- | --- | --- | --- | --- | --- | --- | --- | --- | --- | --- | --- | --- | --- | --- | --- | --- | --- | --- | --- | --- | --- |
| MSU 7 ID | Lasanthi-Kudahettige *et al.* 2007 | | | Narsai et al., 2009 | | | | | | | | | | | | | | | | | | | Hsu and Tung, 2017 | | | Description |
|  | Probe signal intensity (GCRMA) | | Fold (nX) | Probe signal intensity (GCRMA) | | | | | | | | | | | | | Fold (nX)-1h | Fold (nX)-3h | Fold (nX)-12h | Fold (nX)-24h | Fold (nX)-27h | Fold (nX)-30h | Expression (base Mean) | | Fold (nX) |  |
|  | Control | Anoxia |  | 0h | Control-1h | Control-3h | Control-12h | Control-24h | Control-27h | Control-30h | Anoxia-1h | Anoxia-3h | Anoxia-12h | Anoxia-24h | Anoxia-27h | Anoxia-30h |  |  |  |  |  |  | Control group | Anoxia group |  |  |
| LOC_Os02g44108 | 3108.36 | 976.57 | -3.18 | 6.94 | 5.99 | 6.48 | 1271.94 | 3333.61 | 5058.32 | 5760.80 | 6.85 | 5.92 | 429.80 | 491.71 | 595.94 | 919.87 | 1.14 | -1.09 | -2.96 | -6.78 | -8.49 | -6.26 | 1374.83 | 2704.05 | 1.97 | expansin precursor, putative, expressed |
| LOC_Os02g44111 | 62.89 | 65.52 | 1.04 | 87.77 | 69.80 | 99.66 | 111.65 | 78.59 | 129.38 | 111.22 | 64.66 | 102.22 | 99.17 | 83.89 | 152.34 | 138.59 | -1.08 | 1.03 | -1.13 | 1.07 | 1.18 | 1.25 | 317.74 | 377.79 | 1.19 | sec20 domain containing protein, expressed |
| LOC_Os02g44120 |  |  |  |  |  |  |  |  |  |  |  |  |  |  |  |  |  |  |  |  |  |  | 0.01 | 2.27 | 227.20 | ZOS2-13 - C2H2 zinc finger protein, expressed |
| LOC_Os02g44124 |  |  |  |  |  |  |  |  |  |  |  |  |  |  |  |  |  |  |  |  |  |  |  |  |  | retrotransposon protein, putative, unclassified, expressed |
| LOC_Os02g44130 |  |  |  | 5.11 | 5.07 | 5.01 | 5.03 | 5.09 | 5.18 | 5.04 | 5.08 | 5.01 | 5.19 | 7.45 | 9.11 | 11.53 | 1.00 | -1.00 | 1.03 | 1.46 | 1.76 | 2.29 | 10.61 | 6.06 | -1.75 | ZOS2-14 - C2H2 zinc finger protein, expressed |
| LOC_Os02g44132 | 90.99 | 77.82 | -1.17 | 115.71 | 117.25 | 207.93 | 196.66 | 176.07 | 230.24 | 233.43 | 124.54 | 217.56 | 206.38 | 240.85 | 316.23 | 335.34 | 1.06 | 1.05 | 1.05 | 1.37 | 1.37 | 1.44 | 266.70 | 304.58 | 1.14 | expressed protein |
| LOC_Os02g44134 |  |  |  |  |  |  |  |  |  |  |  |  |  |  |  |  |  |  |  |  |  |  | 0.01 | 0.57 | 56.50 | thioesterase family protein, putative, expressed |
| LOC_Os02g44136 |  |  |  |  |  |  |  |  |  |  |  |  |  |  |  |  |  |  |  |  |  |  | 0.56 | 0.01 | -56.24 | retrotransposon protein, putative, Ty1-copia subclass, expressed |
| LOC_Os02g44140 |  |  |  |  |  |  |  |  |  |  |  |  |  |  |  |  |  |  |  |  |  |  |  |  |  | retrotransposon protein, putative, Ty1-copia subclass, expressed |
| LOC_Os02g44150 |  |  |  |  |  |  |  |  |  |  |  |  |  |  |  |  |  |  |  |  |  |  |  |  |  | retrotransposon protein, putative, Ty1-copia subclass |
| LOC_Os02g44155 |  |  |  |  |  |  |  |  |  |  |  |  |  |  |  |  |  |  |  |  |  |  | 227.86 | 140.46 | -1.62 | expressed protein |
| LOC_Os02g44160 |  |  |  |  |  |  |  |  |  |  |  |  |  |  |  |  |  |  |  |  |  |  | 4.76 | 7.23 | 1.52 | retrotransposon protein, putative, Ty3-gypsy subclass, expressed |
| LOC_Os02g44170 |  |  |  |  |  |  |  |  |  |  |  |  |  |  |  |  |  |  |  |  |  |  |  |  |  | retrotransposon protein, putative, Ty3-gypsy subclass, expressed |
| LOC_Os02g44180 |  |  |  |  |  |  |  |  |  |  |  |  |  |  |  |  |  |  |  |  |  |  | 2.37 | 0.57 | -4.14 | retrotransposon protein, putative, Ty3-gypsy subclass, expressed |
| LOC_Os02g44190 |  |  |  |  |  |  |  |  |  |  |  |  |  |  |  |  |  |  |  |  |  |  | 0.01 | 0.10 | 9.87 | retrotransposon protein, putative, Ty3-gypsy subclass |
| LOC_Os02g44200 |  |  |  |  |  |  |  |  |  |  |  |  |  |  |  |  |  |  |  |  |  |  | 0.71 | 0.12 | -5.76 | thioesterase family protein, putative, expressed |
| LOC_Os02g44210 |  |  |  |  |  |  |  |  |  |  |  |  |  |  |  |  |  |  |  |  |  |  |  |  |  | expressed protein |
| LOC_Os02g44220 | 50.55 | 38.21 | -1.32 | 14.81 | 14.63 | 14.28 | 15.16 | 13.33 | 32.82 | 33.67 | 15.30 | 15.46 | 14.83 | 13.97 | 33.39 | 35.82 | 1.05 | 1.08 | -1.02 | 1.05 | 1.02 | 1.06 | 842.80 | 809.49 | -1.04 | peroxisomal biogenesis factor 19, putative, expressed |
| LOC_Os02g44235 |  |  |  |  |  |  |  |  |  |  |  |  |  |  |  |  |  |  |  |  |  |  | 13.66 | 8.35 | -1.64 | expressed protein |
| LOC_Os02g44230 | 785.16 | 3293.40 | 4.19 | 12.96 | 16.22 | 99.13 | 38.71 | 76.25 | 146.54 | 190.90 | 10.58 | 157.05 | 1194.22 | 2424.60 | 4579.99 | 4792.17 | -1.53 | 1.58 | 30.85 | 31.80 | 31.25 | 25.10 | 1133.46 | 5174.92 | 4.57 | CPuORF22 - conserved peptide uORF-containing transcript, expressed |
| LOC_Os02g44240 |  |  |  |  |  |  |  |  |  |  |  |  |  |  |  |  |  |  |  |  |  |  |  |  |  | expressed protein |
| LOC_Os02g44250 |  |  |  | 9.11 | 9.33 | 9.63 | 8.99 | 8.79 | 10.70 | 10.55 | 9.35 | 13.04 | 11.58 | 12.32 | 11.61 | 11.16 | 1.00 | 1.35 | 1.29 | 1.40 | 1.09 | 1.06 | 46.64 | 51.78 | 1.11 | expressed protein |
| LOC_Os02g44260 |  |  |  |  |  |  |  |  |  |  |  |  |  |  |  |  |  |  |  |  |  |  | 0.26 | 0.10 | -2.64 | zinc-binding protein, putative, expressed |
| LOC_Os02g44270 |  |  |  | 24.19 | 25.52 | 13.06 | 7.27 | 5.96 | 4.29 | 4.20 | 36.18 | 20.71 | 6.38 | 6.04 | 4.24 | 4.31 | 1.42 | 1.59 | -1.14 | 1.01 | -1.01 | 1.03 | 66.70 | 67.62 | 1.01 | expressed protein |
| LOC_Os02g44280 |  |  |  | 6.32 | 6.58 | 6.41 | 6.90 | 8.00 | 15.29 | 13.62 | 6.42 | 6.21 | 7.17 | 7.63 | 12.12 | 13.36 | -1.03 | -1.03 | 1.04 | -1.05 | -1.26 | -1.02 | 21.64 | 15.50 | -1.40 | zinc finger protein, putative, expressed |
| LOC_Os02g44290 |  |  |  | 11.50 | 9.36 | 9.79 | 11.90 | 10.44 | 17.38 | 16.43 | 9.85 | 8.93 | 14.44 | 10.44 | 17.26 | 15.94 | 1.05 | -1.10 | 1.21 | -1.00 | -1.01 | -1.03 | 191.19 | 183.28 | -1.04 | phosphatase, putative, expressed |
| LOC_Os02g44300 | 19.78 | 12.32 | -1.61 | 7.25 | 7.23 | 7.04 | 7.00 | 9.21 | 12.70 | 16.81 | 7.10 | 6.99 | 7.04 | 7.04 | 9.15 | 9.24 | -1.02 | -1.01 | 1.01 | -1.31 | -1.39 | -1.82 | 155.85 | 170.56 | 1.09 | MSP domain containing protein, expressed |
| LOC_Os02g44310 | 19.39 | 136.03 | 7.02 | 8.94 | 5.39 | 5.20 | 779.69 | 2716.40 | 2874.25 | 3107.30 | 5.30 | 5.21 | 43.12 | 187.70 | 226.57 | 210.51 | -1.02 | 1.00 | -18.08 | -14.47 | -12.69 | -14.76 | 215.47 | 520.77 | 2.42 | LTPL112 - Protease inhibitor/seed storage/LTP family protein precursor, expressed |
| LOC_Os02g44320 | 139.03 | 61.64 | -2.26 | 4.69 | 4.67 | 4.61 | 8.84 | 400.50 | 351.55 | 691.85 | 4.64 | 4.59 | 4.62 | 17.14 | 52.61 | 74.25 | -1.01 | -1.00 | -1.91 | -23.37 | -6.68 | -9.32 | 24250.53 | 9185.23 | -2.64 | LTPL113 - Protease inhibitor/seed storage/LTP family protein precursor, expressed |
| LOC_Os02g44330 | 100.18 | 109.23 | 1.09 | 75.86 | 90.15 | 95.41 | 340.70 | 338.06 | 515.18 | 497.19 | 105.98 | 103.36 | 473.93 | 464.42 | 617.30 | 624.67 | 1.18 | 1.08 | 1.39 | 1.37 | 1.20 | 1.26 | 690.19 | 471.53 | -1.46 | rho guanine nucleotide exchange factor, putative, expressed |
| LOC_Os02g44340 |  |  |  |  |  |  |  |  |  |  |  |  |  |  |  |  |  |  |  |  |  |  | 3.91 | 7.44 | 1.90 | expressed protein |
| LOC_Os02g44350 |  |  |  |  |  |  |  |  |  |  |  |  |  |  |  |  |  |  |  |  |  |  | 0.32 | 0.41 | 1.27 | retrotransposon protein, putative, unclassified, expressed |
| LOC_Os02g44360 | 46.12 | 45.44 | -1.01 | 9.92 | 38.49 | 84.44 | 225.57 | 207.19 | 198.91 | 189.21 | 12.49 | 76.77 | 187.81 | 174.53 | 202.66 | 212.85 | -3.08 | -1.10 | -1.20 | -1.19 | 1.02 | 1.12 | 1087.92 | 1178.63 | 1.08 | scarecrow transcription factor family protein, putative, expressed |
| LOC_Os02g44370 | 310.71 | 178.73 | -1.74 | 55.55 | 84.27 | 303.91 | 852.28 | 872.33 | 757.17 | 762.49 | 65.21 | 136.72 | 664.16 | 671.24 | 699.39 | 702.23 | -1.29 | -2.22 | -1.28 | -1.30 | -1.08 | -1.09 | 1189.97 | 1387.97 | 1.17 | scarecrow, putative, expressed |
| LOC_Os02g44380 |  |  |  | 27.38 | 16.94 | 14.32 | 27.96 | 22.79 | 22.53 | 23.84 | 27.75 | 16.75 | 20.52 | 38.74 | 35.59 | 30.56 | 1.64 | 1.17 | -1.36 | 1.70 | 1.58 | 1.28 | 295.57 | 391.29 | 1.32 | WD domain containing protein, putative, expressed |
| LOC_Os02g44390 |  |  |  |  |  |  |  |  |  |  |  |  |  |  |  |  |  |  |  |  |  |  |  |  |  | retrotransposon protein, putative, unclassified, expressed |
| LOC_Os02g44400 |  |  |  |  |  |  |  |  |  |  |  |  |  |  |  |  |  |  |  |  |  |  |  |  |  | transposon protein, putative, unclassified, expressed |
| LOC_Os02g44410 |  |  |  |  |  |  |  |  |  |  |  |  |  |  |  |  |  |  |  |  |  |  |  |  |  | retrotransposon protein, putative, unclassified, expressed |
| LOC_Os02g44420 |  |  |  |  |  |  |  |  |  |  |  |  |  |  |  |  |  |  |  |  |  |  |  |  |  | conserved hypothetical protein |
| LOC_Os02g44430 |  |  |  |  |  |  |  |  |  |  |  |  |  |  |  |  |  |  |  |  |  |  | 1.86 | 4.03 | 2.17 | transposon protein, putative, unclassified, expressed |
| LOC_Os02g44440 |  |  |  |  |  |  |  |  |  |  |  |  |  |  |  |  |  |  |  |  |  |  | 0.01 | 0.12 | 12.28 | retrotransposon protein, putative, unclassified, expressed |
| LOC_Os02g44450 |  |  |  |  |  |  |  |  |  |  |  |  |  |  |  |  |  |  |  |  |  |  | 0.14 | 0.01 | -13.89 | retrotransposon protein, putative, unclassified |
| LOC_Os02g44460 |  |  |  |  |  |  |  |  |  |  |  |  |  |  |  |  |  |  |  |  |  |  |  |  |  | retrotransposon protein, putative, unclassified, expressed |
| LOC_Os02g44470 |  |  |  | 5.33 | 5.69 | 6.22 | 328.69 | 33.00 | 43.18 | 18.02 | 4.95 | 5.48 | 8.13 | 6.37 | 7.61 | 6.36 | -1.15 | -1.14 | -40.43 | -5.18 | -5.67 | -2.83 | 19.30 | 21.92 | 1.14 | actin-depolymerizing factor, putative, expressed |
| LOC_Os02g44480 |  |  |  | 5.90 | 5.79 | 5.75 | 6.43 | 5.90 | 7.06 | 6.78 | 5.77 | 5.70 | 6.01 | 5.91 | 6.79 | 6.70 | -1.00 | -1.01 | -1.07 | 1.00 | -1.04 | -1.01 | 26.27 | 37.81 | 1.44 | pentatricopeptide, putative, expressed |
| LOC_Os02g44490 |  |  |  |  |  |  |  |  |  |  |  |  |  |  |  |  |  |  |  |  |  |  | 1.33 | 1.93 | 1.45 | anthranilate phosphoribosyltransferase, putative, expressed |
| LOC_Os02g44500 | 740.75 | 200.31 | -3.70 | 1767.70 | 1866.92 | 1697.72 | 1086.52 | 597.74 | 607.53 | 630.22 | 1825.48 | 1653.40 | 708.29 | 268.55 | 311.34 | 300.89 | -1.02 | -1.03 | -1.53 | -2.23 | -1.95 | -2.09 | 1502.49 | 1746.30 | 1.16 | glutathione peroxidase, putative, expressed |
| LOC_Os02g44510 | 262.55 | 112.59 | -2.33 | 644.73 | 552.41 | 324.33 | 877.75 | 735.49 | 741.68 | 702.65 | 552.58 | 292.45 | 736.90 | 591.92 | 657.27 | 613.44 | 1.00 | -1.11 | -1.19 | -1.24 | -1.13 | -1.15 | 3983.08 | 3429.43 | -1.16 | UDP-glucose glycoprotein glucosyltransferase 1 precursor, putative, expressed |
| LOC_Os02g44520 | 320.48 | 255.61 | -1.25 | 95.79 | 55.54 | 44.73 | 320.02 | 258.01 | 297.94 | 286.69 | 73.56 | 48.41 | 200.55 | 251.82 | 240.48 | 210.98 | 1.32 | 1.08 | -1.60 | -1.02 | -1.24 | -1.36 | 3327.67 | 2847.42 | -1.17 | OsSub19 - Putative Subtilisin homologue, expressed |
| LOC_Os02g44530 |  |  |  |  |  |  |  |  |  |  |  |  |  |  |  |  |  |  |  |  |  |  | 5.22 | 19.47 | 3.73 | expressed protein |
| LOC_Os02g44540 | 120.42 | 76.76 | -1.57 | 78.40 | 65.52 | 175.08 | 197.04 | 140.94 | 208.25 | 215.62 | 75.69 | 72.25 | 251.79 | 186.50 | 304.99 | 363.66 | 1.16 | -2.42 | 1.28 | 1.32 | 1.46 | 1.69 | 1788.90 | 1400.49 | -1.28 | expressed protein |
| LOC_Os02g44550 | 20.19 | 38.29 | 1.90 | 36.67 | 44.10 | 107.63 | 61.92 | 40.73 | 29.00 | 28.12 | 28.29 | 73.97 | 68.09 | 57.34 | 42.43 | 50.50 | -1.56 | -1.46 | 1.10 | 1.41 | 1.46 | 1.80 | 656.24 | 685.71 | 1.04 | NADP-dependent malic enzyme, putative, expressed |
| LOC_Os02g44560 |  |  |  |  |  |  |  |  |  |  |  |  |  |  |  |  |  |  |  |  |  |  |  |  |  | C2 domain containing protein, putative, expressed |
| LOC_Os02g44570 | 20.13 | 20.17 | 1.00 | 99.75 | 91.36 | 63.41 | 85.19 | 83.13 | 87.90 | 86.73 | 90.74 | 79.29 | 103.17 | 103.83 | 135.92 | 141.39 | -1.01 | 1.25 | 1.21 | 1.25 | 1.55 | 1.63 | 631.64 | 591.66 | -1.07 | mitochondrial carrier protein, putative, expressed |
| LOC_Os02g44590 |  |  |  |  |  |  |  |  |  |  |  |  |  |  |  |  |  |  |  |  |  |  | 1.92 | 4.43 | 2.31 | OsSub20 - Putative Subtilisin homologue, expressed |
| LOC_Os02g44599 |  |  |  | 9.56 | 9.23 | 7.97 | 14.74 | 10.47 | 26.38 | 29.22 | 9.85 | 7.21 | 16.79 | 11.63 | 50.81 | 52.58 | 1.07 | -1.10 | 1.14 | 1.11 | 1.93 | 1.80 | 501.57 | 673.12 | 1.34 | expressed protein |
| LOC_Os02g44610 |  |  |  |  |  |  |  |  |  |  |  |  |  |  |  |  |  |  |  |  |  |  | 214.21 | 219.50 | 1.02 | protein kinase, putative, expressed |
| LOC_Os02g44620 |  |  |  | 5.51 | 5.52 | 5.36 | 5.40 | 5.41 | 5.77 | 5.51 | 5.46 | 5.41 | 5.48 | 5.43 | 5.48 | 5.42 | -1.01 | 1.01 | 1.02 | 1.00 | -1.05 | -1.02 | 0.14 | 0.34 | 2.45 | expressed protein |
| LOC_Os02g44630 | 4880.61 | 2671.09 | -1.83 | 3075.54 | 2596.25 | 2245.53 | 3938.21 | 5897.07 | 4754.47 | 5462.83 | 2860.39 | 2541.92 | 2746.10 | 4197.79 | 4095.59 | 4722.70 | 1.10 | 1.13 | -1.43 | -1.40 | -1.16 | -1.16 | 13943.07 | 16728.28 | 1.20 | aquaporin protein, putative, expressed |
| LOC_Os02g44642 | 137.26 | 152.32 | 1.11 | 116.32 | 94.32 | 470.21 | 535.21 | 435.49 | 582.91 | 561.91 | 95.26 | 248.46 | 649.52 | 470.90 | 685.28 | 622.05 | 1.01 | -1.89 | 1.21 | 1.08 | 1.18 | 1.11 | 1576.74 | 1625.69 | 1.03 | STE_MEKK_ste11_MAP3K.10 - STE kinases include homologs to sterile 7, sterile 11 and sterile 20 from yeast, expressed |
| LOC_Os02g44654 | 34.62 | 43.99 | 1.27 | 7.37 | 7.71 | 8.05 | 125.40 | 243.79 | 178.14 | 205.62 | 7.52 | 7.20 | 83.75 | 77.01 | 120.10 | 102.50 | -1.02 | -1.12 | -1.50 | -3.17 | -1.48 | -2.01 | 1422.25 | 1120.16 | -1.27 | cytochrome P450, putative, expressed |
| LOC_Os02g44670 |  |  |  | 4.51 | 4.50 | 4.44 | 4.44 | 6.02 | 4.67 | 5.01 | 4.46 | 4.45 | 4.46 | 4.46 | 5.14 | 4.56 | -1.01 | 1.00 | 1.01 | -1.35 | 1.10 | -1.10 | 177.74 | 183.63 | 1.03 | harpin-induced protein 1 domain containing protein, expressed |
| LOC_Os02g44680 |  |  |  | 4.42 | 4.41 | 4.05 | 16.78 | 14.42 | 8.46 | 11.24 | 4.38 | 4.20 | 4.86 | 4.47 | 6.51 | 6.78 | -1.01 | 1.04 | -3.45 | -3.23 | -1.30 | -1.66 | 114.55 | 84.47 | -1.36 | allantoin permease, putative, expressed |
| LOC_Os02g44690 | 663.28 | 230.04 | -2.88 | 1916.94 | 2017.87 | 1881.28 | 2002.30 | 1809.60 | 2203.58 | 2146.44 | 2072.33 | 1842.71 | 2029.02 | 1673.93 | 1781.42 | 1666.46 | 1.03 | -1.02 | 1.01 | -1.08 | -1.24 | -1.29 | 1558.20 | 1230.87 | -1.27 | HSPC171 protein, putative, expressed |
| LOC_Os02g44700 |  |  |  |  |  |  |  |  |  |  |  |  |  |  |  |  |  |  |  |  |  |  | 2.23 | 0.97 | -2.30 | zinc finger, C3HC4 type domain containing protein, expressed |
| LOC_Os02g44704 |  |  |  | 74.00 | 75.18 | 129.27 | 134.14 | 147.57 | 208.70 | 193.46 | 82.70 | 100.48 | 226.35 | 184.00 | 224.03 | 214.46 | 1.10 | -1.29 | 1.69 | 1.25 | 1.07 | 1.11 | 459.17 | 313.86 | -1.46 | LYR motif containing protein, putative, expressed |
| LOC_Os02g44710 | 1302.24 | 924.18 | -1.41 | 623.49 | 2069.19 | 2373.12 | 2002.32 | 2936.50 | 2333.76 | 2127.70 | 895.76 | 2159.80 | 2434.06 | 1970.36 | 1962.39 | 1926.48 | -2.31 | -1.10 | 1.22 | -1.49 | -1.19 | -1.10 | 650.44 | 1132.69 | 1.74 | expressed protein |
| LOC_Os02g44720 | 521.40 | 1178.11 | 2.26 | 65.13 | 482.19 | 474.46 | 494.09 | 873.87 | 863.47 | 699.26 | 155.90 | 828.67 | 1149.30 | 1011.94 | 1127.74 | 1231.83 | -3.09 | 1.75 | 2.33 | 1.16 | 1.31 | 1.76 | 191.15 | 295.61 | 1.55 | expressed protein |
| LOC_Os02g44730 | 35.20 | 6.71 | -5.24 |  |  |  |  |  |  |  |  |  |  |  |  |  |  |  |  |  |  |  | 105.03 | 33.12 | -3.17 | tetracycline transporter protein, putative, expressed |
| LOC_Os02g44740 | 9.32 | 11.89 | 1.28 | 4.25 | 4.24 | 6.00 | 4.16 | 4.15 | 4.18 | 4.10 | 4.21 | 39.68 | 4.47 | 4.20 | 4.18 | 4.08 | -1.01 | 6.62 | 1.07 | 1.01 | 1.00 | -1.00 | 743.37 | 737.55 | -1.01 | expressed protein |
| LOC_Os02g44750 |  |  |  |  |  |  |  |  |  |  |  |  |  |  |  |  |  |  |  |  |  |  | 17.68 | 13.19 | -1.34 | expressed protein |
| LOC_Os02g44760 |  |  |  | 8.70 | 8.47 | 8.45 | 7.85 | 7.65 | 7.09 | 6.60 | 7.98 | 8.27 | 7.91 | 7.90 | 6.96 | 6.75 | -1.06 | -1.02 | 1.01 | 1.03 | -1.02 | 1.02 |  |  |  | hypothetical protein |
| LOC_Os02g44770 | 8.48 | 67.33 | 7.94 |  |  |  |  |  |  |  |  |  |  |  |  |  |  |  |  |  |  |  | 44.75 | 102.84 | 2.30 | uncharacterized mscS family protein, putative, expressed |
| LOC_Os02g44780 | 215.97 | 190.46 | -1.13 | 150.25 | 284.01 | 956.28 | 597.55 | 510.54 | 627.02 | 610.15 | 185.24 | 463.29 | 491.54 | 364.00 | 469.55 | 488.72 | -1.53 | -2.06 | -1.22 | -1.40 | -1.34 | -1.25 | 1053.85 | 772.33 | -1.36 | polyprenyl synthetase, putative, expressed |
| LOC_Os02g44800 |  |  |  |  |  |  |  |  |  |  |  |  |  |  |  |  |  |  |  |  |  |  |  |  |  | hypothetical protein |
| LOC_Os02g44810 | 59.42 | 138.17 | 2.33 | 542.31 | 532.18 | 530.03 | 516.17 | 431.26 | 616.56 | 620.97 | 548.22 | 507.27 | 535.05 | 525.35 | 703.39 | 651.16 | 1.03 | -1.04 | 1.04 | 1.22 | 1.14 | 1.05 | 526.02 | 490.70 | -1.07 | tRNA pseudouridine synthase family protein, putative, expressed |
| LOC_Os02g44820 | 105.84 | 439.68 | 4.15 | 6477.19 | 6408.20 | 3995.60 | 1171.55 | 1097.71 | 951.90 | 873.05 | 6243.35 | 5485.01 | 1635.35 | 796.33 | 501.97 | 629.29 | -1.03 | 1.37 | 1.40 | -1.38 | -1.90 | -1.39 | 439.11 | 350.17 | -1.25 | rho-GTPase-activating protein-related, putative, expressed |
| LOC_Os02g44830 |  |  |  |  |  |  |  |  |  |  |  |  |  |  |  |  |  |  |  |  |  |  | 0.57 | 0.62 | 1.09 | hypothetical protein |
| LOC_Os02g44840 |  |  |  |  |  |  |  |  |  |  |  |  |  |  |  |  |  |  |  |  |  |  | 23.16 | 17.27 | -1.34 | expressed protein |
| LOC_Os02g44850 |  |  |  |  |  |  |  |  |  |  |  |  |  |  |  |  |  |  |  |  |  |  | 5.63 | 5.27 | -1.07 | GDSL-like lipase/acylhydrolase, putative, expressed |
| LOC_Os02g44860 | 8.32 | 25.92 | 3.12 | 25.12 | 24.64 | 20.01 | 93.66 | 102.79 | 204.67 | 243.06 | 22.31 | 20.91 | 51.92 | 177.44 | 382.13 | 411.47 | -1.10 | 1.05 | -1.80 | 1.73 | 1.87 | 1.69 | 914.28 | 762.13 | -1.20 | GDSL-like lipase/acylhydrolase, putative, expressed |
| LOC_Os02g44870 | 1930.21 | 1233.37 | -1.56 | 4861.60 | 5477.37 | 4278.92 | 74.14 | 212.99 | 381.89 | 371.70 | 5593.08 | 4176.57 | 145.98 | 52.32 | 96.02 | 82.40 | 1.02 | -1.02 | 1.97 | -4.07 | -3.98 | -4.51 | 3825.81 | 5916.94 | 1.55 | dehydrin, putative, expressed |
| LOC_Os02g44880 | 515.63 | 236.34 | -2.18 |  |  |  |  |  |  |  |  |  |  |  |  |  |  |  |  |  |  |  | 157.30 | 282.16 | 1.79 | expressed protein |
| LOC_Os02g44890 | 23.40 | 35.43 | 1.51 | 48.90 | 47.14 | 38.86 | 102.01 | 74.71 | 106.57 | 86.45 | 58.05 | 46.62 | 87.25 | 111.94 | 119.52 | 135.74 | 1.23 | 1.20 | -1.17 | 1.50 | 1.12 | 1.57 | 206.03 | 185.36 | -1.11 | RNA-binding region RNP-1, putative, expressed |
| LOC_Os02g44900 | 23.44 | 29.21 | 1.25 | 185.21 | 353.52 | 303.10 | 60.85 | 81.20 | 99.22 | 110.56 | 233.23 | 501.51 | 97.97 | 133.32 | 202.56 | 197.53 | -1.52 | 1.65 | 1.61 | 1.64 | 2.04 | 1.79 | 148.98 | 156.31 | 1.05 | expressed protein |
| LOC_Os02g44910 | 24.70 | 11.65 | -2.12 | 5.33 | 5.34 | 5.22 | 5.73 | 5.65 | 5.56 | 5.35 | 5.32 | 5.23 | 5.34 | 5.32 | 5.23 | 5.19 | -1.00 | 1.00 | -1.07 | -1.06 | -1.06 | -1.03 | 329.65 | 327.53 | -1.01 | transmembrane protein, putative, expressed |
| LOC_Os02g44920 | 39.17 | 5.21 | -7.53 |  |  |  |  |  |  |  |  |  |  |  |  |  |  |  |  |  |  |  | 6.03 | 3.44 | -1.75 | protein kinase domain containing protein, expressed |
| LOC_Os02g44930 | 37.54 | 82.99 | 2.21 | 2515.43 | 2422.93 | 1838.32 | 634.17 | 589.88 | 849.35 | 819.05 | 2593.48 | 2303.56 | 535.85 | 457.94 | 568.74 | 524.24 | 1.07 | 1.25 | -1.18 | -1.29 | -1.49 | -1.56 | 2509.92 | 1540.73 | -1.63 | HMG1/2, putative, expressed |
| LOC_Os02g44940 | 503.86 | 167.73 | -3.00 | 6.65 | 6.47 | 6.02 | 6.11 | 6.09 | 5.24 | 5.08 | 6.43 | 6.11 | 6.10 | 6.10 | 5.07 | 5.13 | -1.01 | 1.02 | -1.00 | 1.00 | -1.03 | 1.01 | 1018.12 | 1168.49 | 1.15 | RALFL8 - Rapid ALkalinization Factor RALF family protein precursor, expressed |
| LOC_Os02g44950 |  |  |  |  |  |  |  |  |  |  |  |  |  |  |  |  |  |  |  |  |  |  | 0.01 | 0.09 | 8.97 | transposon protein, putative, unclassified |
| LOC_Os02g44970 |  |  |  |  |  |  |  |  |  |  |  |  |  |  |  |  |  |  |  |  |  |  | 66.83 | 65.32 | -1.02 | expressed protein |
| LOC_Os02g44975 |  |  |  |  |  |  |  |  |  |  |  |  |  |  |  |  |  |  |  |  |  |  | 7.36 | 14.59 | 1.98 | expressed protein |
| LOC_Os02g44980 | 621.51 | 352.49 | -1.76 | 108.07 | 220.60 | 313.83 | 165.50 | 83.47 | 49.33 | 71.10 | 105.25 | 218.87 | 23.20 | 30.47 | 24.80 | 25.99 | -2.10 | -1.43 | -7.13 | -2.74 | -1.99 | -2.74 | 3007.22 | 1464.19 | -2.05 | transmembrane amino acid transporter protein, putative, expressed |
| LOC_Os02g44990 | 222.68 | 747.58 | 3.36 | 284.88 | 367.72 | 190.99 | 11.53 | 7.12 | 10.90 | 10.27 | 349.48 | 359.27 | 45.51 | 27.11 | 21.78 | 29.15 | -1.05 | 1.88 | 3.95 | 3.81 | 2.00 | 2.84 | 230.40 | 290.13 | 1.26 | OsFBDUF13 - F-box and DUF domain containing protein, expressed |
| LOC_Os02g45000 | 487.74 | 171.20 | -2.85 | 2685.56 | 3345.85 | 1193.73 | 48.47 | 40.28 | 27.20 | 34.32 | 3020.68 | 2003.56 | 25.28 | 26.15 | 23.85 | 26.48 | -1.11 | 1.68 | -1.92 | -1.54 | -1.14 | -1.30 | 402.86 | 413.72 | 1.03 | expressed protein |
| LOC_Os02g45010 |  |  |  |  |  |  |  |  |  |  |  |  |  |  |  |  |  |  |  |  |  |  | 50.59 | 97.93 | 1.94 | ethylene-responsive protein related, putative, expressed |
| LOC_Os02g45020 |  |  |  |  |  |  |  |  |  |  |  |  |  |  |  |  |  |  |  |  |  |  | 0.24 | 0.43 | 1.74 | transposon protein, putative, CACTA, En/Spm sub-class, expressed |
| LOC_Os02g45030 |  |  |  |  |  |  |  |  |  |  |  |  |  |  |  |  |  |  |  |  |  |  | 0.01 | 1.96 | 196.33 | latency associated nuclear antigen, putative, expressed |
| LOC_Os02g45040 |  |  |  |  |  |  |  |  |  |  |  |  |  |  |  |  |  |  |  |  |  |  | 237.28 | 746.52 | 3.15 | retrotransposon protein, putative, unclassified, expressed |
| LOC_Os02g45054 | 25.59 | 54.47 | 2.13 | 181.75 | 206.59 | 445.75 | 258.28 | 216.64 | 202.79 | 190.16 | 230.83 | 263.40 | 234.09 | 246.84 | 219.67 | 204.03 | 1.12 | -1.69 | -1.10 | 1.14 | 1.08 | 1.07 | 2113.52 | 1271.42 | -1.66 | ZOS2-15 - C2H2 zinc finger protein, expressed |
| LOC_Os02g45070 | 168.82 | 256.88 | 1.52 | 202.89 | 173.79 | 188.90 | 202.16 | 233.23 | 278.71 | 285.03 | 187.51 | 149.69 | 185.54 | 292.84 | 420.35 | 430.70 | 1.08 | -1.26 | -1.09 | 1.26 | 1.51 | 1.51 | 3436.90 | 3741.12 | 1.09 | PINHEAD, putative, expressed |
| LOC_Os02g45080 |  |  |  | 4.86 | 4.82 | 4.76 | 4.84 | 4.92 | 5.10 | 5.05 | 4.81 | 4.75 | 4.80 | 4.79 | 4.86 | 4.77 | -1.00 | -1.00 | -1.01 | -1.03 | -1.05 | -1.06 | 3.60 | 10.01 | 2.78 | MYB family transcription factor, putative, expressed |
| LOC_Os02g45090 | 33.82 | 79.96 | 2.36 | 884.25 | 926.86 | 543.44 | 267.18 | 217.10 | 295.34 | 280.66 | 947.64 | 706.23 | 250.02 | 262.09 | 294.59 | 319.58 | 1.02 | 1.30 | -1.07 | 1.21 | -1.00 | 1.14 | 729.28 | 576.80 | -1.26 | expressed protein |
